# Alcohols electrooxidation coupled with H_2_ production at high current densities promoted by a cooperative catalyst

**DOI:** 10.1038/s41467-021-27806-3

**Published:** 2022-01-10

**Authors:** Zhenhua Li, Yifan Yan, Si-Min Xu, Hua Zhou, Ming Xu, Lina Ma, Mingfei Shao, Xianggui Kong, Bin Wang, Lirong Zheng, Haohong Duan

**Affiliations:** 1 State Key Laboratory of Chemical Resource Engineering, College of Chemistry, Beijing University of Chemical Technology, Beijing, 100029 China; 2 Department of Chemistry, Tsinghua University, Beijing, 100084 China; 3 Beijing Research Institute of Chemical Industry, Sinopec Group, Beijing, 100013 China; 4 Institute of High Energy Physics, the Chinese Academy of Sciences, Beijing, 100049 China

**Keywords:** Electrocatalysis, Nanoscale materials, Electrocatalysis

## Abstract

Electrochemical alcohols oxidation offers a promising approach to produce valuable chemicals and facilitate coupled H_2_ production. However, the corresponding current density is very low at moderate cell potential that substantially limits the overall productivity. Here we report the electrooxidation of benzyl alcohol coupled with H_2_ production at high current density (540 mA cm^−2^ at 1.5 V *vs*. RHE) over a cooperative catalyst of Au nanoparticles supported on cobalt oxyhydroxide nanosheets (Au/CoOOH). The absolute current can further reach 4.8 A at 2.0 V in a more realistic two-electrode membrane-free flow electrolyzer. Experimental combined with theoretical results indicate that the benzyl alcohol can be enriched at Au/CoOOH interface and oxidized by the electrophilic oxygen species (OH*) generated on CoOOH, leading to higher activity than pure Au. Based on the finding that the catalyst can be reversibly oxidized/reduced at anodic potential/open circuit, we design an intermittent potential (IP) strategy for long-term alcohol electrooxidation that achieves high current density (>250 mA cm^−2^) over 24 h with promoted productivity and decreased energy consumption.

## Abstract (web summary)

Electrochemical alcohol oxidation offers a promising approach to produce valuable chemicals that can be paired with fuel-producing reactions. Here, authors utilize gold and cobalt oxyhydroxide nanomaterials to obtain industrially-relevant electrolyzer current densities for benzyl alcohol oxidation.

## Introduction

Hydrogen (H_2_) is a promising energy carrier to replace fossil fuels that can address the environmental problems associated with global warming and alleviates the energy crisis. Electrocatalytic water splitting powered by clean energy (e.g., solar energy, wind) represents a green approach to produce H_2_^1,2^. However, this process still suffers from large overall electricity consumption stemming from high cell potential due to the sluggish four-electron transfer of anodic oxygen evolution reaction (OER)^3,4^. Developing anodic reactions with cell potential lower than OER is a promising way for fundamentally lowering the energy requirements for electrocatalytic H_2_ production. Such anode oxidation reactions have the additional benefit of producing value-added products from inexpensive industrial feedstocks or renewable biomass sources^5,6^. For reactions of this kind, applying large positive potential would unavoidably result in uncontrolled OER, therefore applying moderate potential is necessary to achieve high Faradaic efficiency (FE) toward target products^7^. Tremendous efforts have been devoted to developing electrocatalysts with improved catalytic activities towards various oxidation reactions^8–13^. Despite these efforts, the reported current density remains very low under moderate cell potential, for example, the electrooxidation of alcohols^8,9^, 5-hydroxymethyfurfural (HMF)^10,11^, primary amines^12^ and tetrahydroisoquinolines^13^ are mostly operated at current density lower than 200 mA cm^–2^. This would hamper the overall efficiency and profitability for industrial production of value-added products coupled with H_2_ evolution that requires high current density.

**Fig. 1:**
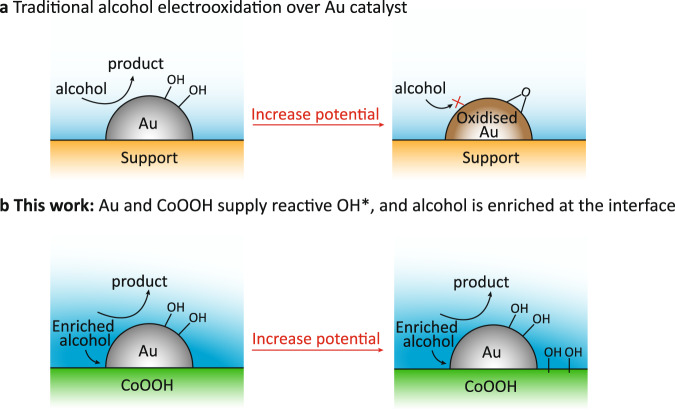
Schematic illustration of the AOR process. **a** Traditional AOR process over Au catalyst and its deactivation at high potential. **b** Alcohols enrichment and reactive OH* generation over Au/CoOOH, showing enhanced current density.

Among the types of anodic reactions, alcohol oxidation reactions (AORs) are particularly important for their wide applications in commodity chemicals production. For instance, the oxidation of benzyl alcohol produces benzoic acid, which is an important fine chemical used in synthetic fiber, resin, and antiseptic industries^14,15^. Oxidation of polyols (such as glycerol) affords corresponding ketones/aldehydes and formate, which can find applications as the degradable plastic monomer, dyestuffs and food additives^16–18^. Among the catalysts studied, Au is a promising material for AORs with high activity at low potential (<1.0 V vs. RHE)^19–22^. In a typical alcohol electrooxidation reaction over Au catalyst, the alcohol molecules tend to interact with Au to form adsorbed R–CH_2_O* species at a moderate potential, which is then oxidized by the in-situ formed electrophilic OH* species on Au (Au–OH) at higher potential (Fig. 1a), with following catalytic dehydrogenation of the beta-hydrogen (C−H_*β*_) of alcohol to give corresponding ketones and carboxylates. Recently, Koper and co-workers reported that the presence of adsorbed CO on Au (111) could strengthen OH adsorption on Au at low potential, which promotes C−H_*β*_ elimination and promote the AOR activity^20^. It was also reported that incorporation of Ni in AuNi alloys could facilitate the formation of Au−OH species and thus improve the glycerol electrooxidation activity^22^. However, the current density at low potential is still insufficient for industrially-relevant production that requires >300 mA cm^–2^. When the potential is further increased, the Au−OH species would be unavoidably oxidized to AuO_*x*_ that causes catalyst deactivation (Fig. 1a). As a result, it remains a huge challenge to develop Au catalysts for AORs operated at high current density.

Here we synthesized a cooperative catalyst of Au nanoparticles (NPs) supported on cobalt oxyhydroxide nanosheets (Au/CoOOH), which realizes the AORs coupled with H_2_ production at high current density. Specifically, the Au/CoOOH exhibits current density of 340 mA cm^−2^ at potential of 1.3 V vs. RHE in 1 M KOH with 0.1 M benzyl alcohol at room temperature (r.t.). The corresponding benzyl alcohol oxidation rate and H_2_ production rate reach 3.19 mmol cm^−2^ h^−1^ and 117.9 mL cm^−2^ h^−1^, respectively, at a constant potential of 1.3 V *vs*. RHE, which is 26- and 28-fold higher than that of Au. The current density can further reach 540 mA cm^−2^ at 1.5 V vs. RHE, representing the highest current density value reported so far at such low potential. The absolute current can further reach 4.8 A at 2.0 V in a more realistic two-electrode membrane-free flow electrolyzer, suggesting the potential of the catalyst to work under industrially-relevant conditions. Experiments integrated with spin-polarized density functional theory (DFT) studies reveal that the benzyl alcohol (in the form of alkoxide) is enriched at Au/CoOOH interface and oxidized by the electrophilic OH* generated on CoOOH with low reaction barrier, leading to higher activity than pure Au (Fig. 1b). The Au/CoOOH affords a broad substrate scope, including alcohols with alpha-*π* bond such as phenyl, C=C and C=O groups, showing 9~28-fold higher current density than Au. Based on the finding that catalyst can be reversibly oxidized/reduced at anodic potential/open circuit, we developed an intermittent potential (IP) strategy for long-term electrooxidation, achieving high current density (>250 mA cm^−2^) over 24 h. Compared with constant potential (CP) strategy, this IP strategy achieves 10- and 9-fold increment for benzyl alcohol oxidation and H_2_ production, respectively, together with 33% of electric energy saving at 300 mA cm^−2^.

## Results

### Screening of catalysts for benzyl alcohol oxidation

Note that the in-situ formed Au−OH species play a vital role for AORs over Au catalysts, but is easily oxidized to AuO_*x*_ at higher potential that causes deactivation. We sought to incorporate Au with a component that can supply electrophilic OH* for AORs. It is well-documented that transition metal oxyhydroxides (MOOHs) are capable of generating active oxygen species (OH* or O*) in an aqueous solution under certain anodic potential for oxidation of various organic substrates^23^. Hence, we expect that the MOOHs can cooperate with Au for AOR at higher potential. With this idea in mind, we synthesized Au supported on CoOOH and NiFeOOH, with these two MOOH being reported to produce active oxygen species at certain potentials^24,25^. Of note, the synthesized Au/CoOOH and Au/NiFeOOH shows similar Au mass loading, particle size and nanosheet array structure (Supplementary Fig. 1), thus the influence of catalyst’s morphology on the catalytic performance can be ruled out when making comparison. Their performances in benzyl alcohol electrooxidation were then evaluated.

**Fig. 2:**
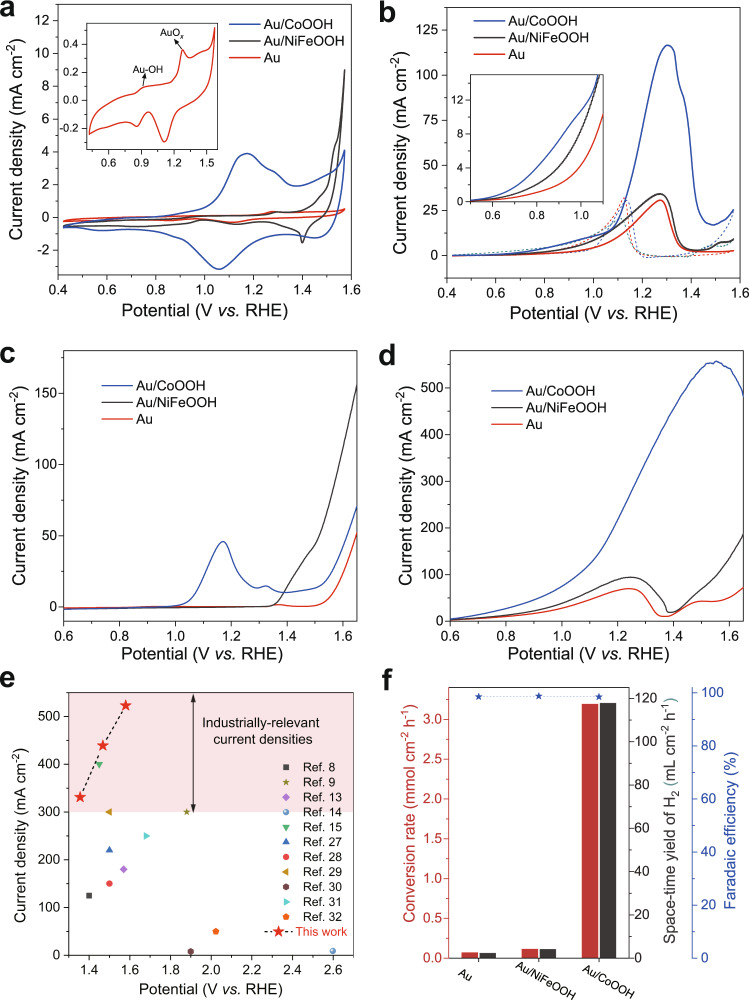
Benzyl alcohol electrooxidation over Au and Au/MOOHs catalysts. **a** CV curves of glassy carbon electrode supported Au, Au/CoOOH and Au/NiFeOOH catalysts in 1 M KOH (Inset: CV curve of Au). **b** CV curves in 1 M KOH with 0.1 M benzyl alcohol (Inset: local magnification of the CV curve). **c**, **d** LSV curves of Ni foam supported Au, Au/CoOOH, and Au/NiFeOOH catalysts at scan rate of 10 mV s^−1^ in (**c**) 1 M KOH and (**d**) 1 M KOH with 0.1 M benzyl alcohol at r.t. **e** Current densities of anodic oxidation reactions reported in the literatures and the benzyl alcohol oxidation in this work. **f** Benzyl alcohol conversion rate, H_2_ space-time yield and Faradaic efficiency over different catalysts coupled Pt cathode, in 1 M KOH with 0.1 M benzyl alcohol at 1.3 V vs. RHE.

We first investigated the redox properties of Au, Au/CoOOH and Au/NiFeOOH by cyclic voltammetry (CV) in 1 M KOH electrolyte. Glassy carbon electrode was used as the conductive substrate. As shown in Fig. 2a, Au exhibits two oxidation waves in the anodic direction (red curve in Fig. 2a and inset), which are attributed to the adsorption of OH^−^ to form Au−OH (from ~0.84 V vs. RHE) and its further oxidation to AuO_*x*_ (from 1.2 V to 1.35 V vs. RHE), in consistent with previous report^21^. For Au/CoOOH (blue curve in Fig. 2a), another oxidation peak was observed at ~1.15 V vs. RHE, at potential lower than the formation of AuO_*x*_, which can be assigned to the reversible oxidation of Co^2+^ to Co^3+^ in CoOOH^26^. Previous reports have demonstrated that Co^2+^ oxidation is accompanied by the formation of electrophilic OH*, which showed catalytic activity for nucleophilic alcohols oxidation.^23–25^ For Au/NiFeOOH (dark cyan curve in Fig. 2a), the oxidation of Ni^2+^ to Ni^3+^ with possible active oxygen species generation was observed at potential (~1.47 V *vs*. RHE) higher than AuO_*x*_ formation.

Figure 2b shows CV curves (with 0.1 M benzyl alcohol) of the samples on glassy carbon electrode. For Au in Fig. 2b, an oxidation current at ~0.6 V vs. RHE was observed, which is absent in the blank voltammetry in KOH (red curve in Fig. 2a). This is assigned to the adsorption of benzyl alcohol on Au in the form of alkoxide in an alkaline media. The oxidation current begins to prominently increase at potential higher than 0.8 V vs. RHE, suggesting the occurrence of alkoxide oxidation by Au−OH that is formed at the same potential region. The oxidation current begins to decay at ~1.3 V vs. RHE and then diminishes at ~1.4 V vs. RHE as a result of the formation of AuO_*x*_. For benzyl alcohol oxidation over Au/CoOOH (blue curve in Fig. 2b), the onset potential of the adsorption of benzyl alcohol is lower than that of Au. While the oxidation current of Au/CoOOH is only slightly higher than Au below ~1.15 V vs. RHE, it significantly increases beginning at ~1.15 V vs. RHE, at the same potential for the oxidation of Co^2+^ to Co^3+^ (blue curve in Fig. 2a). We speculate the increase of the current density stems from benzyl alcohol oxidation by the in-situ formed OH* on CoOOH. In comparison, Au/NiFeOOH (dark cyan curve in Fig. 2b) does not show obvious current enhancement. Another oxidation wave was observed at ~1.45 V vs. RHE over Au/NiFeOOH, which can be ascribed to benzyl alcohol oxidation catalyzed by NiFeOOH (Supplementary Fig. 2).

To gain higher current density, we then prepared Au, Au/NiFeOOH and Au/CoOOH supported on Ni foam (Supplementary Fig. 3). As shown in Fig. 2c, the LSV curves exhibit similar trend as in Fig. 2a, with Au/CoOOH and Au/NiFeOOH showing oxidation peaks centred at 1.19 and 1.45 V vs. RHE, respectively. For Au, no obvious oxidation peaks were observed except the weak signal from nickel foam. After 0.1 M benzyl alcohol was introduced into the KOH electrolyte, oxidation peaks were observed beginning at ~0.8 V vs. RHE over all the samples, which is assigned to the benzyl alcohol oxidation catalysed by Au−OH species (Fig. 2d), in consistent with the CV results using glassy carbon electrodes (Fig. 2b). Au/CoOOH exhibits higher current density than Au and Au/NiFeOOH after 1.2 V vs. RHE, at which potential CoOOH is capable of generating OH* that may oxidize benzyl alcohol. The current density can achieve 340, 455 and 540 mA cm^−2^ at potential of 1.3, 1.4, and 1.5 V vs. RHE, respectively, based on the geometric surface area of the electrode. To the best of our knowledge, the current densities under these low potentials represent the advantageous performance in electrocatalytic AORs compared with other reported Au-based electrocatalysts (Supplementary Fig. 4 and Table 1) and also other noble/non-noble metal-based electrocatalysts (Fig. 2e and Supplementary Table 2)^27–32^. In addition, the Au/CoOOH begins to deactivate at potential (>1.5 V vs. RHE) higher than other samples, indicating a superior stability. We also tested the catalytic performance of other MOOH supported Au such as NiOOH and FeOOH, but only found Au/CoOOH showed superior current density (Supplementary Fig. 5).

### Benzyl alcohol electrooxidation performances

We then compared the catalytic performance of Au, Au/CoOOH and Au/NiFeOOH in benzyl alcohol oxidation at a constant potential (1.3 V *vs*. RHE and Supplementary Fig. 6) in 1 M KOH with 0.1 M benzyl alcohol. As shown in Fig. 2f, the conversion rate for benzyl alcohol oxidation over Au/CoOOH reaches 3.19 mmol cm^−2^ h^−1^ (corresponding to 968 C cm^−2^ h^−1^) at 1.3 V *vs*. RHE, which is 15~26-fold higher than that of other samples. All the samples exhibit high FE of >98% for benzyl alcohol oxidation, indicating no OER or H_2_ oxidation reaction (HOR) occurred at the anode. This is also rationalized by spin-polarized DFT calculations (Supplementary Note 1, and Supplementary Figs. 7 and 8). The kinetic curves of benzyl alcohol oxidation over Au/CoOOH reveal that it is first oxidized to benzaldehyde and then to benzoic acid (Supplementary Fig. 9a). Benzaldehyde was completely converted to benzoate with yield of 99% after 8 h, thus the products separation issue can be avoided. To collect benzoic acid from benzoate, we adopted acid neutralization and following filtration treatments. By doing these, benzoic acid was obtained with yield up to 92% and near 100 % purity (Supplementary Fig. 9b, c). Of note, acid neutralization of the carboxylate products might not be economically feasible, which is also considered an existing challenge for electrocatalysis taking place in alkaline electrolytes. We propose some possible solutions in Supplementary Note 2, expecting to address this issue. In terms of benzaldehyde, we found that the selectivity is sensitive to the pH of electrolyte, achieving high selectivity of benzaldehyde when the pH of electrolyte was decreased (Supplementary Fig. 10). From the catalyst structure point of view, the product selectivity can be further optimized by regulation of benzaldehyde adsorption strength via engineering the electronic and geometric structures of the catalyst (see Supplementary Note 3 for detailed deduction). Besides the anodic organic products, H_2_ production takes place at the counterpart Pt cathode. The space-time yield of H_2_ reaches 117.9 mL cm^−2^ h^−1^ when Au/CoOOH is used as the anode, which is significantly higher than Au (4.1 mL cm^−2^ h^−1^) and other Au supported samples (5.1–7.5 mL cm^−2^ h^−1^).

To rule out the possibility that the activity of Au/CoOOH is merely from CoOOH, we prepared CoOOH and the current density was far inferior than Au/CoOOH (Supplementary Fig. 11). We further poisoned Au sites on Au/CoOOH by using methionine regent or pre-oxidizing Au/CoOOH to form AuO_*x*_ (Supplementary Fig. 12). In both cases, we observed a significant decay of current density. The above results reveal that Au is indispensable for benzyl alcohol oxidation and CoOOH play important role to enhance the activity.

To compare the intrinsic activity over different samples in benzyl alcohol oxidation, we calculated the turnover frequency (TOF) at different potentials. As shown in Supplementary Figs. 13 and 14, the TOF of Au/CoOOH is only slightly higher than that of Au at the potential of 1.0 and 1.1 V *vs*. RHE, indicating the intrinsic activity contributed by Au in Au and Au/CoOOH is similar. The slightly high activity of Au/CoOOH may be due to the enhanced adsorption of benzyl alcohol on Au/CoOOH interface, as verified by Fourier transform infrared spectroscopy (FTIR) and supported by theoretical calculations shown latter. However, at 1.2 V *vs*. RHE, the TOF of Au/CoOOH is significantly higher than others. As the intrinsic activity of Au is similar as demonstrated at lower potential, the higher TOF of Au/CoOOH is more likely contributed by CoOOH. Considering that CoOOH can generate electrophilic OH* at this potential range, these results suggest that the OH* generated over CoOOH exhibits even higher activity than Au–OH species in benzyl alcohol oxidation (see Supplementary Note 4 for detailed discussion).

### Characterization of the optimum Au/CoOOH

**Fig. 3:**
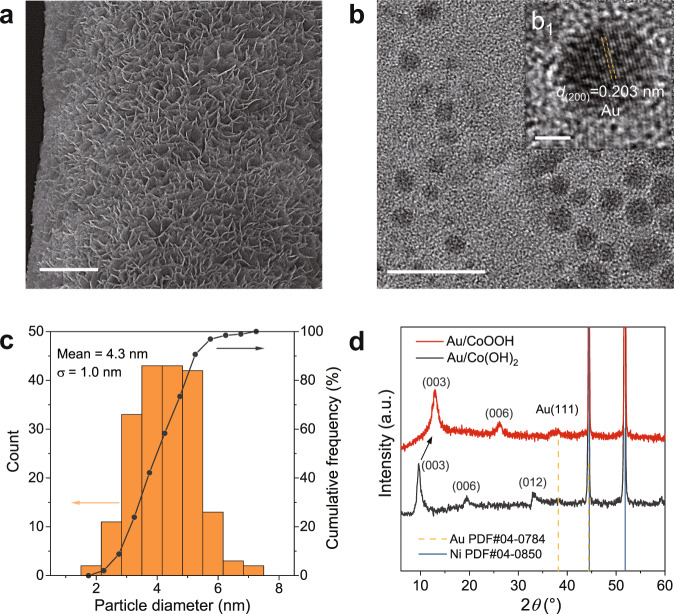
Structural characterization of the Au/CoOOH catalyst. **a** SEM image of the Au/CoOOH catalyst. Scale bar, 3 μm. **b** HRTEM images of a representative region. Inset displays an individual Au nanoparticle. Scale bars: 20 nm (**b**); 2 nm (**inset**). **c** Size distribution of the Au NPs. **d** XRD patterns of the Au/CoOOH catalyst and Au/Co(OH)_2_.

Scanning electron microscope (SEM) images (Fig. 3a) show that the CoOOH nanosheets (with an average thickness of ∼10 nm and diameter of 200–300 nm) are vertical on Ni foam with array structure. High-resolution transmission electron microscope (HRTEM) images reveal that Au NPs are well dispersed on CoOOH nanosheets (Fig. 3b and Supplementary Fig. 15) with average diameter of 4.3 nm (Fig. 3c). The HRTEM image of an individual Au nanoparticle displays a fringe distance of 0.203 nm (Fig. 3b; inset), closely matching the (200) plane of face-centered-cubic Au. X-ray diffraction (XRD) analysis of Au/CoOOH reveals a *γ*-CoOOH phase with typical (003) and (006) reflections at 12.9° and 26.2°, which is electrochemically transformed from its precursor, *α*-Co(OH)_2_^33,34^ (Fig. 3d and Supplementary Fig. 16). The reflection peak at 2*θ* = 38.2° for both Au/Co(OH)_2_ and Au/CoOOH can be attributed to Au (111), and the sharp peaks at 2*θ* = 44.4° and 51.8° are from Ni foam. Inductively coupled plasma-atomic emission spectrometry (ICP-AES) shows that the mass loading of Au on CoOOH is 11.3 wt% (Supplementary Table 3).

### Benzyl alcohol enrichment at Au/CoOOH interface

**Fig. 4:**
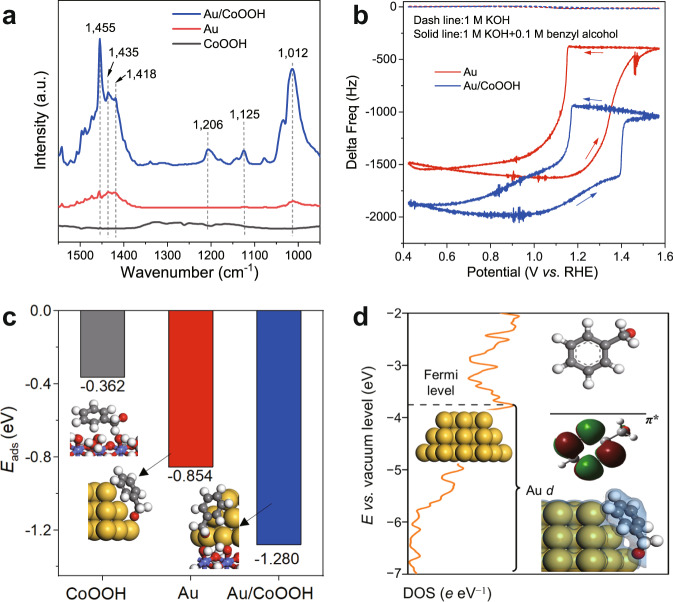
Mechanistic investigation of benzyl alcohol enrichment. **a** FTIR spectra of CoOOH, Au, and Au/CoOOH after benzyl alcohol adsorption and 15 min of He purging. **b** EQCM frequency response over Au and Au/CoOOH in 1 M KOH or 1 M KOH with 0.1 M benzyl alcohol. Scan rate: 50 mV s^−1^. **c** Adsorption energies of benzyl alcohol in the form of alkoxide on CoOOH, Au, and Au/CoOOH, respectively. The optimized geometries of Ph−CH_2_O^−^* for CoOOH, Au, and Au/CoOOH are also displayed. The color of each element is yellow for Au, blue for Co, red for O, white for H and gray for C, respectively. **d** Partial density of states for Au *d* orbital in Au/CoOOH, and the energy level of π* orbital (LUMO) for benzyl alkoxide. The isosurface of Ph−CH_2_O^−^* (* is Au/CoOOH) is also displayed.

It is reported that reactant adsorption with appropriate strength is also important for enhancing catalytic activity^35^. To study the adsorption behavior of benzyl alcohol over Au/CoOOH, in-situ FTIR spectroscopy was conducted. When benzyl alcohol was adsorbed on CoOOH, the peak positions are close to gaseous benzyl alcohol (Supplementary Fig. 17a)^36,37^, but it was easily removed by helium (He) purging within 15 min (Fig. 4a and Supplementary Fig. 17b), indicating benzyl alcohol is weakly adsorbed on CoOOH. For benzyl alcohol adsorption on Au and Au/CoOOH, the band intensity that corresponds to the skeletal vibration of benzene ring (peak at 1455 cm^−1^) decreases largely and widens towards lower wavenumber, together with the appearance of bands at around 1435 and 1418 cm^−1^ (Fig. 4c and Supplementary Fig. 17c, d). These variations may be attributed to the interaction between Au and benzene ring of benzyl alcohol that leads to the decrease of benzene symmetry, because breaking of symmetry was demonstrated to give rise to band shifting and splitting^38^. Moreover, the bands of *δ*(O−H) and *ν*(C−O) shift to lower wavenumbers and the intensity of *δ*(O−H) band is weakened due to the adsorption of benzyl alcohol^39,40^. Another band was observed at 1125 cm^−1^ that can be assigned to the stretching mode of C−O bond of alkoxide species adsorbed on Au^41^. After 15 min of He purging, the signal of adsorbed benzyl alcohol on Au/CoOOH remains intense, much higher than that on Au and CoOOH, indicating a stronger interaction exists between benzyl alcohol and Au/CoOOH.

The adsorption of benzyl alcohol over Au and Au/CoOOH was also examined by electrochemical quartz crystal microbalance (EQCM)^42^. As shown in Fig. 4b, both Au and Au/CoOOH show negligible frequency change in 1 M KOH. After 0.1 M benzyl alcohol was added, the Au sample exhibits a negative initial frequency at 0.4 V *vs*. RHE, revealing that benzyl alcohol can be spontaneously adsorbed on Au. The frequency increases in the positive scan due to the adsorption of more benzyl alcohol. However, the frequency starts to decay from ~1.2 V *vs*. RHE due to the formation of AuO_*x*_ that inhibits the adsorption of benzyl alcohol. By contrast, the initial frequency of Au/CoOOH after adding benzyl alcohol shows more negative shift than that of Au (blue curve in Fig. 4b), indicating that the adsorption of benzyl alcohol is promoted over Au/CoOOH, in agreement with the above CV results (Fig. 2b). With increasing potential, the frequency increases first and then decreases at ~1.1 V *vs*. RHE. This may be due to the formation of electrophilic OH* on CoOOH that accelerates the consumption of adsorbed benzyl alcohol on Au/CoOOH. The frequency finally decays at the potential of ~1.4 V *vs*. RHE that is higher than Au, demonstrating that Au deactivation is delayed over Au/CoOOH.

### Mechanistic studies for benzyl alcohol adsorption

The adsorption behavior of benzyl alcohol on Au/CoOOH was also studied by spin-polarized DFT calculations. Three models were constructed and denoted as CoOOH, Au and Au/CoOOH, representing the reaction sites of the (110) surface of *γ*-CoOOH, the (111) surface of Au, and Au (111)/*γ*-CoOOH (110) interface (Supplementary Figs. 18 and 19; the reason to choose these reaction sites is illustrated in the Methods section and Supplementary Note 5). Since the reaction takes place in an alkaline electrolyte, *i.e*., 1 M KOH, benzyl alcohol is in the form of alkoxide. Therefore, benzyl alkoxide was used as the reactant in the calculations. Thus the adsorption energies (*E*_ads_) of benzyl alcohol in the form of alkoxide on CoOOH, Au, and Au/CoOOH were calculated and displayed in Fig. 4c. The adsorption of benzyl alkoxide on Au/CoOOH (*E*_ads_ of –1.280 eV) is significantly stronger than that on CoOOH (*E*_ads_ of –0.362 eV) and on Au (*E*_ads_ of –0.854 eV). By analyzing the Hirshfeld charge, bond order and density of states, together with the Frontier orbital of benzyl alkoxide, it can be deduced that the strong adsorption of benzyl alcohol on Au/CoOOH is contributed by both the *σ* bond between Au 6 *s* orbital and the lone pair electrons of *α*–O in –CH_2_O^–^ group, and the *π* bond between the occupied Au 5*d* orbital and the unoccupied π* orbital of benzyl alkoxide^43^ (see Fig. 2d, Supplementary Fig. 20 and Supplementary Note 6). Moreover, according to the Hirshfeld charge analysis, there is electron transferring from Au to CoOOH, giving rise to Au atoms in Au/CoOOH interface being more electron-deficient than those in pure Au (Supplementary Fig. 20), matching with the X-ray photoelectron spectra (XPS) results (Supplementary Fig. 21). As a result, electron transfer from −CH_2_O^−^ to Au 6 *s* orbital is enhanced, which in turn strengthen the Au−O bond. Together with the FTIR and EQCM analysis, these results show that benzyl alcohol adsorption and thus enrichment is enhanced over Au/CoOOH at the interface, which may contribute to its higher activity than that of Au.

### Reaction mechanism

**Fig. 5:**
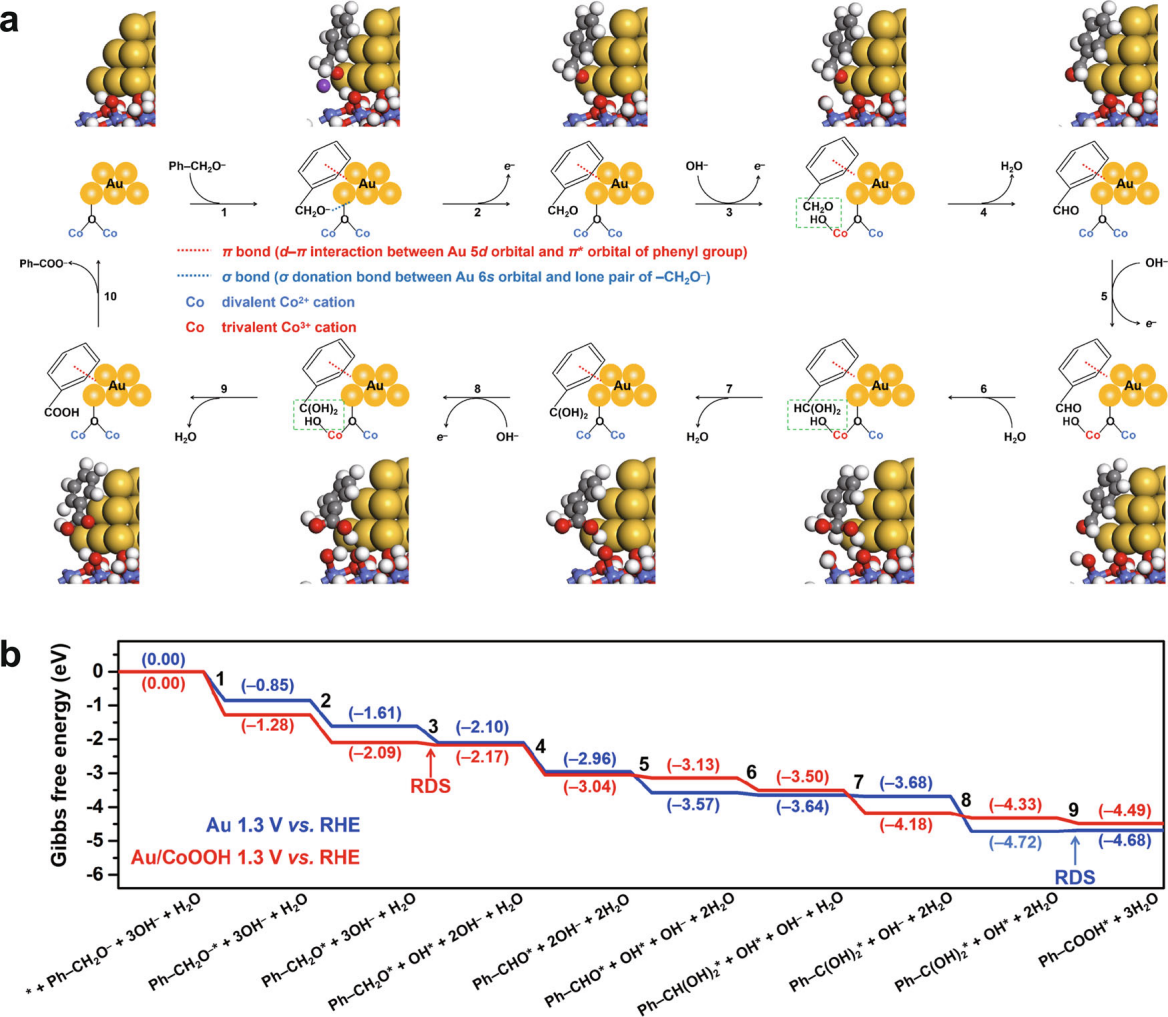
Proposed reaction mechanism of benzyl alcohol oxidation. **a**, Schematic illustrations of the reaction processes for benzyl alcohol oxidation on Au/CoOOH. The color of each element is yellow for Au, blue for Co, red for O, white for H, gray for C, and purple for K, respectively. The optimized geometries of reaction intermediates on Au/CoOOH are also displayed. **b**, Gibbs free energy diagrams for benzyl alcohol oxidation to benzoic acid on Au and Au/CoOOH under working condition. The numbers in the bracket are the Gibbs free energies in the unit of eV. The serial numbers for the elementary steps are also labeled in both a and b.

To further rationalize the superior activity of Au/CoOOH and illustrate the key role of OH* for benzyl alcohol oxidation, Gibbs free energy diagrams of benzyl alcohol oxidation to benzoic acid were calculated over Au and Au/CoOOH. As shown in Fig. 5a, the reaction begins with the adsorption of benzyl alkoxide on Au. When the potential is increased, the adsorbed benzyl alkoxide (Ph–CH_2_O^–^*) is converted to nucleophilic Ph–CH_2_O* with electron transferred to Au. Then electrophilic OH* is generated either on Au or CoOOH at certain potentials (0.856 V *vs*. RHE on Au and 1.216 V *vs*. RHE on CoOOH), which attacks on Ph–CH_2_O* to generate benzaldehyde (Ph–CHO*) along with proton-electron transfer process. Then Ph–CHO* is converted to Ph–CH(OH)_2_* by spontaneous hydration, which is followed by reaction with OH* to give Ph–C(OH)_2_* and finally Ph–COOH*. The Gibbs free energy diagrams of benzyl alcohol oxidation over Au and Au/CoOOH at 0 V *vs*. RHE are displayed in Supplementary Fig. 22. The rate-determining step (RDS) in benzyl alcohol oxidation are the generation of OH* for both Au and Au/CoOOH with the Gibbs free energy change (∆*G*) of 0.856 eV and 1.216 eV, respectively. Therefore, the OH* can be generated on Au at lower potential than that generated on CoOOH in Au/CoOOH, in accordance with the LSV and CV measurements (Fig. 2a, c). In order to reveal why the OH* generated on CoOOH in Au/CoOOH possesses stronger oxidation ability than that generated on Au, the Gibbs free energy diagrams at reaction potential (1.3 V *vs*. RHE) are displayed in Fig. 5b. The RDS over Au is switched to the nucleophilic attack of Ph–C(OH)_2_* on OH* (Δ*G* = 0.041 eV) since the Δ*G* for the generation of OH* is reduced to ‒0.444 eV with increased potential while the Δ*G* for the electrophilic oxidation of OH* keep invariant. For Au/CoOOH, the RDS is still the generation of OH* on CoOOH with a Δ*G* of –0.084 eV. As a result, the cooperative effect of Au/CoOOH interface enhancing benzyl alcohol adsorption and CoOOH generating OH* facilitate benzyl alcohol oxidation (see Supplementary Note 7 and Supplementary Figs. 23, 24 for detailed discussion of the reaction mechanism over Au and Au/CoOOH at different potentials).

To understand the higher activity of Au/CoOOH compared with other Au/MOOHs in benzyl alcohol oxidation, we compared the electronic interaction between Au and different MOOHs. The results suggest that there may be no significant difference in the electronic structure interaction between Au/CoOOH and other Au/MOOHs. In addition, there is comparable adsorption behavior between benzyl alcohol and different Au/MOOHs (Supplementary Fig. 25 and Supplementary Note 8). Thus we postulate that the enhanced current density of Au/CoOOH compared to other Au/MOOH samples (Fig. 2d and Supplementary Fig. 5) is more likely due to the catalytic activity of CoOOH that is capable of providing OH* to oxidize benzyl alcohol under reaction potential. To verify this postulation, we calculated the Δ*G* of oxygen species generation over different MOOHs. The results show that OH* can be generated on CoOOH at 1.248 V *vs*. RHE, while NiOOH, FeOOH, and NiFeOOH are more likely to generate O* as the reactive oxygen species, with the Δ*G* of O* generation larger than 0 eV at 1.3 V vs. RHE, namely, 0.099 eV for Au/NiOOH, 0.042 eV for Au/FeOOH, and 0.112 eV for Au/NiFeOOH, respectively (Supplementary Note 9, Supplementary Table 4, and Supplementary Figs. 26–32). This might be reason for the insignificant promotion effect for these three catalysts (Au/NiOOH, Au/FeOOH, Au/NiFeOOH) toward benzyl alcohol oxidation at reaction potential of 1.3 V vs. RHE. For pure CoOOH, although it can generate OH* at ~1.2 V *vs*. RHE, it exhibits weak adsorption ability for benzyl alcohol, resulting in poor benzyl alcohol oxidation activity with low oxidation onset potential of ~1.35 V *vs*. RHE and low current density.

### Substrate scoping

To demonstrate the generality of the AOR promotion effect over Au/CoOOH catalyst, benzyl alcohols with diverse substituents (−Cl, −F,−CH_3_,−CF_3_,−OCH_3_,−C(CH_3_)_3_) were examined. The electrooxidations were performed at 60 °C to guarantee alcohols full dissolution in 1 M KOH. The catalytic results show that Au/CoOOH exhibits 6 to 12-fold enhancement of current density (representing as charge transfer) compared with Au, together with higher production rates of oxidized products (entries 2–7, Table 1; and Supplementary Fig. 33) and comparably high FEs. Among these alcohol molecules, benzyl alcohol substituted with −C(CH_3_)_3_ shows inferior activity (entry 7, Table 1), which may be due to the steric hindrance that inhibits benzene ring adsorption on Au. Moreover, the Au/CoOOH catalyst can realize the selective oxidation of *α*-phenethyl alcohol to acetophenone (entry 8, Table 1; and Supplementary Fig. 34), which is insoluble in water and spontaneously floats on top of the electrolyte to be easily abstracted.

**Table 1:** Substrate scoping investigations.

Entry	Reactant	Au *vs*. Au/CoOOH				
		Charge transfer (C cm^−2^ h^−1^)	Production rate (mmol cm^−2^ h^−1^)		FE (%)	
			aldehyde/ketones	acid	aldehyde/ketones	acid
**1**		201.4/1463	0.21/0.91	0.41/3.27	21/12	77/86
**2**		99.4/1207.2	0.034/0.44	0.24/2.86	7/5	91/93
**3**		85.3/1020.1	0.12/0.86	0.12/1.81	56/16	27/69
**4**		178.1/1148.2	0.59/2.70	0.12/1.30	63/45	26/44
**5**		63.82/638.1	0.029/0.085	0.15/1.58	9/3	90/96
**6**		131.4/891.7	0.56/3.23	0.055/0.61	82/70	16/26
**7**		8.8/110.2	0.00/0.00	0.022/0.29	0/0	96/99
**8**		102.6/967.8	0.53/5.01	0/0	99/99	0/0
**9**		73.1/651.9	0.016/0.52	0.18/1.45	4/15	94/84
**10**		17.8/255.5	0.00/0.00	0.049/0.71	0/0	85/85^a^
**11**		36.4/322.9	0.00/0.00	0.10/0.90	0/0	98/98^b^
**12**		3.5/253.2	0.00/0.00	0.011/0.77	0/0	97/96^c^
**13**		86.7/406.2	0.00/0.00	0.33/1.38	0/0	92/91^d^

Comparison of the catalytic performance of electrooxidation of benzyl alcohol derivatives and alcohols with vicinal C=C, C=O and ‒OH groups over Au and Au/CoOOH catalysts.

Note. Entries 1–8 were reacted in 1 M KOH with 0.1 M reactant at 60 °C, among them, the products for Entries 1–7 are aldehyde and acid, Entries 8 is acetophenone only. Entries 9–13 were reacted in 1 M KOH with 0.3 M reactant at r.t.

^a^The total acid products of hydroxyacetone, including pyruvic acid (59%/58%), acetic acid (5%/6%) and formic acid (21%/21%).

^b^The total acid products of ethylene glycol, including glycolic acid (65%/68%) and formic acid (33%/30%).

^c^The total acid products of 1,2-propanediol, including lactic acid (26%/29%), acetic acid (26%/28%) and formic acid (45%/39%).

^d^The total acid products of glycerol, including glyceric acid (24%/20%), lactic acid (2.0%/11%), glycolic acid (35%/26%) and formic acid (31%/34%).

The above experimental and theoretical results suggest that benzene ring with *π* bond and *α*–C–OH are both important functional groups for the enhanced adsorption of alcohols on Au/CoOOH with promoted current density. We then investigated the alcohols with *α*–C=C (methallyl alcohol) and *α*–C=O group (hydroxyacetone), because these groups also contain *π* bond that resembles benzene ring. As expected, electrooxidation of these alcohols were also enhanced over Au/CoOOH compared with Au (entries 8 and 9, Table 1; and Supplementary Fig. 35). DFT calculations reveal that the adsorption energies of methallyl alcohol and hydroxyacetone on Au/CoOOH are –1.21 and –1.00 eV, respectively, which is assigned to the strong *σ*‒*π* bonds (see Supplementary Fig. 36 and Supplementary Note 10). Furthermore, we found that Au/CoOOH also shows enhanced current density for electrooxidation of polyols with vicinal hydroxyl groups, including ethylene glycol, 1,2-propanediol and glycerol (entries 10–12, Table 1; and Supplementary Fig. 37). It is worth noting that glycerol is an important biomass derivative produced in large amount annually in biodiesel production. Previous works have demonstrated that polyols were firstly electrooxidized to corresponding hydroxy aldehydes (or ketones) over Au and other noble-metal catalysts in alkaline media^16,17^. For example, Tremiliosi-Filho and co-workers demonstrated the formation of glyceraldehyde intermediate in the electrooxidation of glycerol over Au catalyst in an alkaline solution^18^. Thus, we postulate that the oxidation of -CH_2_−OH to -C=O may also take place under our reaction conditions (1 M KOH with 0.1 M polyols), with the formation of *α*-C=O group showing enhanced adsorption on Au/CoOOH via *π* bonding as demonstrated for hydroxyacetone (entry 9, Table 1).

We then examined three alcohols without *π* bond or *α*–C–OH, namely ethanol, 1,3-propanediol and *β*-phenylethanol. The current densities of these alcohols oxidation on Au/CoOOH, as well as Au and Au/NiFeOOH, were much lower than that of benzyl alcohol (Supplementary Figs. 38, 39, and Supplementary Note 11). We speculate that the superior current density for benzyl alcohol oxidation is not only related with its enhanced adsorption on Au/CoOOH interface, but also linked with the electronic structure of benzyl alcohol. Inspired by the molecular orbital theory, the energy levels of the highest occupied molecular orbital (HOMO) for benzyl alcohol, ethanol, 1,3–propanediol, and *β*–phenylethanol were calculated, which was –6.458 eV, –7.497 eV, –7.256 eV, and –6.701 eV, respectively. The HOMO of benzyl alcohol lies higher than the other three alcohols, revealing that the ionization energy of benzyl alcohol is smaller, and thus benzyl alcohol is easier to be oxidized by the electrophilic OH* on Au/CoOOH (Supplementary Note 12). The mechanism of ethanol oxidation to acetic acid on Au/CoOOH was also calculated (Supplementary Note 13, Supplementary Figs. 40 and 41). It is found that the RDS of ethanol oxidation on Au/CoOOH is 0.488 eV under reaction potential, which is larger than 0 eV, thus the oxidation of ethanol is slow under reaction potential. Moreover, the adsorption energy of ethanol on Au/CoOOH was calculated to be –0.51 eV, which is much weaker than that of benzyl alcohol (–1.28 eV). Both the strengthened adsorption and lowered reaction barrier contribute to the fast oxidation rate of benzyl alcohol on Au/CoOOH.

### Understanding of the decay and restoring of current density

**Fig. 6:**
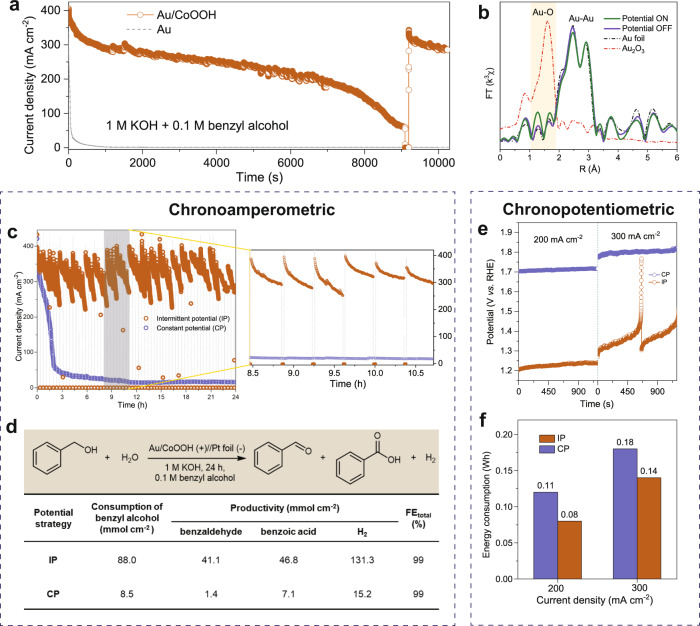
Current density revival of Au/CoOOH. **a** *I*-*t* curve of Au/CoOOH catalyst at 1.35 V *vs*. RHE in a 1 M KOH with 0.1 M benzyl alcohol at r.t. **b** Fourier-transform Au *L*_3_-edge EXAFS spectra of Au/CoOOH, Au foil and Au_2_O_3_. **c** Chronoamperometric measurements of Au/CoOOH catalyst using intermittent potential (IP) and constant potential (CP) strategies at 1.35 V *vs*. RHE in a 1 M KOH with 0.1 M benzyl alcohol at r.t. over 24 h (left), and enlarged measurement period in **a** (right). **d** The corresponding consumption of benzyl alcohol, productivities of benzaldehyde, benzoic acid and H_2_, as well as the FE of total anodic products. **e** Chronopotentiometric measurements of Au/CoOOH catalyst with IP and CP strategies at 200 and 300 mA cm^−2^ in a 1 M KOH with 0.1 M benzyl alcohol at r.t. **f** The corresponding electric energy consumption.

The stability of Au/CoOOH catalyst for benzyl alcohol oxidation was also studied. As shown in Fig. 6a, the current-time (*I-t*) curves show that the current density of the pure Au decreases rapidly to <5 mA cm^−2^ after 900 s at 1.35 V *vs*. RHE in a 1 M KOH with 0.1 M benzyl alcohol at r.t. This is due to the oxidation of Au to form AuO_*x*_ (Supplementary Fig. 42). In apparent contrast, the current density maintains above 200 mA cm^−2^ after 7000 s over Au/CoOOH due to the oxidation of Au is delayed, which is confirmed by operando extended X-ray absorption fine structure spectroscopy (operando EXAFS) (Supplementary Fig. 43). However, the current density decays eventually after a long-term reaction over the Au/CoOOH (Fig. 6a), suggesting that Au would still get oxidized. This is revealed by the Fourier-transform Au *L*_3_-edge EXAFS spectrum, showing the appearance of Au−O bond attributed to AuO_*x*_ formation over Au/CoOOH (Fig. 6b).

The origin of the stability of Au/CoOOH than Au is likely associated with the stronger adsorption of benzyl alcohol on Au/CoOOH interface compared with that on Au, which is capable of reducing the in-situ formed Au oxide and thus dynamically retarding the Au deactivation process. Indeed, alcohols were used as reducing agents in many reaction systems.^44,45^ To verify the reduction ability of benzyl alcohol, we pre-oxidized Au and studied its reduction kinetic in the absence or in the presence of benzyl alcohol at open circuit. The results show that Au oxide cannot be reduced in 1 M KOH electrolyte but was readily reduced in the presence of benzyl alcohol (Supplementary Fig. 44). Based on the understanding of the reduction ability of benzyl alcohol, we cut off the potential for approximately 100 s at open circuit (denoted as potential OFF) after the Au got deactivated, and then applied anodic potential (denoted as potential ON). We found that the initial high current density can be readily restored (Fig. 6a). The reduction of Au oxide was confirmed by Fourier-transform Au *L*_3_-edge EXAFS spectra (Fig. 6b), in which the Au−O signal was significantly decreased at potential OFF. Notably, operando EXAFS measurement shows that the Au oxidation /reduction processes are reversible when more potential ON/OFF were applied (Supplementary Fig. 45), suggesting that it would be feasible to regain the high current density by exerting potential OFF for a short time at the interval of a long-term electrooxidation when the current density decreases to a certain level.

### Intermittent potential strategy

In light of the above findings, we developed an intermittent potential (IP) strategy to maintain the original high current density in a long-term electrooxidation, through alternatively switching between potential ON and potential OFF (see Method for details). By repeating the above procedures, the current density could be maintained at a high level (250–400 mA cm^−2^) over 24 h (Fig. 6c). For a longer time operation, the current density can be maintained at 100–170 mA cm^−2^ at 1.2 V vs RHE over 108 h (Supplementary Fig. 46). In contrast, the current density of Au/CoOOH decays from 400 to 60 mA cm^−2^ within 2 h using a traditional constant potential (CP) strategy. As a result, the Au/CoOOH with IP strategy exhibits larger benzyl alcohol consumption, higher productivities of benzaldehyde, benzoic acid and H_2_ compared with CP strategy, with high FE of total anodic products (Fig. 6d). The Au/CoOOH maintains its structure after the long-term reaction (Supplementary Fig. 47), which demonstrates its high stability. We further conducted chronopotentiometric measurements to compare the electric energy consumption of IP and CP strategy. As shown in Fig. 6e, the Au/CoOOH with the IP strategy requires much lower potential than that of CP strategy to reach the same current. The calculated electric energy consumption was 0.08 and 0.14 Wh for IP at constant current density of 200 and 300 mA cm^−2^, respectively, much lower than that of CP with electric energy consumption of 0.12 and 0.18 Wh at 200 and 300 mA cm^−2^, respectively (Fig. 6f and Supplementary Table 5). Thus, the IP strategy exhibited 33–43% energy saving than CP strategy. For the real application of this IP strategy, it is critical to slow down the deactivation process of Au catalyst, and we found that lowering the reaction potential is an effective way. As shown in Supplementary Fig. 48, at the potential of 1.4 V vs. RHE (curve in black), the current density of Au/CoOOH decayed from 400 to 100 mA cm^−2^ after ~3000 s. When we reduced the potential, the deactivation time was extended to 8000 s at 1.35 V vs. RHE (curve in red) and longer than 10000 s at 1.3 V vs. RHE (curve in blue). We believe that the deactivation of Au can be further delayed through designing Au catalyst that exhibits higher activity at low potential, thus avoiding applying high potential. The design of more active Au catalysts is undergoing in our laboratory. In addition, inspired by the recent work^46^, we set up a symmetric single compartment (SSC) system to save energy and time, in which both electrodes are identical and perform the same set of reactions in one compartment. As schematic illustrated in Supplementary Fig. 49, two identical Au/CoOOH electrodes alternatively proceed alcohol oxidation (as anode) and H_2_ production (as cathode). By using this system, once the Au/CoOOH catalyst is used for H_2_ production as the cathode, Au can be rapidly reduced and reactivated. This may effectively avoid the waste of time and energy by IP strategy. However, as the HER activity of our Au/CoOOH catalyst is not high, the current SSC strategy still has the problem of high energy consumption. We will attempt to support component such as Pt that is active for HER on Au/CoOOH, expecting to design a bifunctional catalyst with benzyl alcohol oxidation and HER activities.

**Fig. 7:**
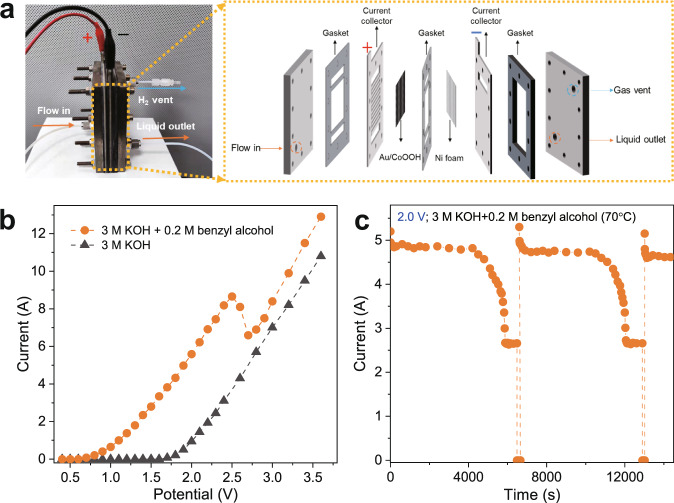
Flow electrolyzer. **a** Photograph and schematic illustration of the membrane-free flow electrolyzer. **b** The LSV curves of Au/CoOOH in 3 M KOH with or without 0.2 M benzyl alcohol at 70 °C in the membrane-free flow electrolyzer. **c** *I*-*t* curve of Au/CoOOH catalyst using IP strategy at 2 V in a 3 M KOH with 0.2 M benzyl alcohol at 70 °C in the membrane-free flow electrolyzer.

To evaluate the catalyst in a more practical scenario, we carried out two-electrode tests in a homemade membrane-free flow electrolyzer using Au/CoOOH catalyst as anode and Ni foam as cathode with working area of 30 cm^2^ (Fig. 7a and Supplementary Fig. 50). The electrochemical tests were performed at industrially-relevant conditions (*i.e*., 70 °C and 3 M KOH). The LSV curves in Fig. 7b show that the onset potential of benzyl alcohol oxidation is 0.7 V, much lower than that of OER. The current of benzyl alcohol oxidation increases until a temporary decay at ~2.5 V due to the formation of AuO_*x*_, with the following increase from ~2.7 V, which is ascribed to benzyl alcohol oxidation by CoOOH. The electrolyzer outputted an absolute current of 4.8 A at 2.0 V for benzyl alcohol oxidation using IP strategy (Fig. 7c, see Method for details), achieving benzyl alcohol conversion rate of 39.8 mmol h^−1^ and benzoic acid productivity of 36.6 mmol h^−1^. The space-time yield of H_2_ reaches ~1.9 L h^−1^ with purity of 99.99%. Together with the good stability of the structure of Au/CoOOH catalyst after electrochemical test (Supplementary Fig. 51), these results suggest the potential of Au/CoOOH catalyst to work under industrially-relevant conditions.

## Discussion

In summary, we achieved the electrooxidation of benzyl alcohol coupled with H_2_ production at high current density by using a cooperative Au/CoOOH catalyst. Experimental and spin-polarized DFT calculations reveal that the benzyl alkoxide is enriched at Au/CoOOH interface and oxidized by the electrophilic OH* generated on CoOOH with low reaction barrier, resulting in the fast reaction rate of benzyl alcohol oxidation to benzoic acid on Au/CoOOH. The substrate can be applied to a wide range of alcohols with *α*‒*π* bond including *α*‒phenyl, C = C and C = O groups. Based on the finding that Au can be reversibly oxidized/reduced at anodic potential/open circuit, we design an IP strategy, which achieves long-term alcohol electrooxidation at high current density with promoted productivities and reduced energy consumption. We believe the IP strategy may find its application in the natural intermittent energies (such as wind power, tidal power, waves), which provide a pause time for catalyst reactivation. This work offers a promising opportunity to enhance the current density of the catalyst by engineering interfacial sites for reactants enrichment toward electrosynthesis of valuable chemicals coupled H_2_ production.

## Methods

### General information

Except noted, all chemicals were purchased and used without further purification.

### Catalyst preparation

The Au NPs supported on CoOOH nanosheet catalyst (Au/CoOOH) was prepared via a two-step electrochemical method, in which Co(OH)_2_ nanosheets was initially grown on nickel (Ni) foam, then Au NPs were electrodeposited onto Co(OH)_2_. Subsequently, the as-synthesized Au/Co(OH)_2_ was electro-oxidized in 1 M KOH solution to enable the structural transformation to Au/CoOOH.

#### Preparation of Co(OH)_2_ nanosheets

The nickel foam (10 × 20 × 1.5 mm) was used as matrix for growing Co(OH)_2_ nanosheets array. Initially, the nickel foam (10 × 20 × 1.5 mm) was sequentially washed with dilute HCl (1 M), ethanol, and deionized water (each for 10 min) to remove surficial oxides and contaminants. The electrosynthesis of Co(OH)_2_ nanosheet array was carried out in a three-electrodes setup, using saturated calomel electrode (SCE) and Pt foil as reference and counter electrodes respectively. The growth of Co(OH)_2_ nanosheets was performed at constant potential (−1.0 V *vs*. SCE) in aqueous Co(NO_3_)_2_ (0.15 M) electrolyte for 300 seconds. The potentiostatic deposition was carried out at a potential of −1.0 V *vs*. SCE. The resulting Co(OH)_2_ nanosheet array was withdrawn and rinsed thoroughly with ethanol and distilled water^23^.

#### Preparation of Au/Co(OH)_2_

The electrochemical deposition of Au NPs onto Co(OH)_2_ was carried out in a three-electrode configuration as above described. Specifically, Au NPs were deposited on Co(OH)_2_ by stepping the potential to −0.6 V *vs*. SCE for 10 s, followed by stepping back to −0.2 V *vs*. SCE for 10 s for three cycles, using aqueous electrolyte with 0.1 M NaCl and 5 mM HAuCl_4_^47^. The pure Au catalyst was prepared via the similar electrodeposition method by directly using Ni foam as the working electrode.

#### Preparation of Au/CoOOH

The Au/CoOOH was obtained from the as-prepared Au/Co(OH)_2_ via a simple cyclic voltammetry (CV) method in a three-electrode configuration, using Ag/AgCl (with saturated KCl) and Pt foil as the reference and counter electrodes respectively. The electrochemical oxidization process was performed at a scan rate of 100 mV s^−1^ from 0 V to 0.8 V *vs*. Ag/AgCl for approximately 20 cycles in 1 M KOH solution. The pure CoOOH catalyst was prepared via the similar electrochemical oxidization method by using as-obtained Co(OH)_2_ as the working electrode.

### Characterizations

X-ray diffraction patterns were collected on a Shimadzu XRD-6000 diffractometer using a Cu Kα source, with a scan range of 3−70° and scan step of 5° min^−1^. X-ray photoelectron spectra (XPS) were performed on a Thermo VG ESCALAB 250 X-ray photoelectron spectrometer at a pressure of about 2 × 10^−9^ Pa using Al Kα X-rays as the excitation source. Scanning electrode microscope (SEM) imaging was performed using a Zeiss SUPRA 55 Field Emission SEM with an accelerating voltage of 20 kV. Transmission electron microscopy (TEM) images were recorded with JEOL JEM-2010 high resolution (HR-) TEM with an accelerating voltage of 200 kV, combined with energy dispersive X-ray spectroscopy (EDX) for the determination of metal composition. Metal contents in catalysts were determined by ICP-AES on a Thermo ICAP6300 Radial. The operando Au and Co XAFS measurements was performed at the beamline 1W1B of the Beijing Synchrotron Radiation Facility (BSRF), Institute of High Energy Physics (IHEP), Chinese Academy of Sciences (CAS). Extended X-ray absorption fine structure spectra (EXAFS) were recorded at ambient temperature in transmission mode. The typical energy of the storage ring was 2.5 GeV with a maximum current of 250 mA; the Si (111) double crystal monochromator was used. Fourier transform of the EXAFS spectra were carried out in a *K*-range from 3.0 to 12.8 Å^−1^. The in-situ FTIR spectra of benzyl alcohol were carried out in a Bruker Equinox 55 spectrometer, between 4000 and 400 cm^−1^ with a resolution of 4 cm^−1^ after 600 scans per spectrum. About 30 mg of the sample was pressed into a wafer with a diameter of 13 mm, which was then installed in an in-situ IR reactor with CaF_2_ windows. The sample was pre-processing by He gas at r.t. for 1 h and then benzyl alcohol was flowed into the cell for 30 min, then physically adsorbed benzyl alcohol was removed by flowing He gas for 30 min. The FTIR spectra were in-situ collected during the He purging process^37^.

### Electrochemical measurement

All electrochemical measurements for alcohols oxidation were performed in 1 M KOH electrolyte at r.t. or 60 °C on an electrochemical workstation (CHI 760E, CH Instruments, Inc.). The electrochemical tests were performed in a three-electrode system in a membrane-free glass beaker, using Ag/AgCl electrode (with saturated KCl) and Pt foil as reference and counter electrode, respectively (Supplementary Fig. 52). Linear scan voltammetry (LSV) curves of catalysts were acquired from −0.2 V to 0.6 V vs. Ag/AgCl at a scan rate of 10 mV s^−1^. All potentials measured against Ag/AgCl were converted to the reversible hydrogen electrode (RHE) scale using: *Evs*. RHE = *Evs*. Ag/AgCl+ 0.197 +0.059ph. The space-time yield of H_2_ in Fig. 2d was obtained by gas-collecting method of drainage water.

The FEs of all the products were calculated based on their corresponding electron transfer per molecule oxidation using the following equations.

I $${{{{{\rm{Faradaic}}}}}}\,{{{{{\rm{efficiency}}}}}}=\frac{{e}_{{{{{{\rm{products}}}}}}}\times {n}_{{{{{{\rm{products}}}}}}}\times N}{{{{{{\rm{Q}}}}}}/n}\times 100 \%$$

where *e*_products_ is the number of electrons required to oxidize alcohol/H_2_O molecule to products, including ketones/aldehydes without C‒C cleavage (*e* = 2), acid without C‒C cleavage (*e* = 4). For C‒C cleavage products, 2 electrons are added to the *e*_products_ to break each C‒C in addition to the electrons to form ketones/aldehydes/acid. *n*_products_ is the productivity of products, *N* is Avogadro’s constant (*N* = 6.02 × 10^23^), *Q* is the quantity of electric charge, and *n* is the elementary charge (*e* = 1.602 × 10^−19^ C).

The electric energy consumption using IP and CP strategies were calculated as follows: the chronopotentiometric measurements were performed to evaluate the electric energy consumption. The value of electric energy consumption was determined using the following equation:^16^

II $${W}_{E}=I\times U\times t$$

where *I* stands for the current density (for example, 200 and 300 mA cm^−2^ in our calculations), *U* is the reaction potential and *t* is the reaction time (0.33 h in our calculations).

For long-term electrochemical measurement by IP, the reaction was initially conducted at 1.35 V vs. RHE in 1 M KOH with 0.1 M benzyl alcohol, and a high current density (~400 mA cm^−2^) was observed as expected. When the current density decays to <250 mA cm^−2^, we cut off the potential for about 100 s and then switched the potential back to 1.35 V *vs*. RHE, giving rise to the recovery of the initial high current density. For IP strategy in membrane-free flow electrolyzer, we cut off the voltage for about 120 s when the current decays to <2.6 A, and then switched the voltage back to 2 V to recover the initial high current.

The productivity of H_2_ in long-term electrochemical measurement by IP and CP strategies (in Fig. 6b) was calculated based on the charge transfer of benzyl alcohol oxidation. The aromatic alcohols, aldehydes, and acids were quantified by high performance liquid chromatography (HPLC; Angilent 1200 Infinity Series) equipped with C18 column (Cosmosil C18-MS-II) using MeCN/H_2_O/H_3_PO_4_ (40/60/0.05) as mobile phase and detected by UV detector at 220 nm. Aliphatic alcohols, aldehydes, and acids were quantified by HPLC equipped with organic acid column (Coregel 87H3) using 5 mM aqueous H_2_SO_4_ as mobile phase and detected by UV detector (210 nm) and refractive index detector.

Electrochemical quartz crystal microbalance (EQCM) measurements were performed as follows. The CoOOH, Au, and Au/CoOOH were electrodeposited on the conductive quartz crystal substrates using the same electrosynthesis method as above with the geometrical area of 0.5 cm^2^, which were used as working electrode. Pt wire and Ag/AgCl electrode were used as counter and reference electrodes respectively. The adsorption capacity of benzyl alcohol was obtained by comparing the mass changes of EQCM electrodes in 1 M KOH and 1 M KOH with 0.1 M benzyl alcohol. The change in mass per unit area (Δ*m*) was calculated from the changes in resonance frequency (Δ*f*), using the Sauerbrey equation: Δ*f* = ‒2 *f*_*0*_^*2*^Δm/[*A* (*µρ*)^0.5^], where *f*_*0*_ is the resonant frequency of the quartz resonator, *A* is the area of the EQCM electrode, *µ* is the shear modulus of the quartz and *ρ* is density of the quartz^42^.

### Computational details

#### Model construction

In order to represent the three possible reaction sites in the cooperative electrocatalyst Au/CoOOH, *i.e*., CoOOH surface, Au surface, and Au/CoOOH interface, three models were constructed and denoted as CoOOH, Au, and Au/CoOOH, respectively. Moreover, the models of Au/NiOOH, Au/FeOOH, and Au/NiFeOOH were also constructed to represent the control samples used in this work. The detailed information of model construction can be found in Supplementary Note 5 and 7 in the Supplementary Information.

#### Computational methods

The spin-polarized DFT calculations at the generalized gradient approximation (GGA) Perdew-Burke-Ernzerhof (PBE) level^48^ using a plane wave implementation were performed with the Cambridge Sequential Total Energy Package (CASTEP)^49^. The ionic cores of Au, Co, O and C were described by the ultrasoft pseudopotentials to decrease the number of plane waves required in the expansion of the Kohn-Sham orbitals^50^. The cut-off energy of the plane wave was set as 500 eV. To validate the convergence of the basis set size, the *E*_ads_ of Ph−CH_2_O^−^ on Au/CoOOH was calculated with a 600 eV cut-off. Adsorption energy varied by less than 0.020 eV, supporting the choice of 500 eV cut-off in this work. The Broyden-Fletcher-Goldfarb-Shanno (BFGS) algorithm was used to search the potential energy surface in geometry optimization^51^. The *k*-point meshes were set as 3 × 3 × 1 for all models in this work. To validate the convergence of *k*-points meshes, the *E*_ads_ of Ph−CH_2_O^−^ on Au/CoOOH was calculated with a 5 × 5 × 1 *k*-point meshes. The *E*_ads_ varied by less than 0.015 eV, indicating that the 3 × 3 × 1 *k*-point meshes has converged. During the geometry optimization, three convergence criteria were used as follows: (1) energy tolerance of 1.0 × 10^−6^ eV per atom, (2) force tolerance of 1.0 × 10^−2^ eV/Å, (3) displacement tolerance of 1.0 × 10^−3^ Å.

The *E*_ads_ of benzyl alcohol in the form of alkoxide was calculated with eq. (1):

1 $${E}_{{{{{{\rm{ads}}}}}}}={E}_{{{{{{{\rm{Ph}}}}}}-{{{{{\rm{CH}}}}}}}_{2}{{{{{{\rm{O}}}}}}}^{-}\ast }-{E}_{{{{{{{\rm{Ph}}}}}}-{{{{{\rm{CH}}}}}}}_{2}{{{{{{\rm{O}}}}}}}^{-}}-{E}_{\ast }$$

where *E*_ads_, ${E}_{{{{{{{\rm{Ph}}}}}}-{{{{{\rm{CH}}}}}}}_{2}{{{{{{\rm{O}}}}}}}^{-}\ast }$, E_*_, and ${E}_{{{{{{{\rm{Ph}}}}}}-{{{{{\rm{CH}}}}}}}_{2}{{{{{{\rm{O}}}}}}}^{-}}$ represent the adsorption energy of benzyl alcohol in the form of alkoxide, energy of Ph-CH_2_O^−^*, energy of *, and energy of benzyl alkoxide, respectively. The *E*_ads_ of Ph−CH_2_CH_2_O^−^, CH_2_=C(CH_3_)CH_2_O^−^ and CH_3_COCH_2_O^−^ were calculated in the similar way as eqs (2–4):

2 $${E}_{{{{{{\rm{ads}}}}}}}={E}_{{{{{{{\rm{Ph}}}}}}-{{{{{\rm{CH}}}}}}}_{2}{{{{{{\rm{CH}}}}}}}_{2}{{{{{{\rm{O}}}}}}}^{-}\ast }-{E}_{{{{{{{\rm{Ph}}}}}}-{{{{{\rm{CH}}}}}}}_{2}{{{{{{\rm{CH}}}}}}}_{2}{{{{{{\rm{O}}}}}}}^{-}}-{E}_{\ast }$$

3 $${E}_{{{{{{\rm{ads}}}}}}}={E}_{{{{{{{\rm{CH}}}}}}}_{2}{{{{{{\rm{C}}}}}}({{{{{\rm{CH}}}}}}_{3})}{{{{{{\rm{CH}}}}}}}_{2}{{{{{{\rm{O}}}}}}}^{-}\ast }-{E}_{{{{{{{\rm{CH}}}}}}}_{2}{{{{{{\rm{C}}}}}}({{{{{\rm{CH}}}}}}_{3})}{{{{{{\rm{CH}}}}}}}_{2}{{{{{{\rm{O}}}}}}}^{-}}-{E}_{\ast }$$

4 $${E}_{{{{{{\rm{ads}}}}}}}={E}_{{{{{{{\rm{CH}}}}}}}_{3}{{{{{{\rm{COCH}}}}}}}_{2}{{{{{{\rm{O}}}}}}}^{-}\ast }-{E}_{{{{{{{\rm{CH}}}}}}}_{3}{{{{{{\rm{COCH}}}}}}}_{2}{{{{{{\rm{O}}}}}}}^{-}}-{E}_{\ast }$$

The oxidation of benzyl alcohol to benzoic acid happens in nine consecutive steps:

A $$\ast +{{{{{\rm{Ph}}}}}}-{{{{{{\rm{CH}}}}}}}_{2}{{{{{{\rm{O}}}}}}}^{-}+3{{{{{{\rm{OH}}}}}}}^{-}+{{{{{{\rm{H}}}}}}}_{2}{{{{{\rm{O}}}}}}\to {{{{{\rm{Ph}}}}}}-{{{{{{\rm{CH}}}}}}}_{2}{{{{{{\rm{O}}}}}}}^{-}\ast +3{{{{{{\rm{OH}}}}}}}^{-}+{{{{{{\rm{H}}}}}}}_{2}{{{{{\rm{O}}}}}}$$

B $${{{{{\rm{Ph}}}}}}-{{{{{{\rm{CH}}}}}}}_{2}{{{{{\rm{O}}}}}}^-\ast +{{{{{{\rm{3OH}}}}}}}^{-}+{{{{{{\rm{H}}}}}}}_{2}{{{{{\rm{O}}}}}}\to {{{{{\rm{Ph}}}}}}-{{{{{{\rm{CH}}}}}}}_{2}{{{{{\rm{O}}}}}}\ast +{{{{{{\rm{3OH}}}}}}}^{-}+{{{{{{\rm{H}}}}}}}_{2}{{{{{\rm{O}}}}}}+{{e}}^{-}$$

C $${{{{{\rm{Ph}}}}}}-{{{{{{\rm{CH}}}}}}}_{2}{{{{{\rm{O}}}}}}\ast +3{{{{{{\rm{OH}}}}}}}^{-}+{{{{{{\rm{H}}}}}}}_{2}{{{{{\rm{O}}}}}}\to {{{{{\rm{Ph}}}}}}-{{{{{{\rm{CH}}}}}}}_{2}{{{{{\rm{O}}}}}}\ast +{{{{{\rm{OH}}}}}}\ast +2{{{{{{\rm{OH}}}}}}}^{-}+{{{{{{\rm{H}}}}}}}_{2}O+{e}^{-}$$

D $${{{{{\rm{Ph}}}}}}-{{{{{{\rm{CH}}}}}}}_{2}{{{{{\rm{O}}}}}}\ast +{{{{{\rm{OH}}}}}}\ast +2{{{{{{\rm{OH}}}}}}}^{-}+{{{{{{\rm{H}}}}}}}_{2}{{{{{\rm{O}}}}}}\to {{{{{\rm{Ph}}}}}}-{{{{{\rm{CHO}}}}}}\ast +2{{{{{{\rm{OH}}}}}}}^{-}+2{{{{{{\rm{H}}}}}}}_{2}{{{{{\rm{O}}}}}}$$

E $${{{{{\rm{Ph}}}}}}-{{{{{\rm{CHO}}}}}}\ast +2{{{{{{\rm{OH}}}}}}}^{-}+2{{{{{{\rm{H}}}}}}}_{2}{{{{{\rm{O}}}}}}\to {{{{{\rm{Ph}}}}}}-{{{{{\rm{CH}}}}}}{({{{{{\rm{OH}}}}}})}_{2}\ast +2{{{{{{\rm{OH}}}}}}}^{-}+{{{{{{\rm{H}}}}}}}_{2}{{{{{\rm{O}}}}}}$$

F $${{{{{\rm{Ph}}}}}}	-{{{{{\rm{CH}}}}}}{({{{{{\rm{OH}}}}}})}_{2}\ast +2{{{{{{\rm{OH}}}}}}}^{-}+{{{{{{\rm{H}}}}}}}_{2}{{{{{\rm{O}}}}}}\to {{{{{\rm{Ph}}}}}}-{{{{{\rm{CH}}}}}}{({{{{{\rm{OH}}}}}})}_{2}\ast +{{{{{\rm{OH}}}}}}\ast \\ 	+{{{{{{\rm{OH}}}}}}}^{-}+{{{{{{\rm{H}}}}}}}_{2}{{{{{\rm{O}}}}}}+{{e}}^{-}$$

G $${{{{{\rm{Ph}}}}}}-{{{{{\rm{CH}}}}}}{({{{{{\rm{OH}}}}}})}_{2}\ast +{{{{{\rm{OH}}}}}}\ast +{{{{{{\rm{OH}}}}}}}^{-}+{{{{{{\rm{H}}}}}}}_{2}{{{{{\rm{O}}}}}}\to {{{{{\rm{Ph}}}}}}-{{{{{\rm{C}}}}}}{({{{{{\rm{OH}}}}}})}_{2}\ast +{{{{{{\rm{OH}}}}}}}^{-}+2{{{{{{\rm{H}}}}}}}_{2}{{{{{\rm{O}}}}}}$$

H $${{{{{\rm{Ph}}}}}}-{{{{{\rm{C}}}}}}{({{{{{\rm{OH}}}}}})}_{2}\ast +{{{{{{\rm{OH}}}}}}}^{-}+2{{{{{{\rm{H}}}}}}}_{2}{{{{{\rm{O}}}}}}\to {{{{{\rm{Ph}}}}}}-{{{{{\rm{C}}}}}}{({{{{{\rm{OH}}}}}})}_{2}\ast +{{{{{\rm{OH}}}}}}\ast +2{{{{{{\rm{H}}}}}}}_{2}{{{{{\rm{O}}}}}}+{{e}}^{-}$$

I $${{{{{\rm{Ph}}}}}}-{{{{{\rm{C}}}}}}{({{{{{\rm{OH}}}}}})}_{2}\ast +{{{{{\rm{OH}}}}}}\ast +2{{{{{{\rm{H}}}}}}}_{2}{{{{{\rm{O}}}}}}\to {{{{{\rm{Ph}}}}}}-{{{{{\rm{COOH}}}}}}\ast +3{{{{{{\rm{H}}}}}}}_{2}{{{{{\rm{O}}}}}}$$

Among them, the steps A is the adsorption of benzyl alcohol and step E is the spontaneous hydration of benzaldehyde. The steps C, F, and H are the generation of electrophilic oxygen species OH* on the reaction site. The steps D, G, and I are the nucleophilic attack between the substrate and electrophilic OH*. The Δ*G* of each elementary step was calculated by minus the Gibbs free energy of reactant from that of the product. The Gibbs free energy of OH^−^ and e^−^ was calculated with the computational standard hydrogen electrode approximation (SHE, 0.5 H_2_ → H^+^ + *e*^−^, pH = 0, *p* = 1 atm, *T* = 298.15 K)^52^. The effect of pH and electric potential on Δ*G* were treated with terms “−*kT*ln10·pH” and “*eU*”, respectively. Therefore, Δ*G* of step C was calculated with eq. 5:

5 $$\varDelta {G}_{{{{{{\rm{C}}}}}}}={G}_{{{{{{{\rm{Ph}}}}}}-{{{{{\rm{CH}}}}}}}_{2}{{{{{\rm{O}}}}}}\ast +{{{{{\rm{OH}}}}}}\ast }-{G}_{{{{{{{\rm{Ph}}}}}}-{{{{{\rm{CH}}}}}}}_{2}{{{{{\rm{O}}}}}}\ast }-{G}_{{{{{{{\rm{H}}}}}}}_{2}{{{{{\rm{O}}}}}}}-0.5{G}_{{{{{{{\rm{H}}}}}}}_{2}}-eU-kT\,{{{{\mathrm{ln}}}}}\,10\cdot {{{{{\rm{pH}}}}}}$$

The Δ*G* of the other elementary steps were calculated in the similar way. The Gibbs free energies of models were obtained by calculating the phonon density of states, as shown in eq. 6:

6 $$G=E+ZPE+kT\int F(\omega ){{{{\mathrm{ln}}}}}\,\left[1-\exp \left(-\frac{\hslash \omega }{kT}\right)\right]d\omega$$

where *E* is the total energy, *ZPE* is the zero-point energy, the third term is the correction of Gibbs free energy *via* thermodynamics analysis. The explicit impact of the electrochemical potential was assessed for the RDS of benzyl alcohol oxidation to benzoic acid on Au/CoOOH. This impact is simulated by calculating the energy as a function of the chemical potential. The chemical potential of the system is tuned by charging the slab using an approach called “grand canonical DFT” and is associated with a Poisson–Boltzmann electrolyte for the charge compensation. This scheme is accomplished by VASPsol^53^ and has been widely used in previous literature^54,55^. The energy correction due to the explicit inclusion of the electrochemical potential treated with Poisson–Boltzmann scheme is negligible (<0.05 eV) for the RDS, generation of OH* on Au/CoOOH, in accordance with previous literature^56^. Therefore, the computational standard hydrogen electrode approximation was used in this work. More accurate grand canonical DFT method would be considered in our future work.

## Supplementary information

Supplementary Information

## Data Availability

The data supporting the findings of this study are available within the article and its Supplementary Information. The source data generated in this study and the optimized geometries of catalysts and reaction intermediates are available in the figshare repository (https://figshare.com/s/637889b1eddc36c11ad9). Additional data are available from the corresponding authors upon reasonable request. Source data are provided with this paper.

## Source data

Source Data
