# Supplementary material for: Alcohols electrooxidation coupled with H2 production at high current densities promoted by a cooperative catalyst
Source: Nat Commun. 2022 Jan 10;13:147. doi: 10.1038/s41467-021-27806-3 (PMC8748678; doi:10.1038/s41467-021-27806-3)
Supplement: Supplementary file 1 — Supplementary Information [file 41467_2021_27806_MOESM1_ESM.pdf]

Supplementary Information

Alcohols Electrooxidation Coupled with H_2 Production at High Current Densities Promoted by a Cooperative Catalyst

Zhenhua Li[1], Yifan Yan[1], Si-Min Xu[1], Hua Zhou[2], Ming Xu[1], Lina Ma[1], Mingfei Shao[1], Xianggui Kong[1], Bin Wang[3], Lirong Zheng[4] and Haohong Duan[2]*

[1]State Key Laboratory of Chemical Resource Engineering, College of Chemistry, Beijing University of Chemical Technology, Beijing 100029, China

[2]Department of Chemistry, Tsinghua University, Beijing 100084, China

[3]Beijing Research Institute of Chemical Industry, Sinopec Group, Beijing 100013, China

[4]Institute of High Energy Physics, the Chinese Academy of Sciences, Beijing 100049, China

Zhenhua Li, Yifan Yan, Si-Min Xu and Hua Zhou contributed equally to this work.

Correspondence and requests for materials should be addressed to H.D. (email: hhduan@mail.tsinghua.edu.cn).

Supplementary figures

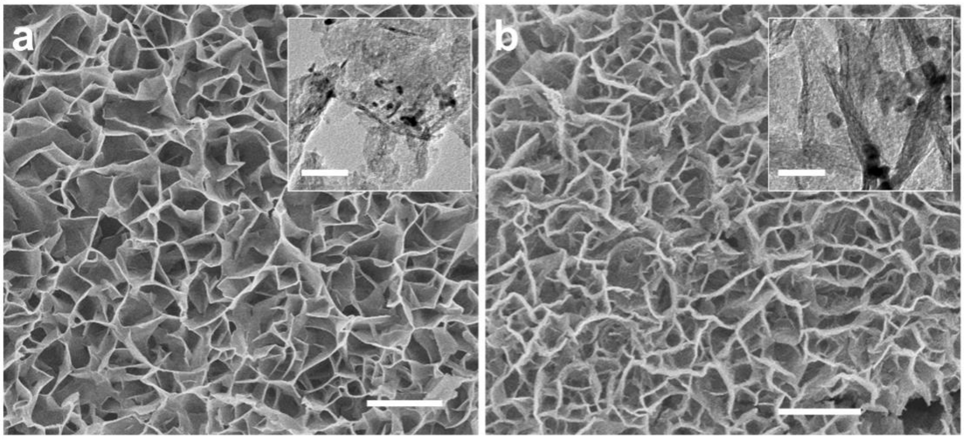

Supplementary Figure 1 | Characterisations of different catalysts. SEM images of (**a**) CoOOH, (**b**) NiFeOOH on glassy carbon. Scale bars, 1 μm. Inset displays the corresponding HRTEM images. Scale bars, 30 nm.

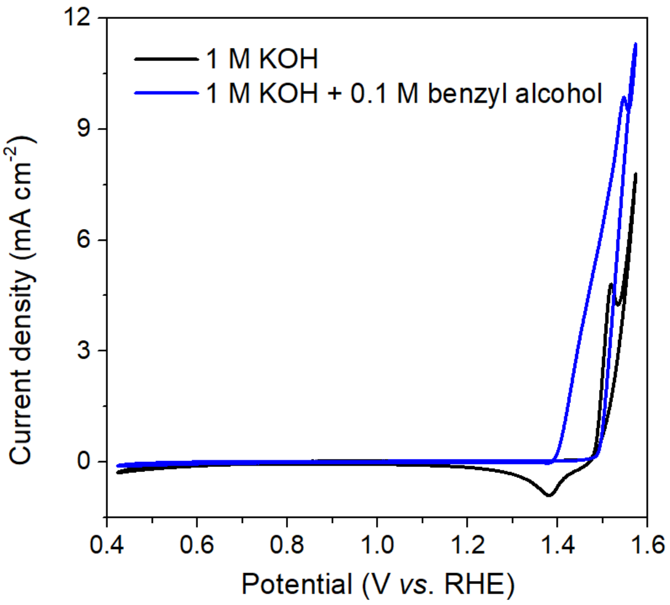

Supplementary Figure 2 | Electrochemical performances. CV curves of NiFeOOH on glassy carbon in 1 M KOH and 1 M KOH with 0.1 M benzyl alcohol at scan rate of 10 mV s^{-1}.

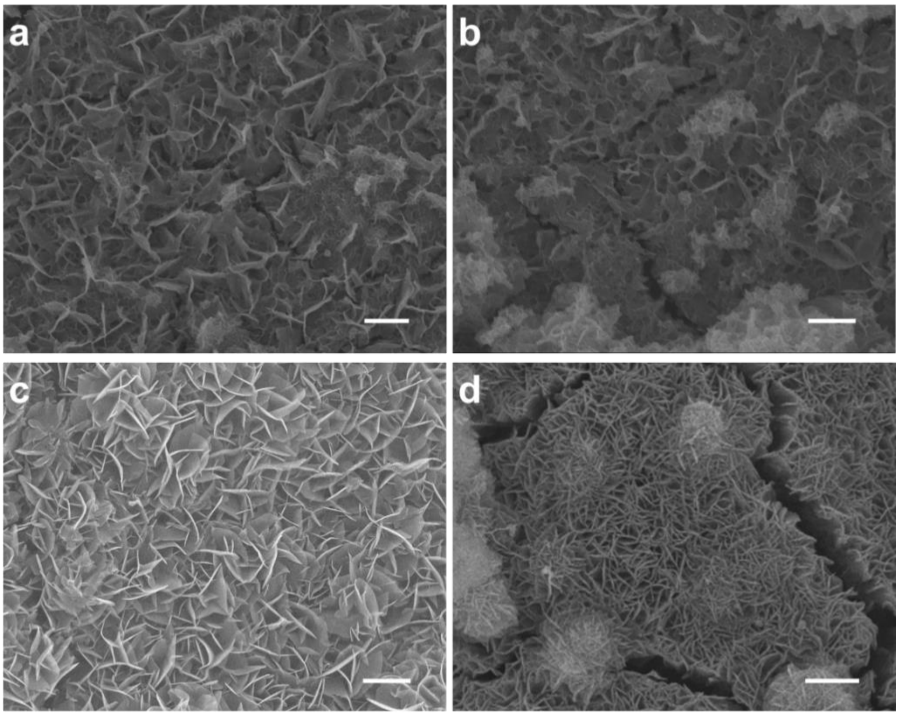

Supplementary Figure 3 | Characterisations of different catalysts. SEM images of (**a**) Au/NiOOH, (**b**) Au/FeOOH, (**c**) Au/CoOOH and (**d**) Au/NiFeOOH on Ni foam. Scale bars, 1 μm.

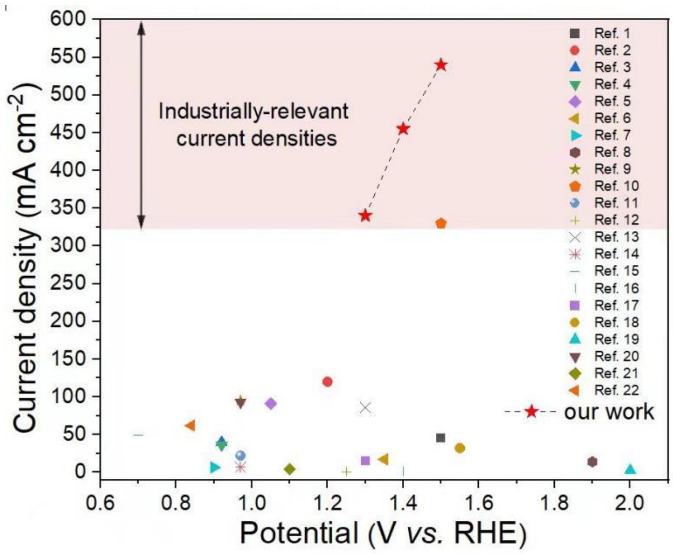

Supplementary Figure 4 | Comparison of the catalytic performances. Current densities of anodic oxidation reactions over Au-based catalysts reported in the literatures and the benzyl alcohol oxidation in this work.

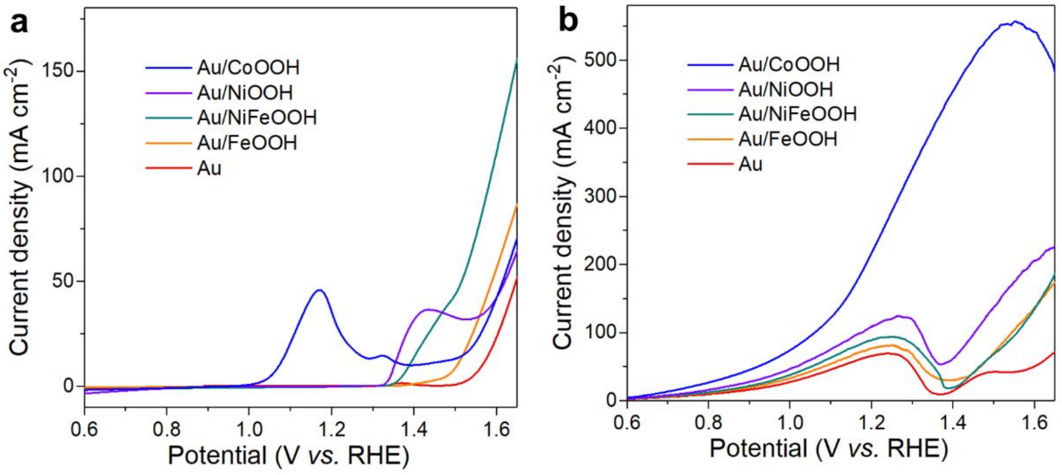

Supplementary Figure 5 | Electrochemical performances. LSV curves of Au and Au/MOOHs catalysts at scan rate of 10 mV s^{-1} in (**a**) 1 M KOH and (**b**) 1 M KOH with 0.1 M benzyl alcohol at r.t.

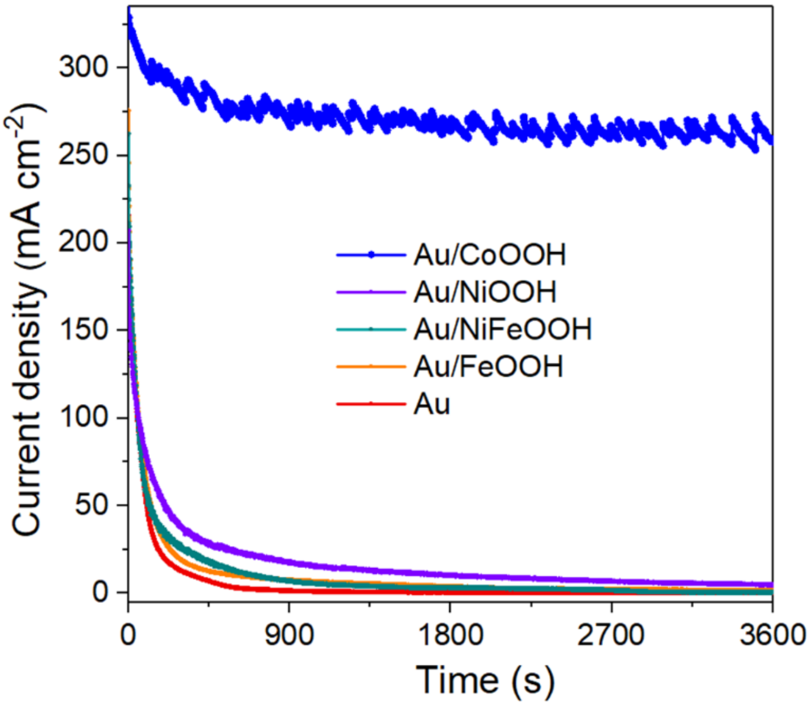

Supplementary Figure 6 | Electrochemical performances. Current-time (*I*-*t*) curves of Au and Au/MOOH samples in 1 M KOH with 0.1 M benzyl alcohol at 1.3 V *vs*. RHE.

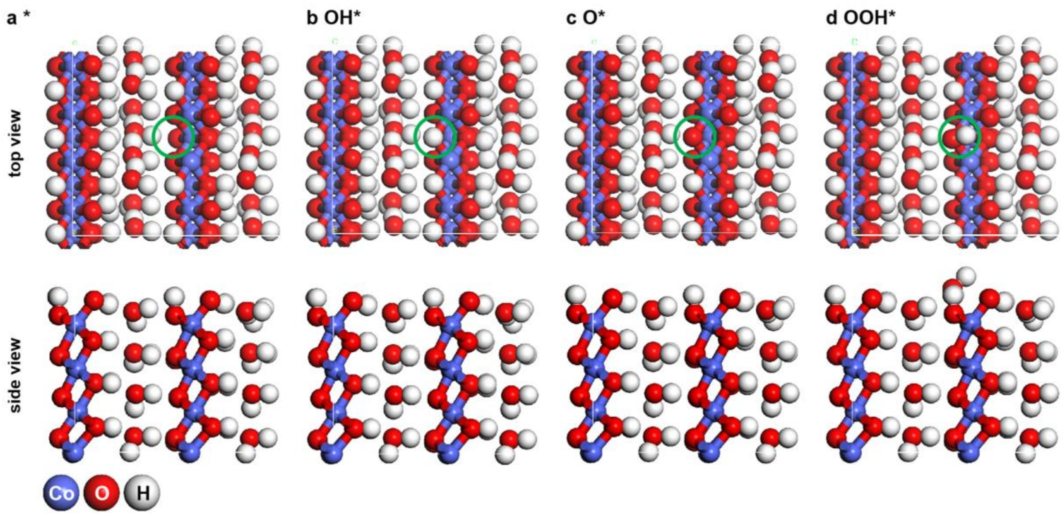

Supplementary Figure 7 | Optimized geometries of OER reaction intermediates. Optimized geometries of reaction intermediates (* (**a**), OH* (**b**), O* (**c**), and OOH* (**d**)) in oxygen evolution reaction over CoOOH. The reaction active site is highlighted with green cycle. The color of each element is also labeled.

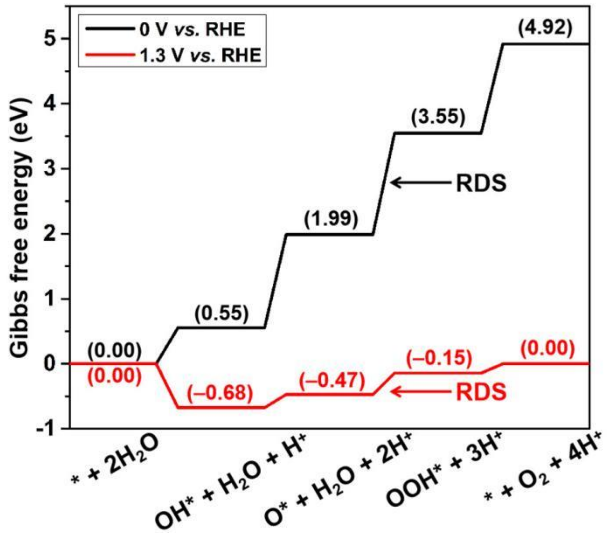

Supplementary Figure 8 | Gibbs free energy diagrams. Gibbs free energy diagrams for oxygen evolution reaction over CoOOH at 0 V and 1.3 V *vs.* RHE. The RDS in oxygen evolution reaction is labeled.

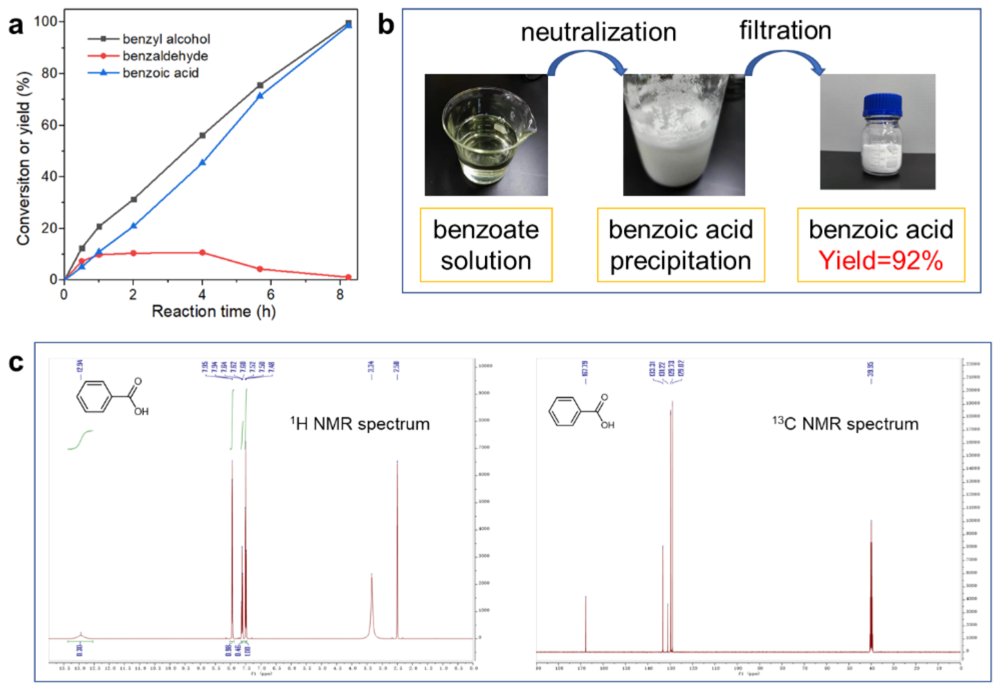

Supplementary Figure 9 | Product separation. a, The kinetic curves for benzyl alcohol conversion as a function of reaction time over Au/CoOOH in 1 M KOH with 0.1 M benzyl alcohol at 1.3 V *vs*. RHE at r.t. **b**, Collection of pure benzoic acid. **c**, The ^{1}H and ^{13}C NMR spectra of the obtained product showing the purity of benzoic acid.

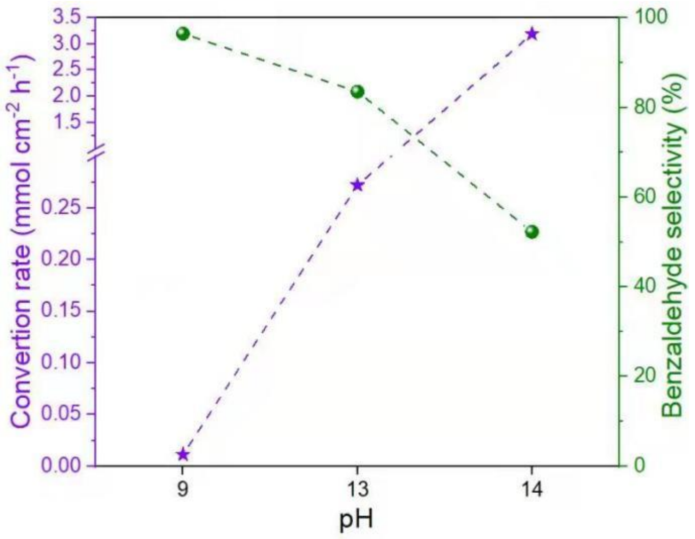

Supplementary Figure 10 | Benzaldehyde selectivity at different pH value. The conversion rate of benzyl alcohol and corresponding benzaldehyde selectivity of Au/CoOOH at KOH electrolyte (with different pH) + 0.1 M benzyl alcohol at 1.3 V *vs*. RHE for 2h.

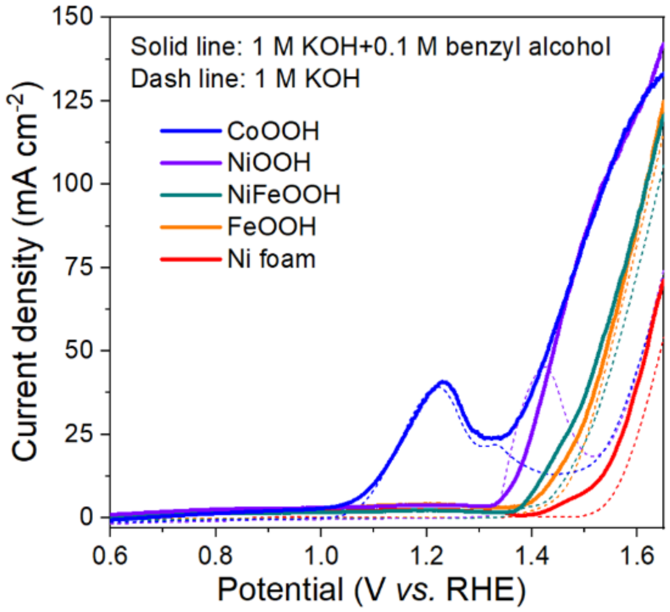

Supplementary Figure 11 | Electrochemical performances. LSV curves of Au and Ni foam and MOOH supports at scan rate of 10 mV s^{-1} in 1 M KOH with 0.1 M benzyl alcohol at r.t.

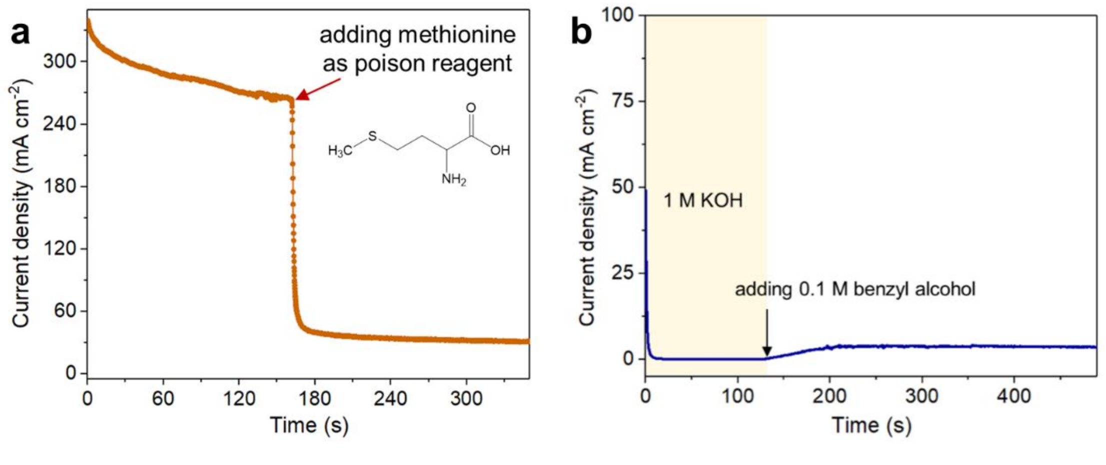

Supplementary Figure 12 | Verification the reason of catalyst inactivation. a, *I-t* curve of Au/CoOOH at 1.3 V *vs*. RHE in 1 M KOH with 0.1 M benzyl alcohol at r.t. (methionine was added at 160 s). **b**, *I-t* curve of Au/CoOOH at 1.3 V *vs*. RHE in 1 M KOH with 0.1 M benzyl alcohol at r.t. (benzyl alcohol was added at 130 s).

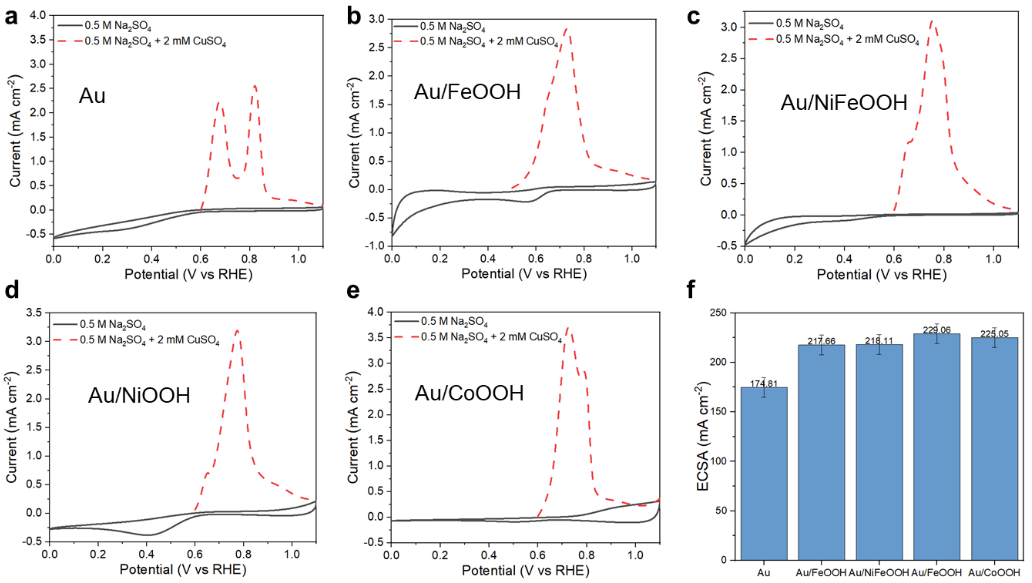

Supplementary Figure 13 | Electrochemical active surface area. Underpotential deposition of Cu for measuring the ECSA of (**a**) Au, (**b**) Au/FeOOH, (**c**) Au/NiFeOOH, (**d**) Au/NiOOH and (**e**) Au/CoOOH. Scan rate: 5 mV s^{-1}. (**f**) ECSA value of Au and Au/MOOH.

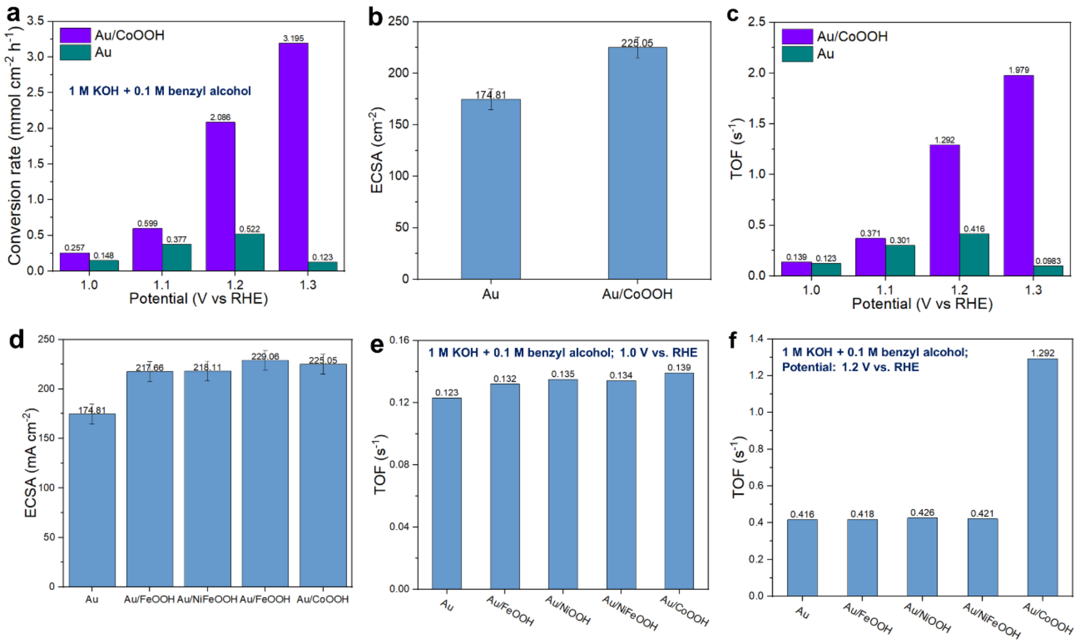

Supplementary Figure 14 | TOF value. a, Benzyl alcohol conversion rate over Au and Au/CoOOH catalysts in 1 M KOH with 0.1 M benzyl alcohol at different potential. **b,** The ECSA of Au and Au/CoOOH catalysts. **c,** The calculated TOF values of Au and Au/CoOOH in 1 M KOH with 0.1 M benzyl alcohol at different potential. **d,** The ECSA of Au and Au/MOOH catalysts. **e,f** The calculated TOF values of Au and Au/CoOOH in 1 M KOH with 0.1 M benzyl alcohol at (**b**) 1.0 V *vs*. RHE and (**c**) 1.2 V *vs*. RHE.

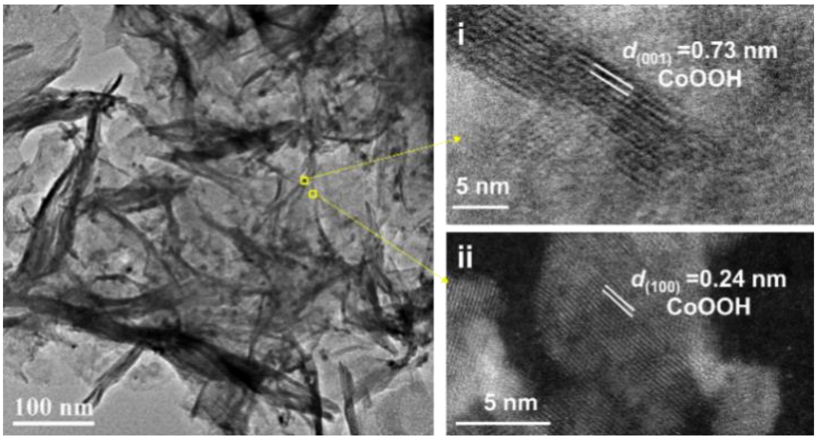

Supplementary Figure 15 | Characterisations of Au/CoOOH. TEM image of Au/CoOOH and the HRTEM images of CoOOH region with exposed **i** (001) and **ii** (100) crystal plane.

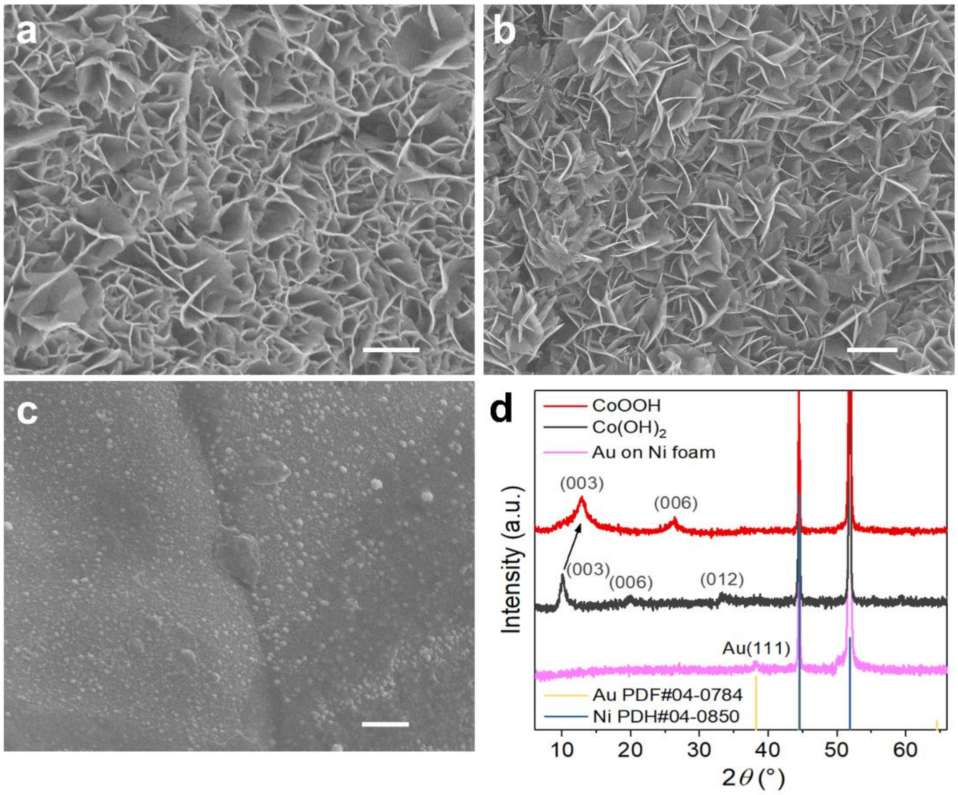

Supplementary Figure 16 | Characterisations of different catalysts. SEM images of (**a**) $Co(OH)_2$, (**b**) CoOOH and (**c**) Au on Ni foam. Scale bars, 1 µm. (**d**) XRD patterns of $Co(OH)_2$, CoOOH and Au on Ni foam.

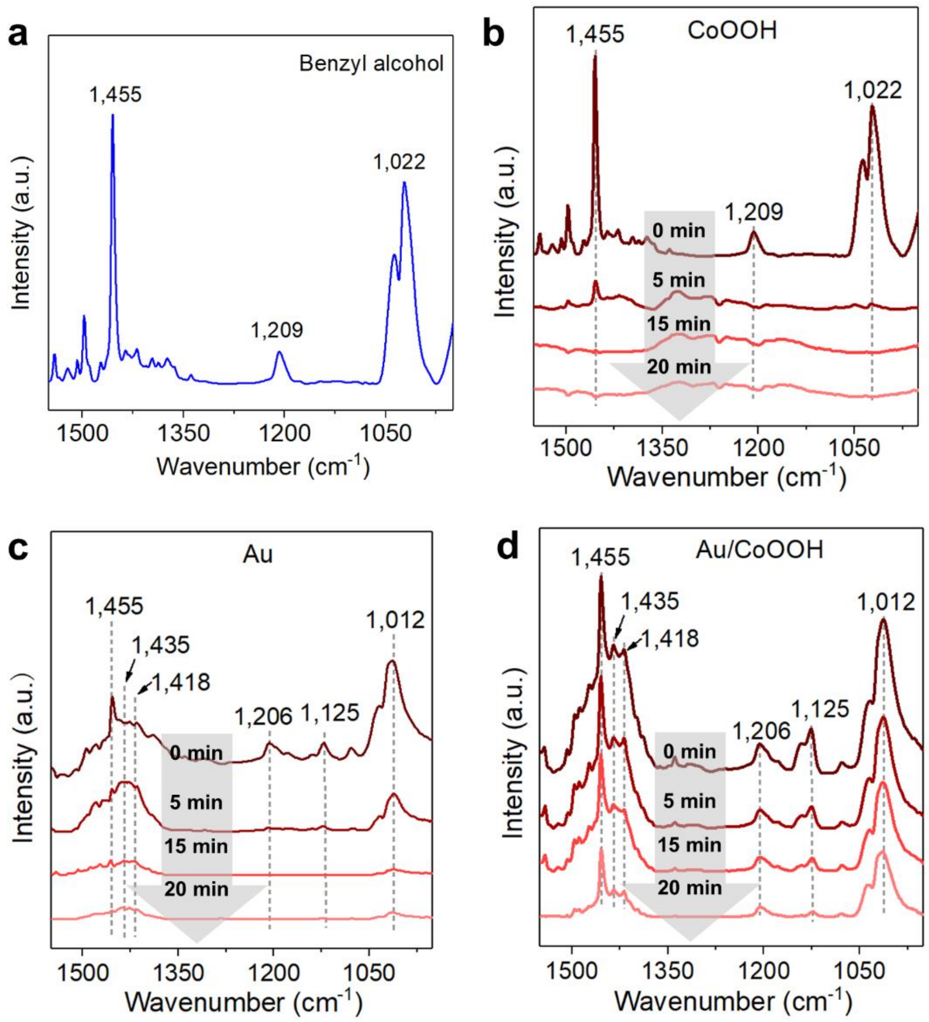

Supplementary Figure 17 | FTIR spectra. a, FTIR spectra of gaseous benzyl alcohol. **b-d**, FTIR spectra of (**b**) CoOOH, (**c**) Au and (**d**) Au/CoOOH after benzyl alcohol adsorption and 15 min of He purging.

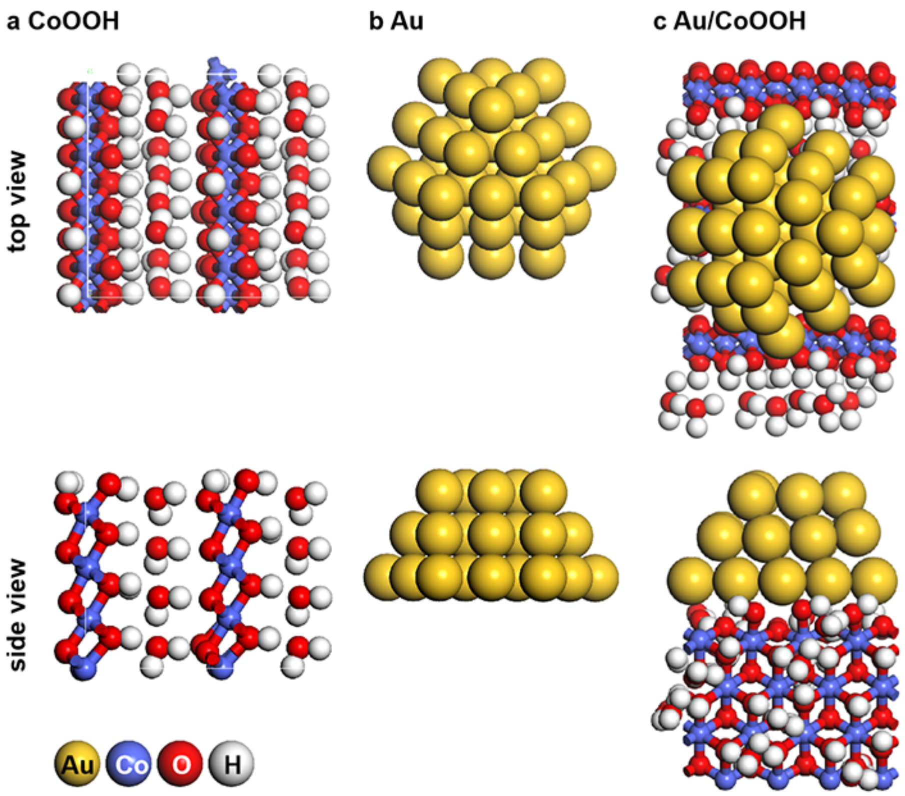

Supplementary Figure 18 | Optimized geometries of models. Optimized geometries of models CoOOH (**a**), Au (**b**) and Au/CoOOH (**c**), respectively. The color of each element is labeled.

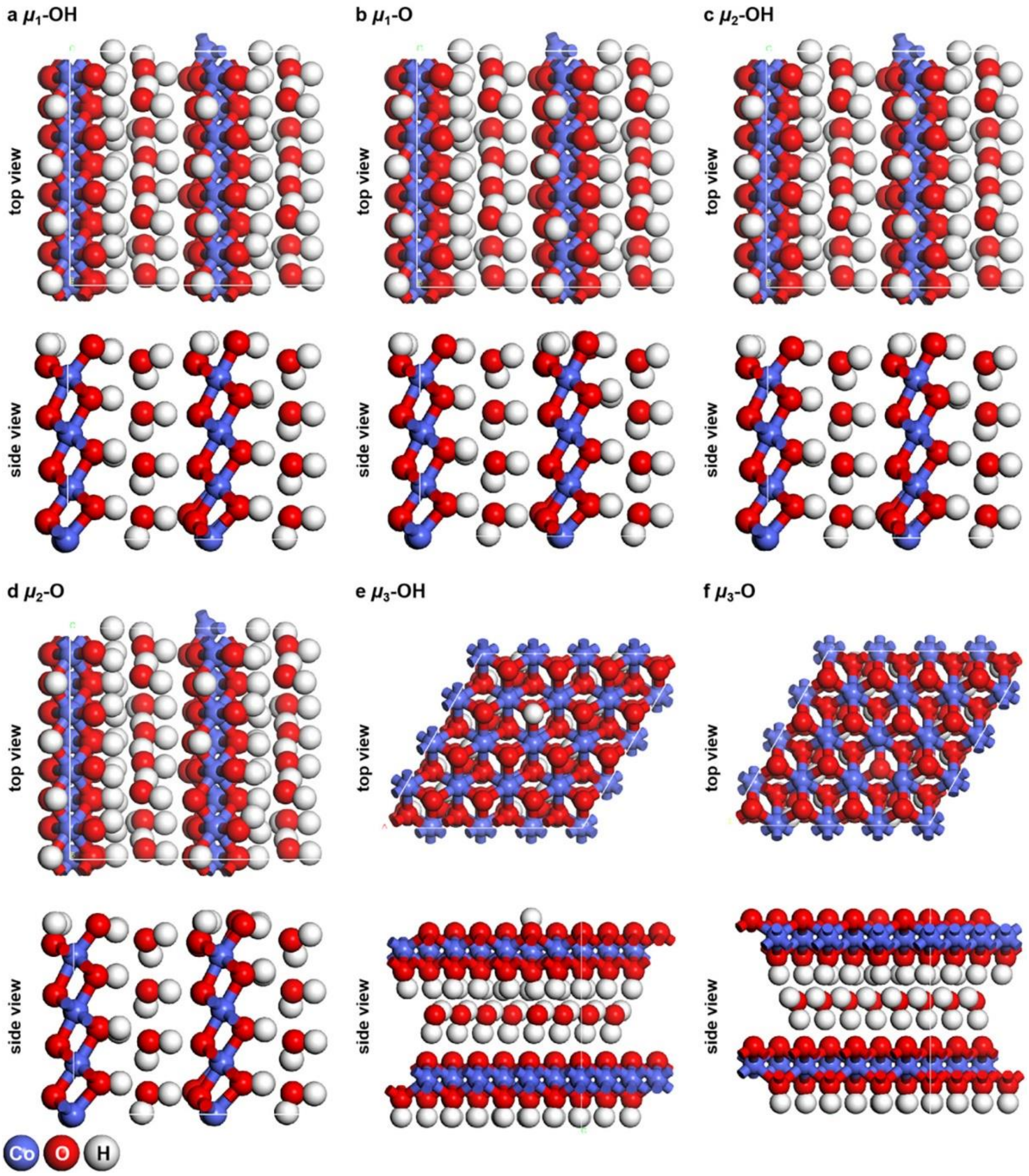

Supplementary Figure 19 | Optimized geometries of CoOOH models. Optimized geometries of μ_1–OH (**a**), μ_1–O (**b**), μ_2–OH (**c**), μ_2–O (**d**), μ_3–OH (**e**), and μ_3–O (**f**) for CoOOH in top and side views. The color for each element is labeled.

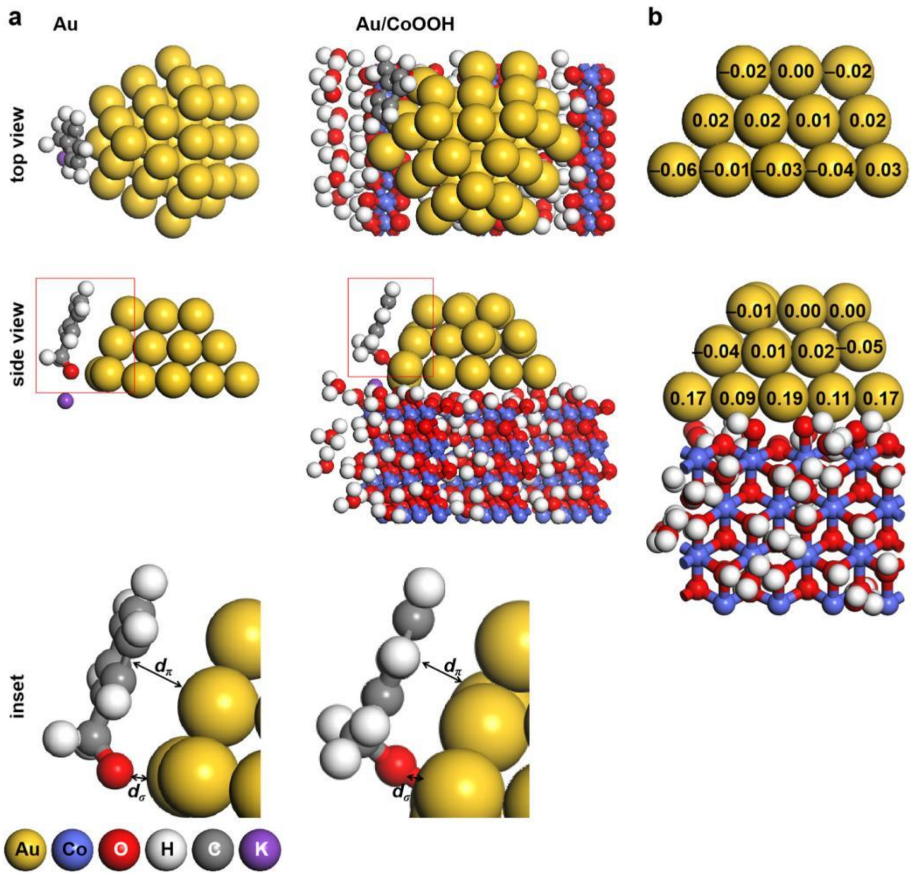

Supplementary Figure 20 | Benzyl alkoxide adsorption. a, Schematic illustrations for the adsorption configurations of benzyl alkoxide on Au and Au/CoOOH. The distance between the phenyl group and Au (111) facet is identified as d_{π}, and that between the O atom and bonded Au atom is identified as d_{σ}. The color for each element is labeled. **b**, Hirshfeld charges of Au atoms in models Au and Au/CoOOH in the unit of *e*, respectively.

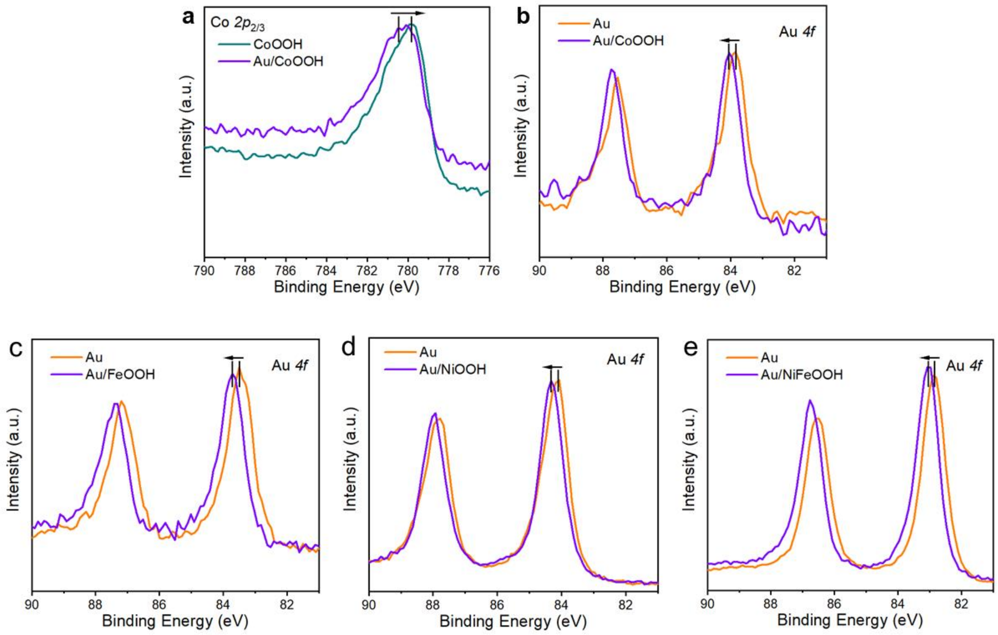

Supplementary Figure 21 | XPS spectra. High-resolution (**a**) Co $2p_{2/3}$ XPS spectra of CoOOH and Au/CoOOH. **b-e** High-resolution Au 4*f* XPS spectra of Au and (**b**) Au/CoOOH, (**c**) Au/FeOOH, (**d**) Au/NiOOH and (**e**) Au/NiFeOOH.

The binding energy of Co $2p_{3/2}$ peak for Au/CoOOH (Supplementary Fig. 21a) shows a shift to higher energy (~0.1 eV) in comparison with pure CoOOH sample (780.08 eV), suggesting the combination of Au NPs reduces the valence state of Co^{3+}. In the case of Au NPs, the Au 4*f* XPS spectra of Au/CoOOH (Supplementary Fig. 21b) shows a positive peak shift than that of Au, further confirming the interface binding and electron transfer between CoOOH and Au NPs. Au 4*f* XPS spectra of Au/FeOOH, Au/NiOOH and Au/NiFeOOH also shows positive peak shift than that of Au (Supplementary Fig. 21c-e), suggesting that CoOOH and other MOOH has similar electronic interaction with Au.

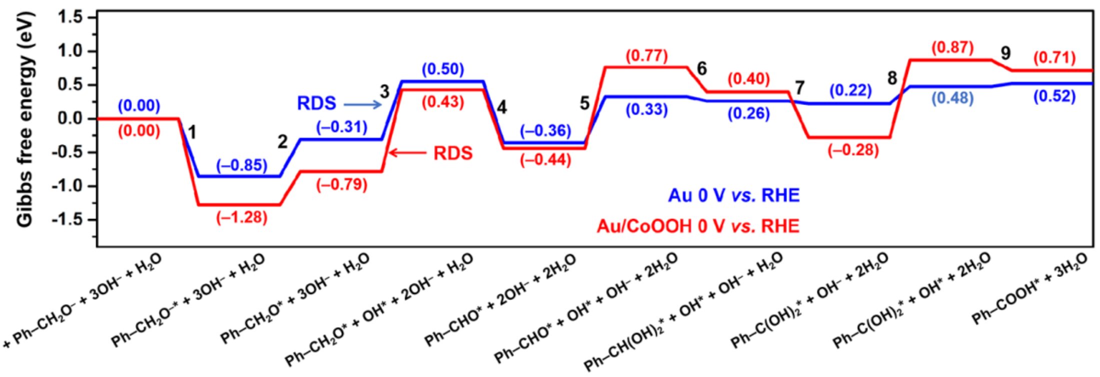

Supplementary Figure 22 | Gibbs free energy diagrams. Gibbs free energy diagrams for benzyl alcohol oxidation to benzoic acid on Au and Au/CoOOH at 0 V *vs.* RHE. The values of Gibbs free energies are labeled in the bracket in the unit of eV. The serial numbers for the elementary steps are also labeled.

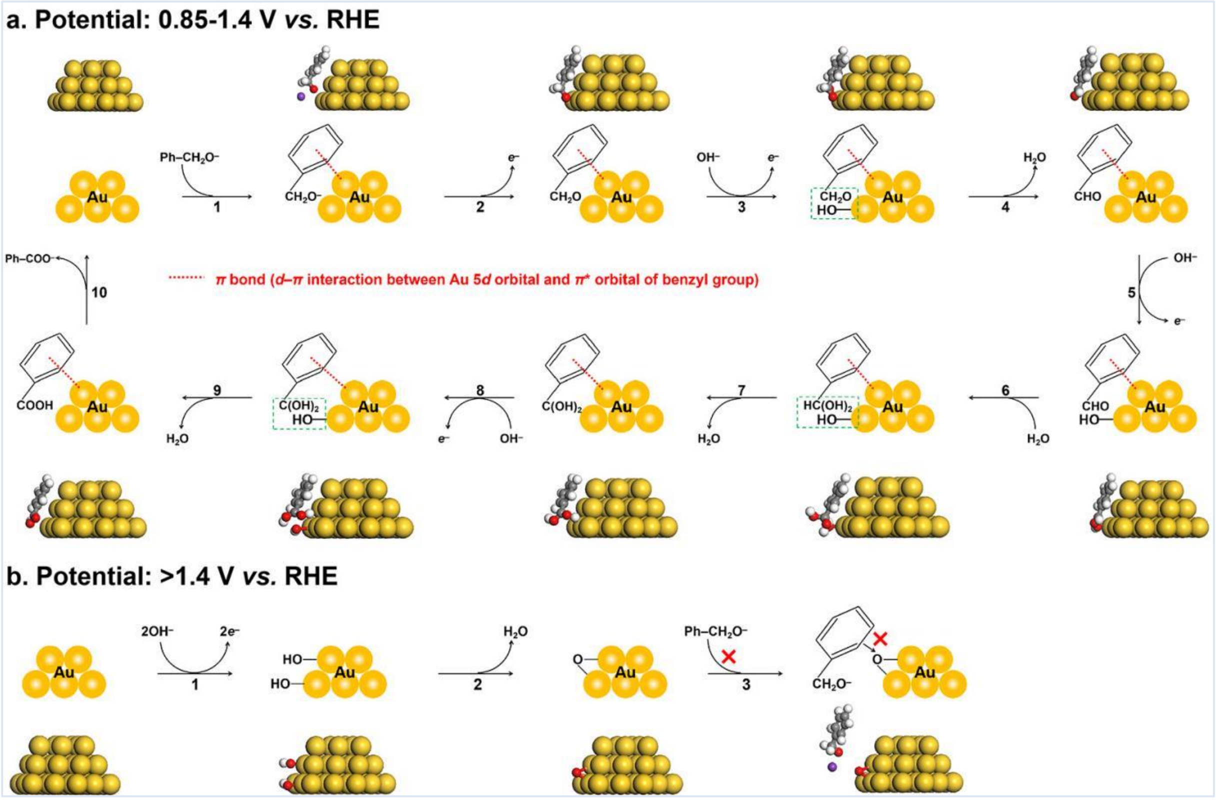

Supplementary Figure 23 | Reaction mechanism over Au. Schematic illustrations of the reaction processes for benzyl alcohol oxidation on Au at potentials of 0.85-1.4 V *vs.* RHE (**a**) and >1.4 V *vs.* RHE (**b**).

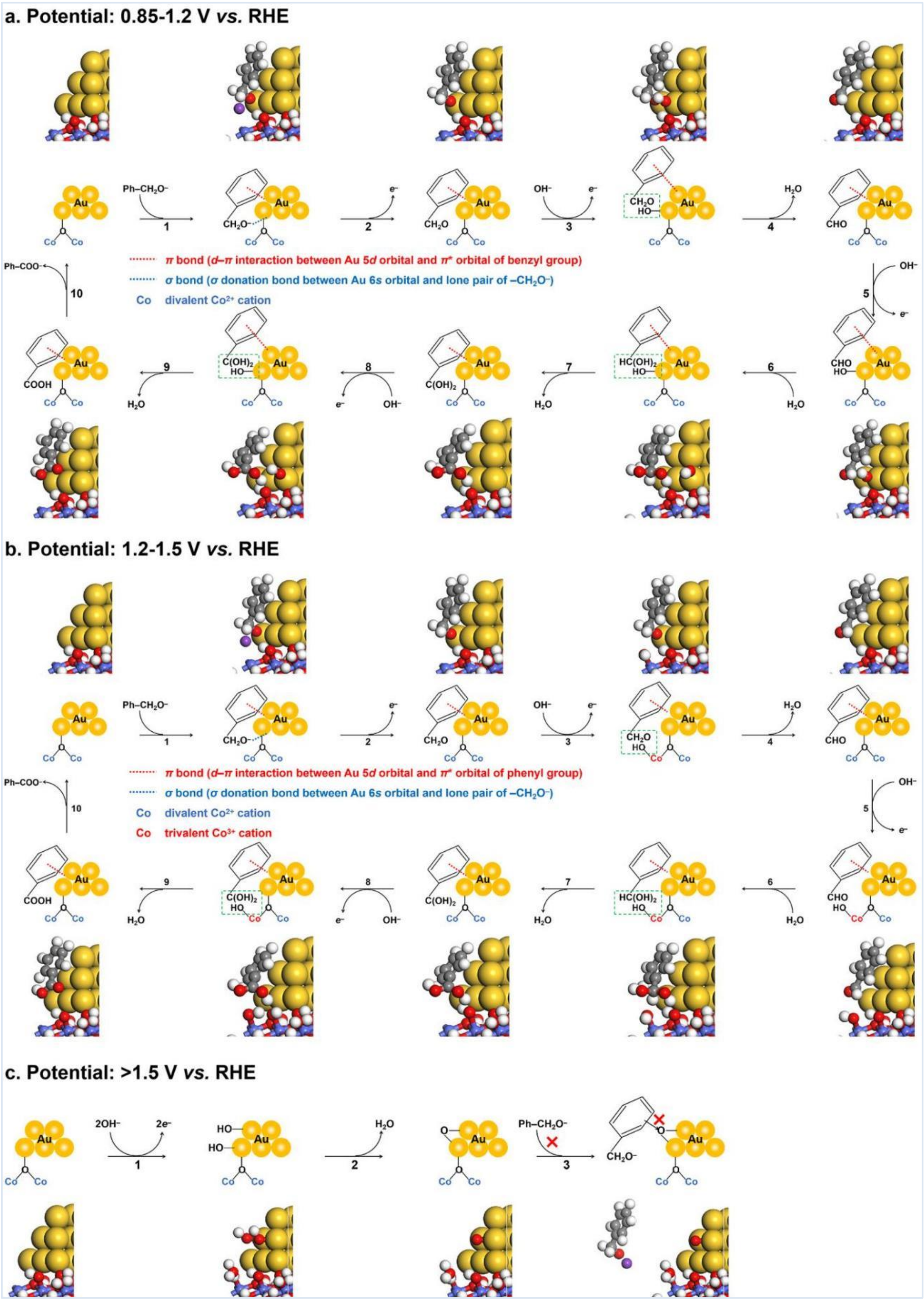

Supplementary Figure 24 | Reaction mechanism over Au/CoOOH. Schematic illustrations of the reaction processes for benzyl alcohol oxidation on Au/CoOOH at potentials of 0.85-1.2 V *vs.* RHE (**a**), 1.2-1.5 V *vs.* RHE (**b**), and >1.5 V *vs.* RHE (**c**).

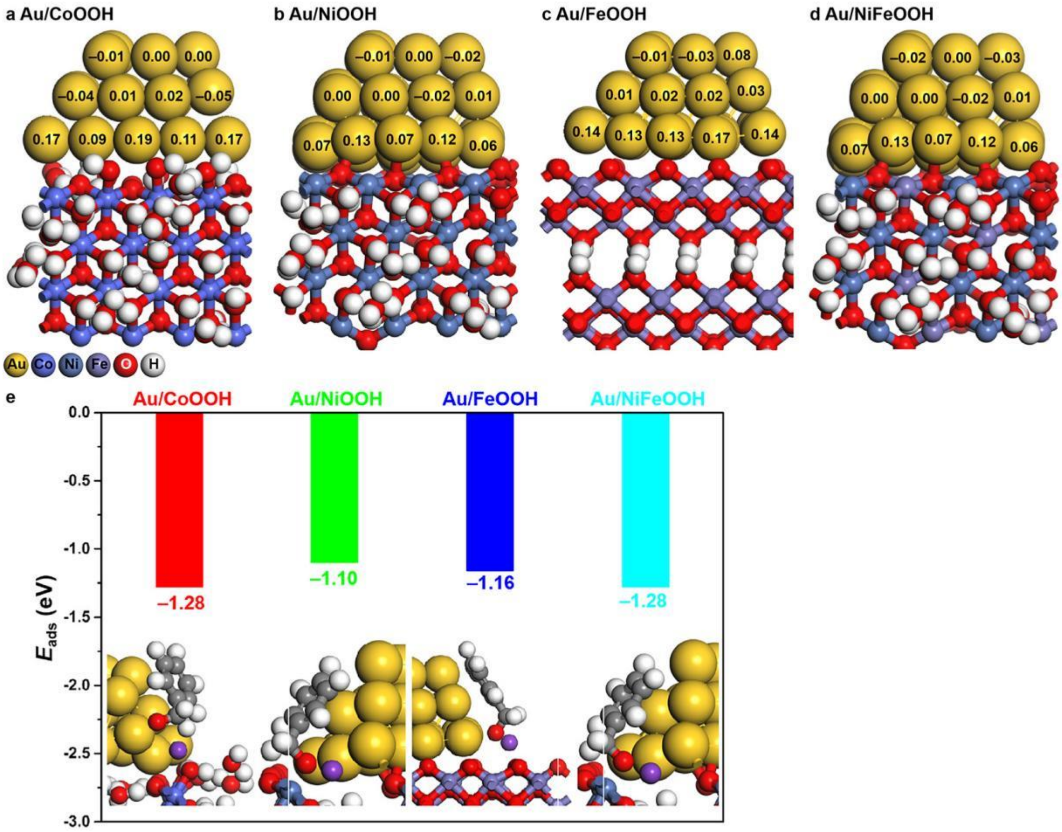

Supplementary Figure 25 | Electronic structure interaction at the interface of Au/MOOH. **a-d**, Hirshfeld charge analyses of Au/CoOOH (**a**), Au/NiOOH (**b**), Au/FeOOH (**c**), and Au/NiFeOOH (**d**), respectively. The Hirshfeld charges of Au atoms are labeled in the unit of *e*. The color for each element is also labeled. **e**, Adsorption energies of Ph–CH_2O^- on Au/CoOOH, Au/NiOOH, Au/FeOOH, and Au/NiFeOOH, respectively. The optimized geometries of Ph–CH_2O^-* are also displayed.

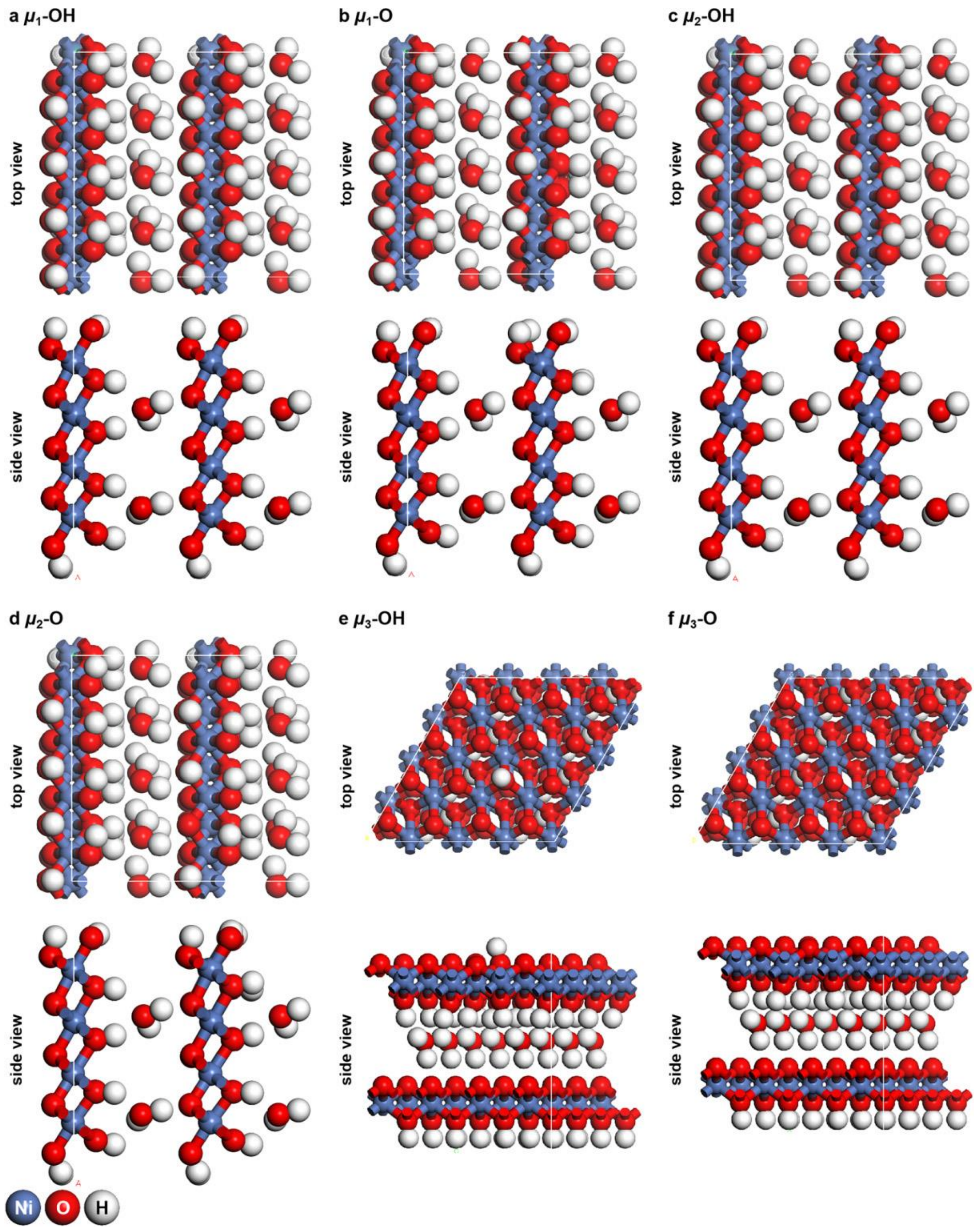

Supplementary Figure 26 | Optimized geometries of NiOOH models. Optimized geometries of μ_1–OH (**a**), μ_1–O (**b**), μ_2–OH (**c**), μ_2–O (**d**), μ_3–OH (**e**), and μ_3–O (**f**) for NiOOH in top and side views.

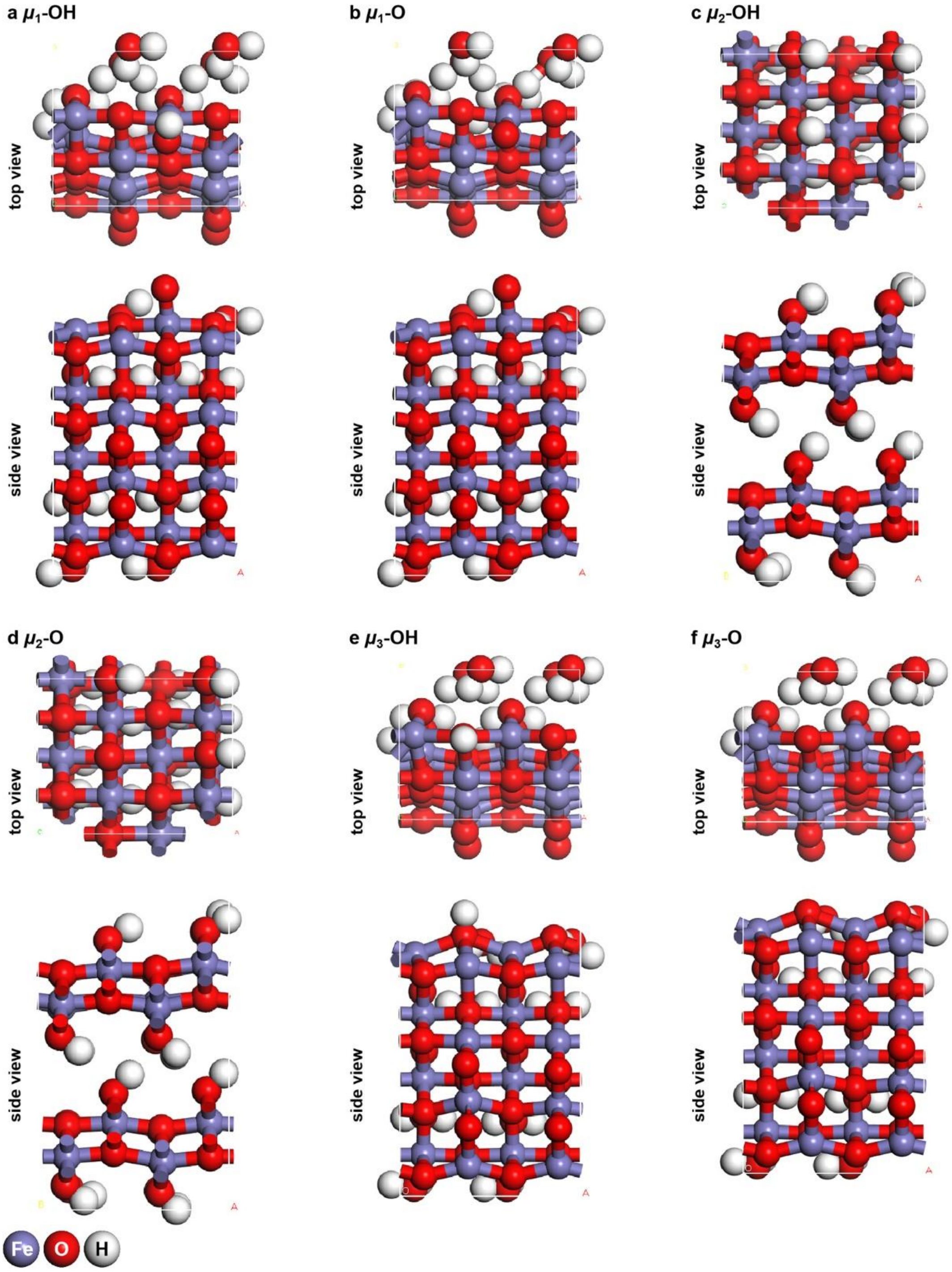

Supplementary Figure 27 | Optimized geometries of FeOOH models. Optimized geometries of μ_1–OH (**a**), μ_1–O (**b**), μ_2–OH (**c**), μ_2–O (**d**), μ_3–OH (**e**), and μ_3–O (**f**) for FeOOH in top and side views. The color of each element is also labeled.

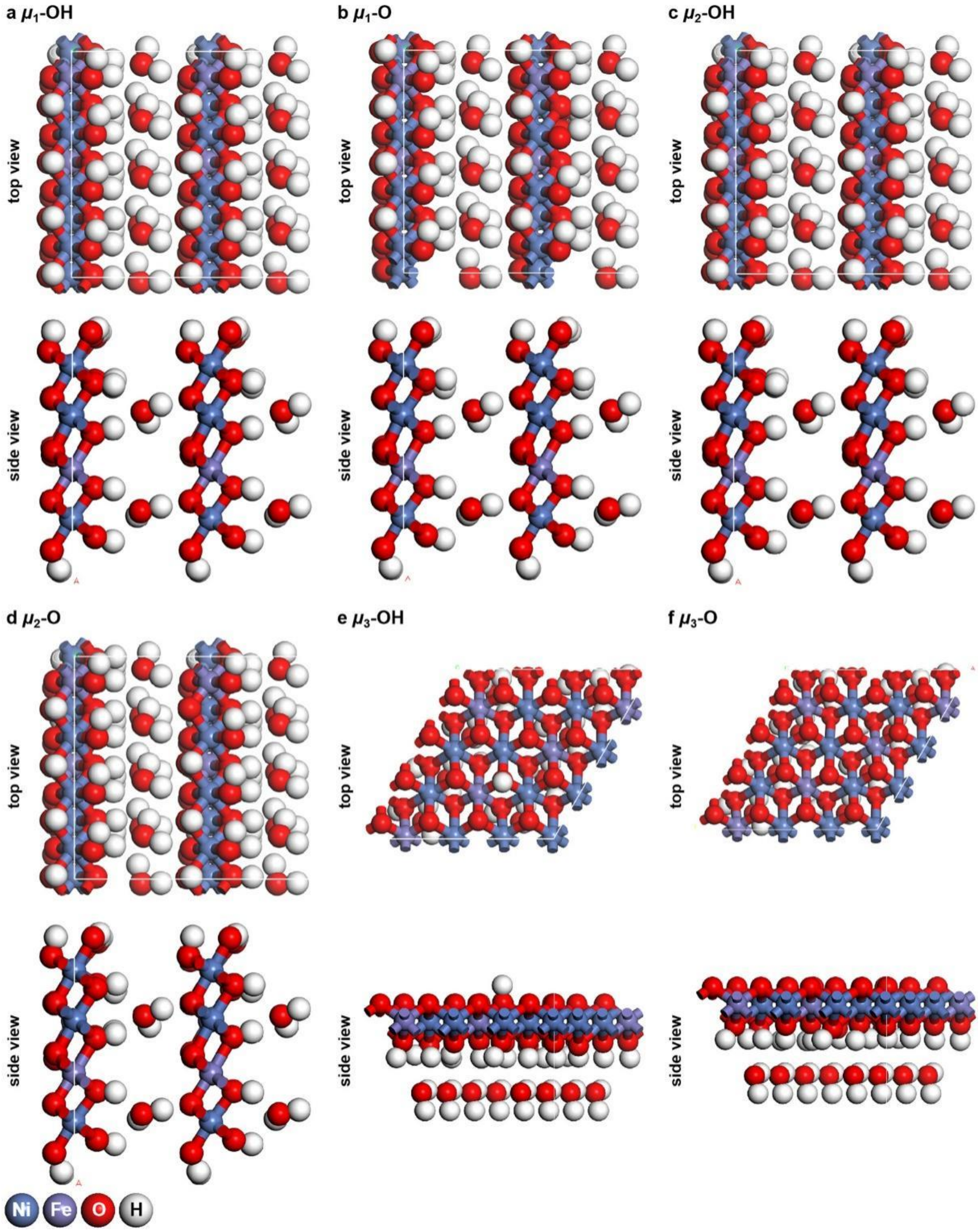

Supplementary Figure 28 | Optimized geometries of NiFeOOH models. Optimized geometries of μ_1–OH (**a**), μ_1–O (**b**), μ_2–OH (**c**), μ_2–O (**d**), μ_3–OH (**e**), and μ_3–O (**f**) for NiFeOOH in top and side views. The color for each element is also labeled.

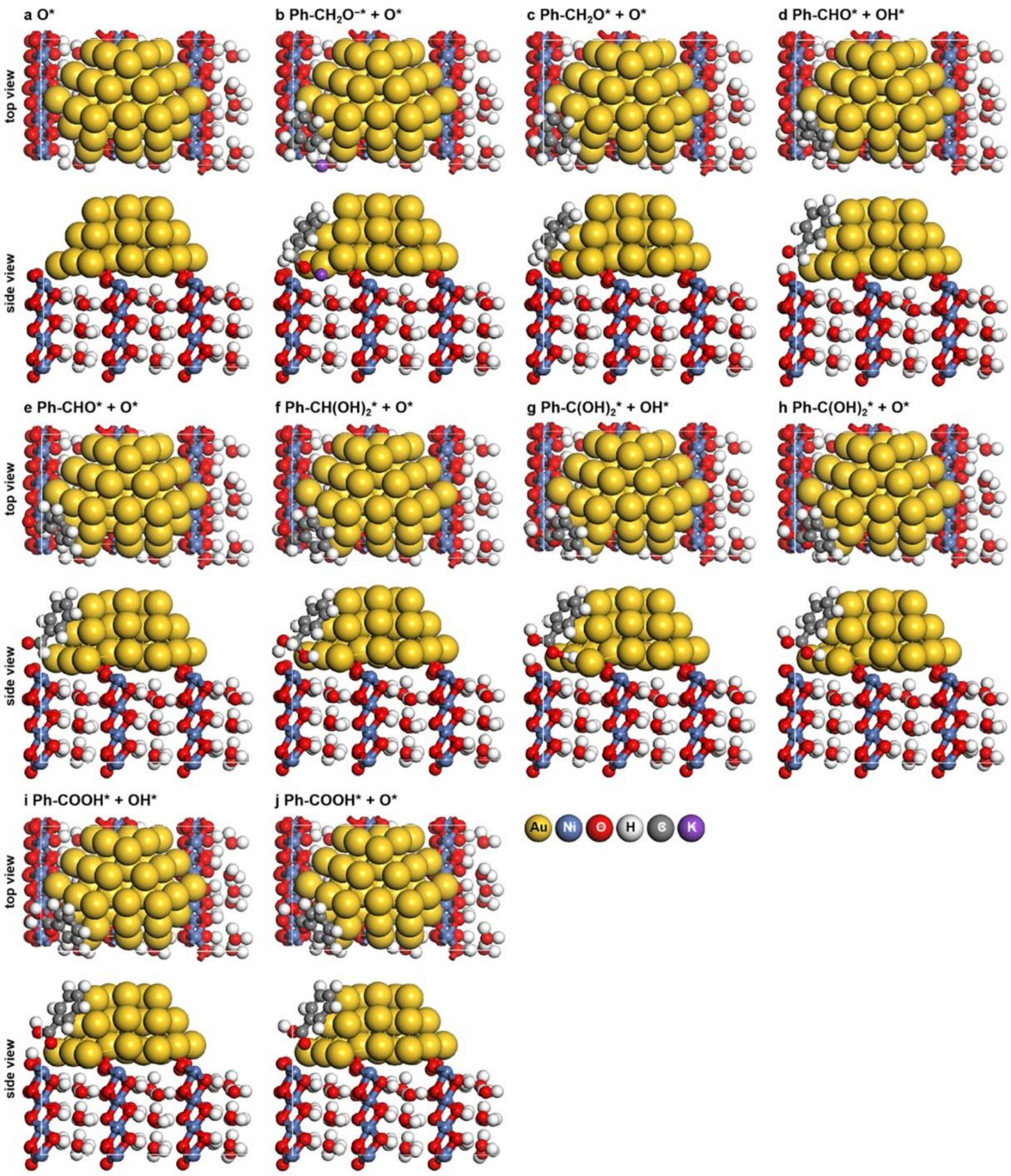

Supplementary Figure 29 | Optimized geometries of the reaction intermediates over Au/NiOOH. Optimized geometries of the reaction intermediates (O* (**a**), Ph–CH_2O^{-}* + O* (**b**), Ph–CH_2O* + O* (**c**), Ph–CHO* + OH* (**d**), Ph–CHO* + O* (**e**), Ph–$CH(OH)_2$* + O* (**f**), Ph–$C(OH)_2$* + OH* (**g**), Ph–$C(OH)_2$* + O* (**h**), Ph–COOH* + OH* (**i**), and Ph–COOH* + O* (**j**)) for benzyl alcohol oxidation to benzoic acid on Au/NiOOH in top and side views. The color for each element is also labeled.

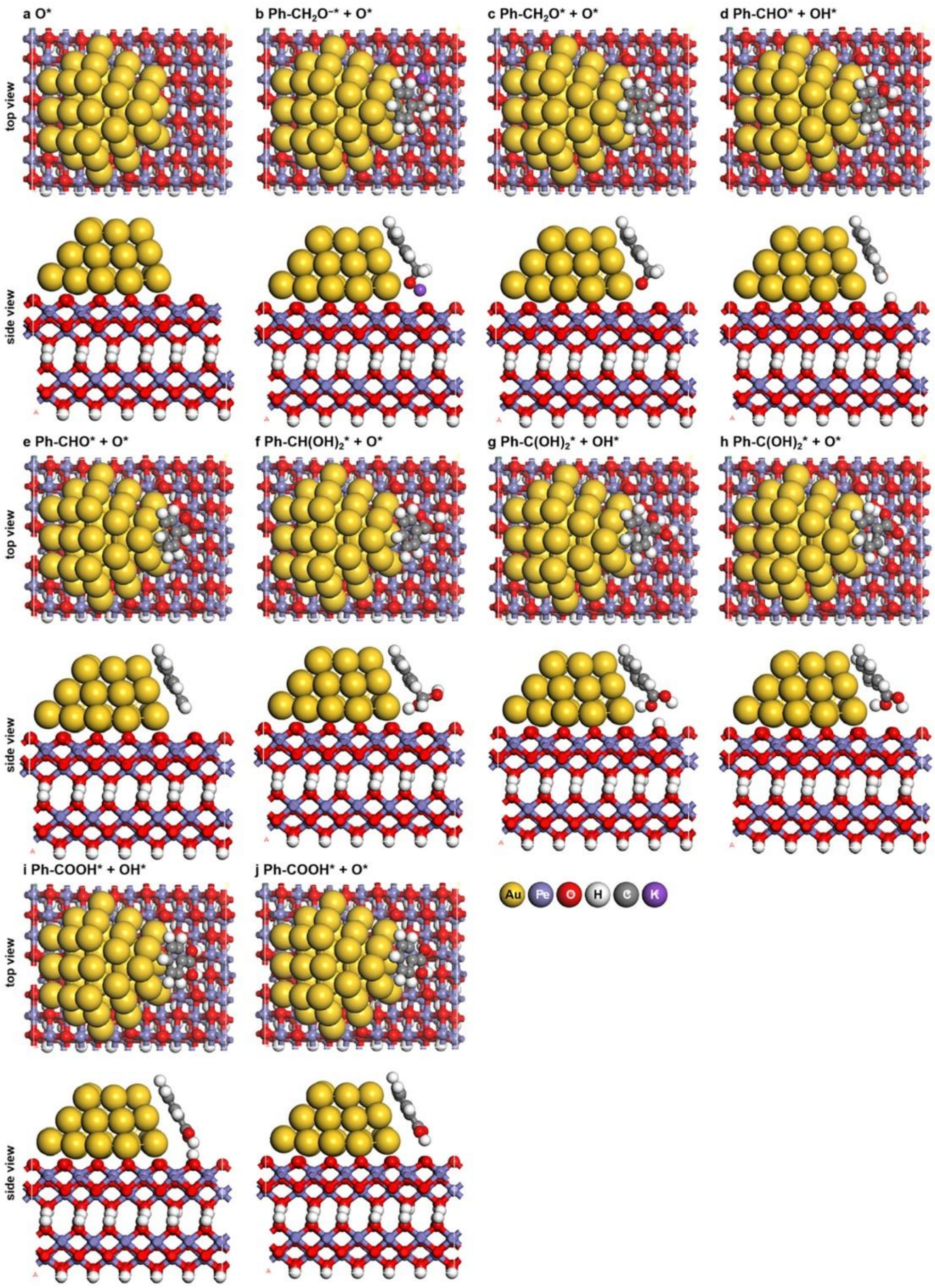

Supplementary Figure 30 | Optimized geometries of the reaction intermediates over Au/FeOOH. Optimized geometries of the reaction intermediates (O* (**a**), Ph–CH$_2$O$^-$* + O* (**b**), Ph–CH$_2$O* + O* (**c**), Ph–CHO* + OH* (**d**), Ph–CHO* + O* (**e**), Ph–CH(OH)$_2$* + O* (**f**), Ph–C(OH)$_2$* + OH* (**g**), Ph–C(OH)$_2$* + O* (**h**), Ph–COOH* + OH* (**i**), and Ph–COOH* + O* (**j**)) for benzyl alcohol oxidation to benzoic acid on Au/FeOOH in top and side views. The color for each element is also labeled.

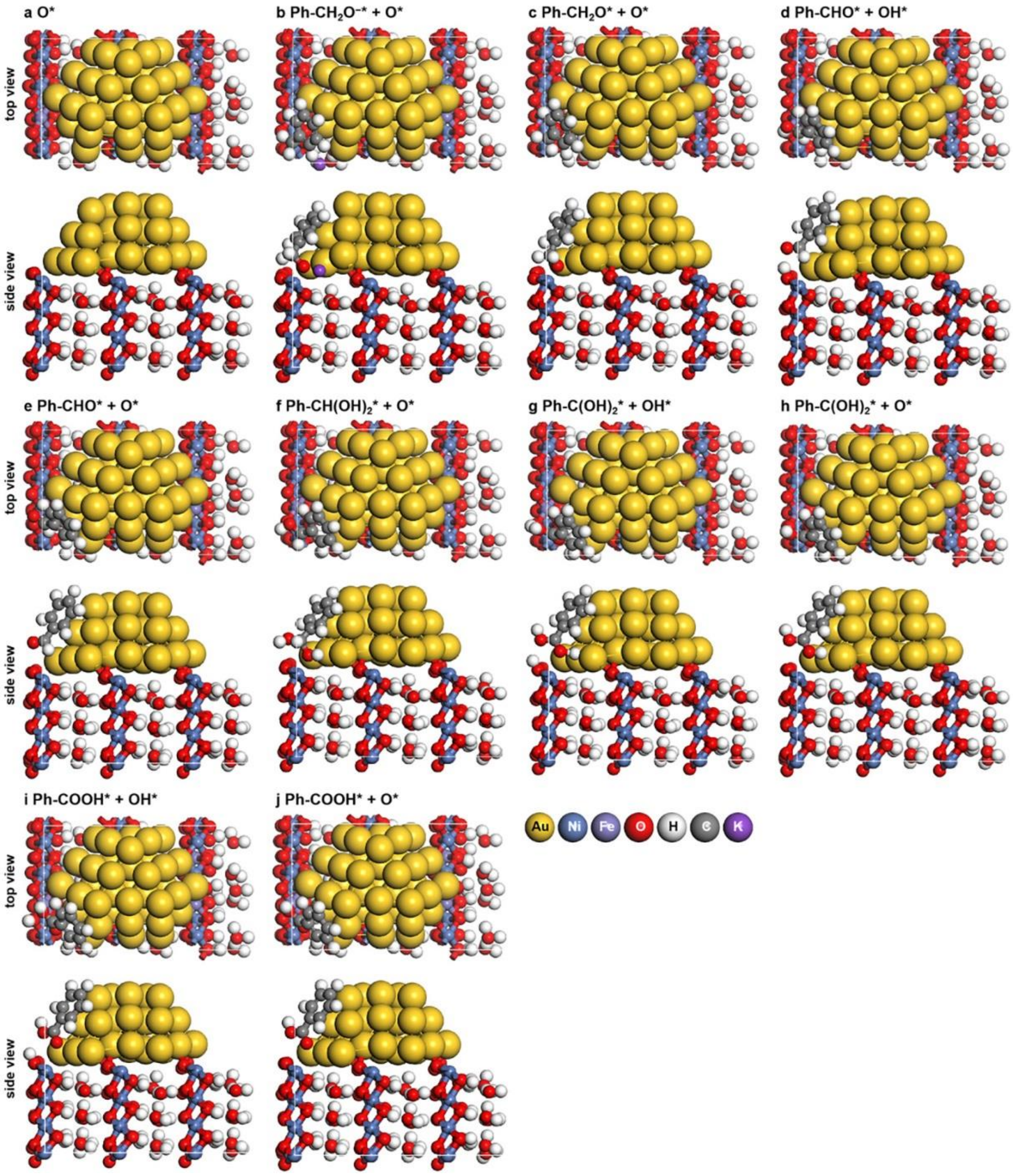

Supplementary Figure 31 | Optimized geometries of the reaction intermediates over Au/NiFeOOH. Optimized geometries of the reaction intermediates (O* (**a**), Ph–CH_2O^{-}* + O* (**b**), Ph–CH_2O* + O* (**c**), Ph–CHO* + OH* (**d**), Ph–CHO* + O* (**e**), Ph–$CH(OH)_2$* + O* (**f**), Ph–$C(OH)_2$* + OH* (**g**), Ph–$C(OH)_2$* + O* (**h**), Ph–COOH* + OH* (**i**), and Ph–COOH* + O* (**j**)) for benzyl alcohol oxidation to benzoic acid on Au/NiFeOOH in top and side views. The color of each element is also labeled.

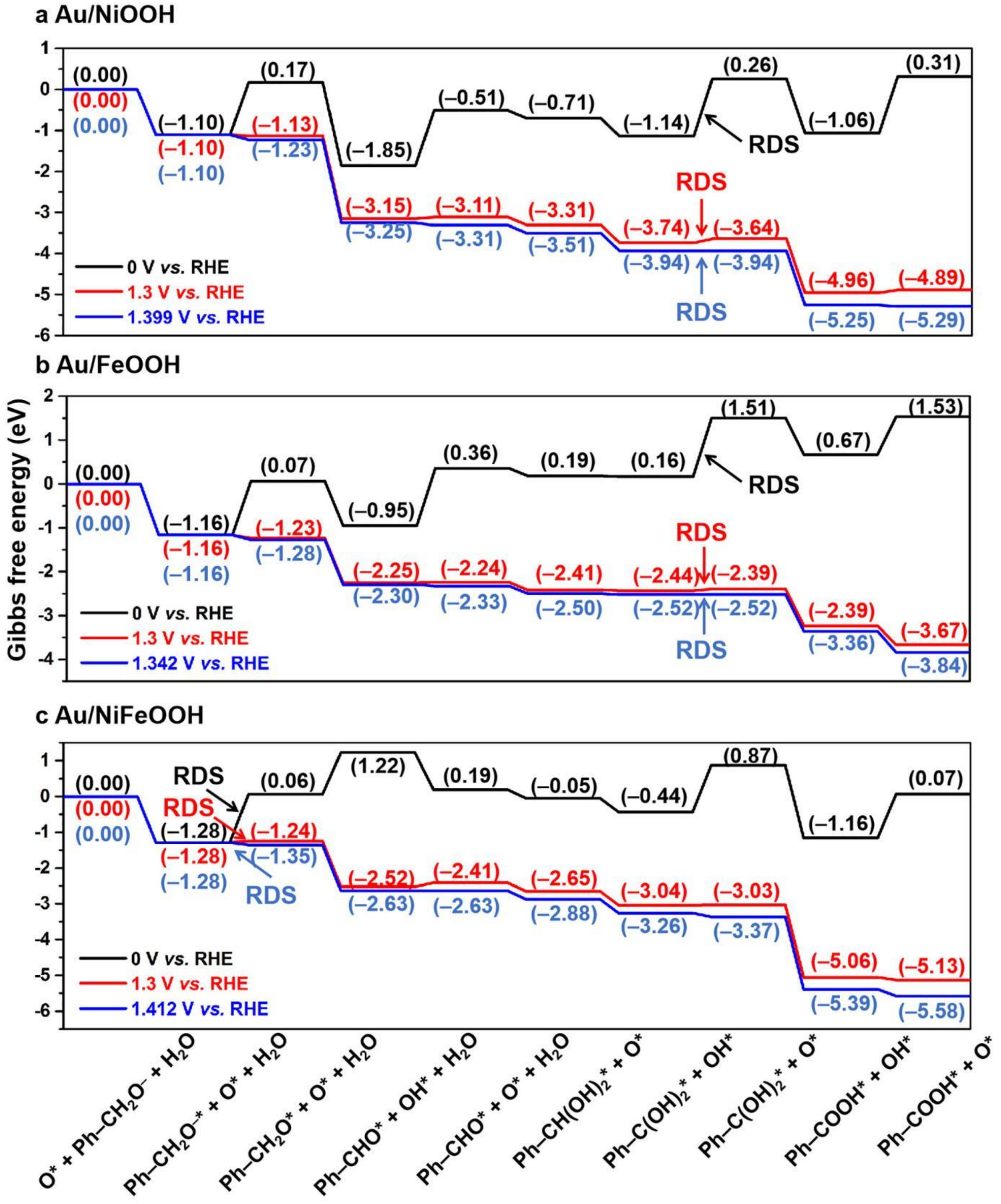

Supplementary Figure 32 | Gibbs free energy diagrams. Gibbs free energy diagrams for the benzyl alcohol oxidation to benzoic acid over Au/NiOOH (**a**), Au/FeOOH (**b**), and Au/NiFeOOH (**c**). The values of Gibbs free energies are labeled in the bracket in the unit of eV. The RDS for each electrocatalyst is labeled.

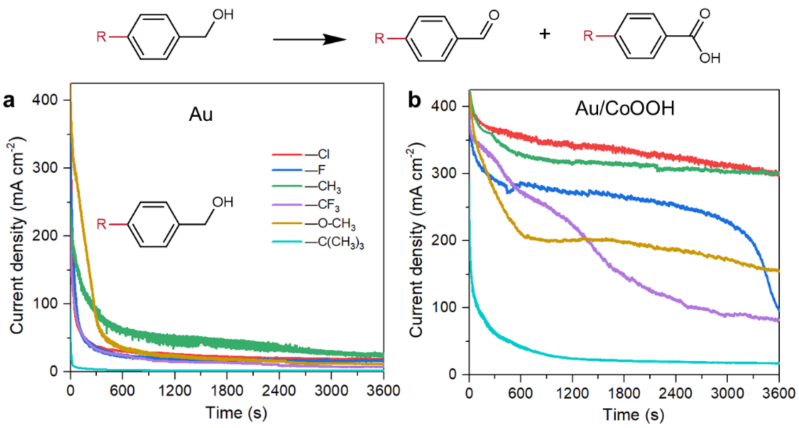

Supplementary Figure 33 | Electrochemical performances of benzyl alcohol derivatives. *I*-*t* curves of (**a**) Au and (**b**) Au/CoOOH catalyst for benzyl alcohol and its derivatives with different substituents (−Cl, −F, $-CH_3$, $-CF_3$, $-OCH_3$, $-C(CH_3)_3$) in 1 M KOH with 0.1 M reactant at 1.3 V *vs*. RHE at 60 ºC.

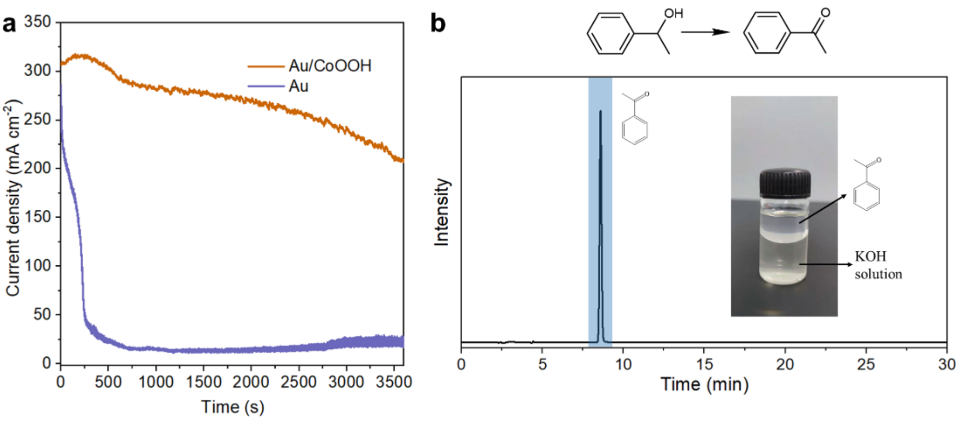

Supplementary Figure 34 | Electrochemical performances of 1-phenylethanol. a, *I-t* curves of Au/CoOOH and Au catalyst in 1 M KOH with 0.1 M *α*-phenethyl alcohol at 1.3 V *vs*. RHE at 60 ºC. **b**, HPLC spectrum of the oxidized products of *α*-phenethyl alcohol and the photograph of the obtained acetophenone produce (inset).

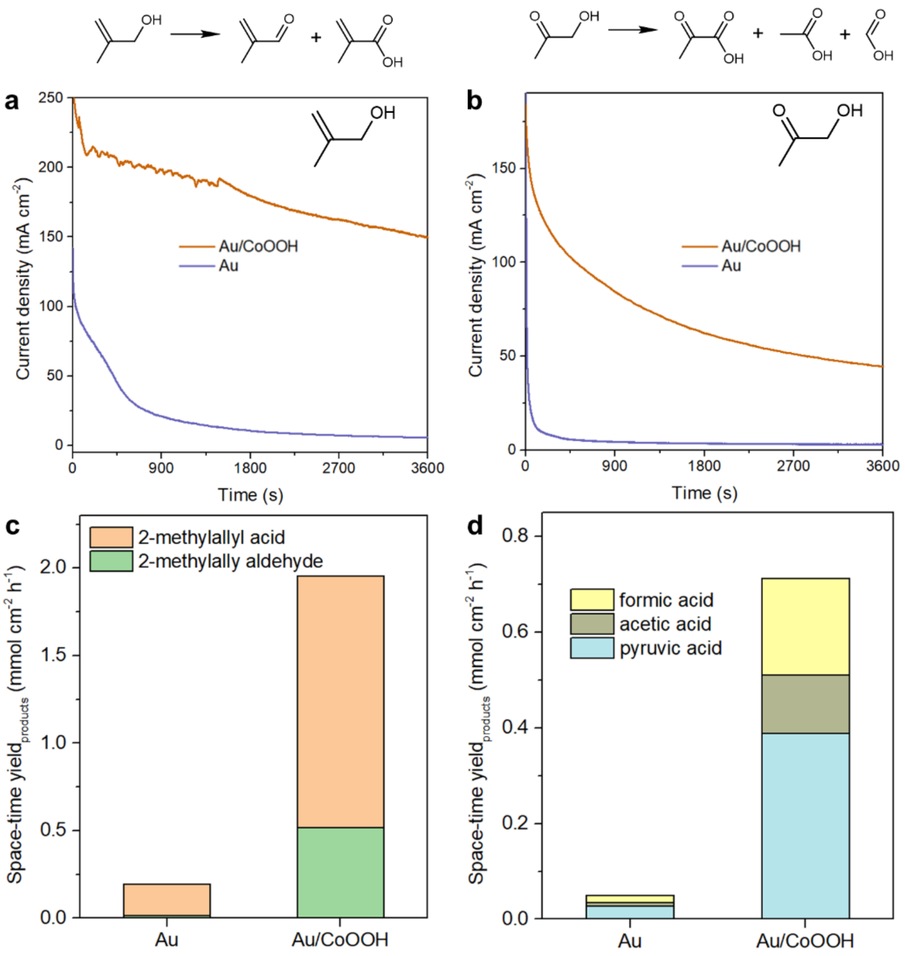

Supplementary Figure 35 | Electrochemical performances of alcohols with *α*–C=C&C=O. *I*-*t* curves of Au/CoOOH and Au catalyst for (**a**) methallyl alcohol and (**b**) hydroxyacetone in 1 M KOH with 0.3 M reactant at 1.3 V *vs*. RHE at r.t. The space-time yield of products for (**c**) methallyl alcohol and (**d**) hydroxyacetone.

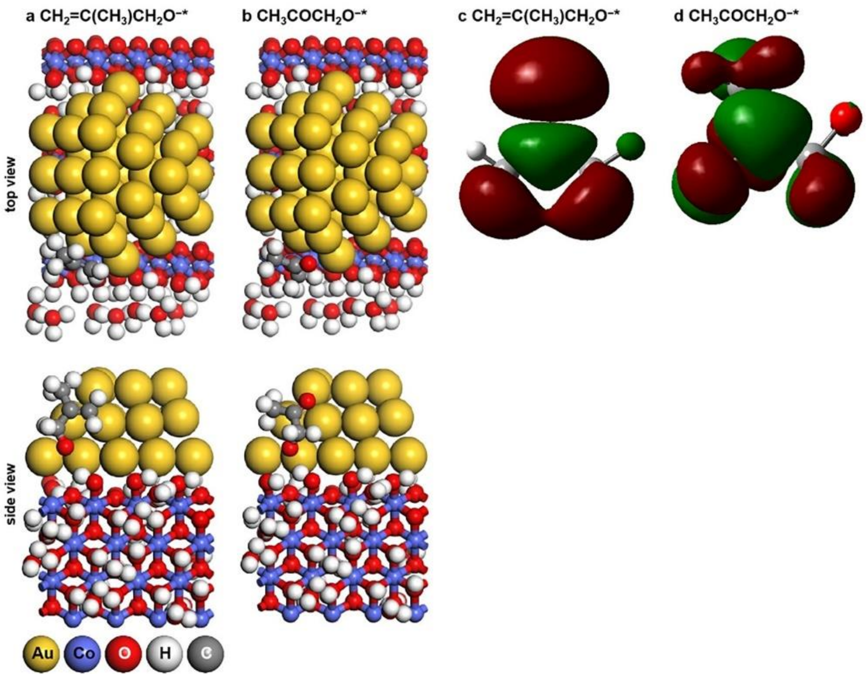

Supplementary Figure 36 | Adsorption of alcohols with *α*–C=C&C=O group. Top and side views of optimized geometries for $CH_2=C(CH_3)CH_2O^-*$ (* is Au/CoOOH) (**a**) and $CH_3COCH_2O^-*$ (**b**), LUMO of $CH_2=C(CH_3)CH_2O^-$ (**c**) and $CH_3COCH_2O^-$ (**d**).

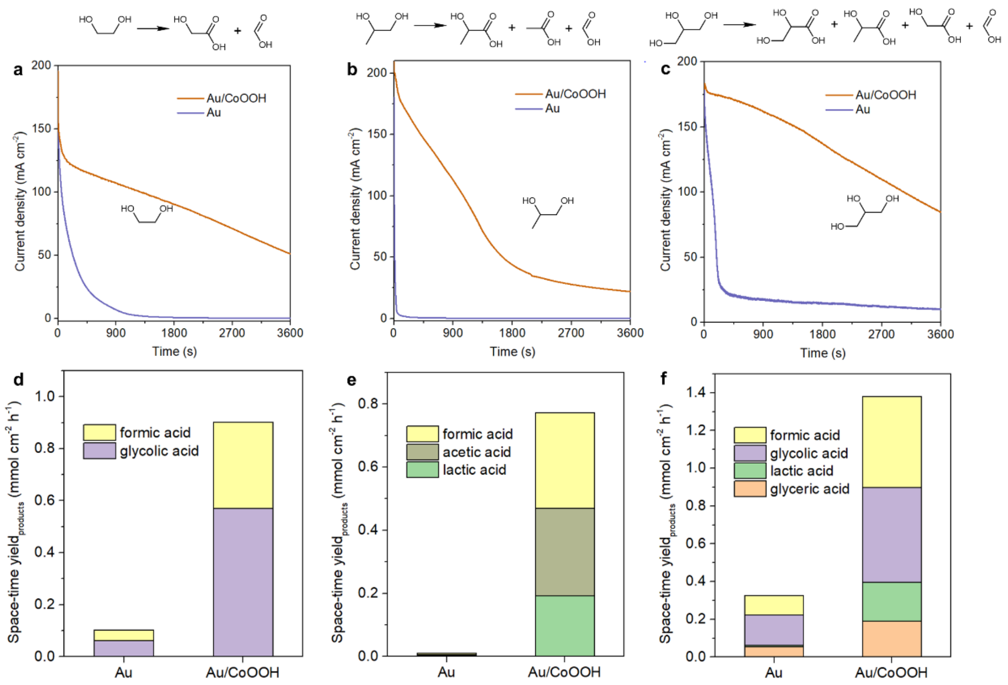

Supplementary Figure 37 | Electrochemical performances of polyols. *I-t* curves of Au/CoOOH and Au catalyst for (**a**) ethylene glycol, (**b**) 1,2-propanediol and (**c**) glycerol. The space-time yield of products for (**d**) ethylene glycol, (**e**) 1,2-propanediol and (**f**) glycerol.

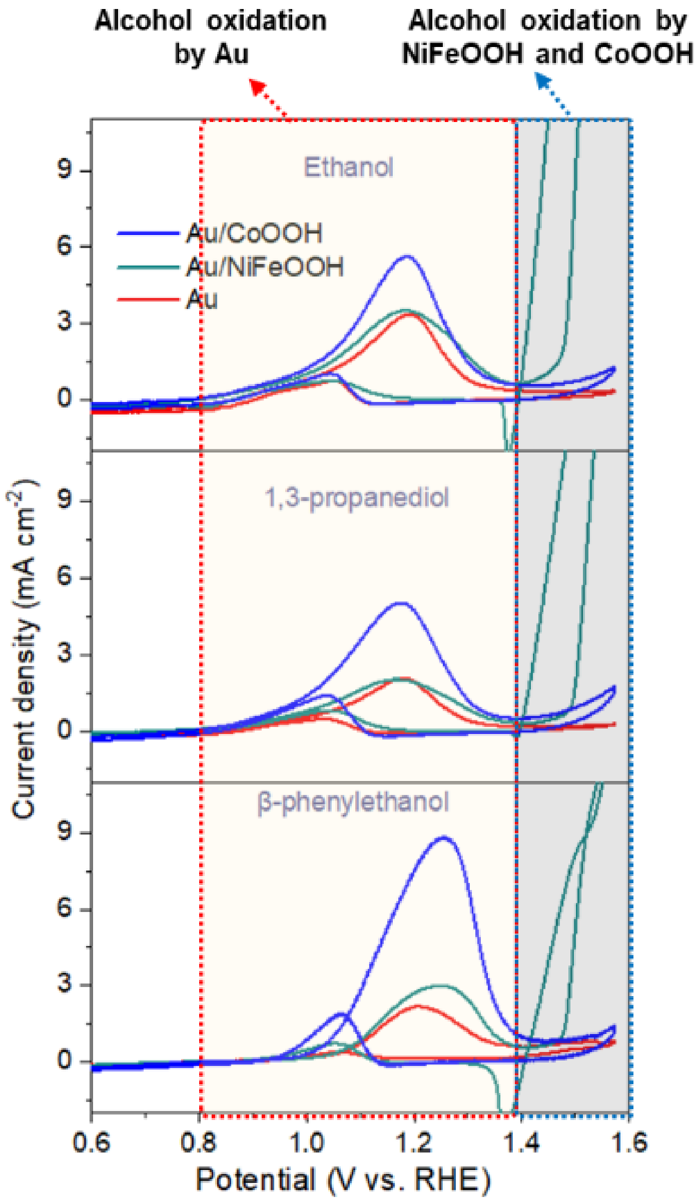

Supplementary Figure 38 | Electrochemical performances. CV curves in 1 M KOH with ethanol (0.3 M), 1,3-propanediol (0.1 M) or *β*-phenylethanol (0.1 M), respectively. Scan rate in all cases, 50 mV s^{-1}. Glassy carbon electrode was used as the conductive substrate for CV measurements.

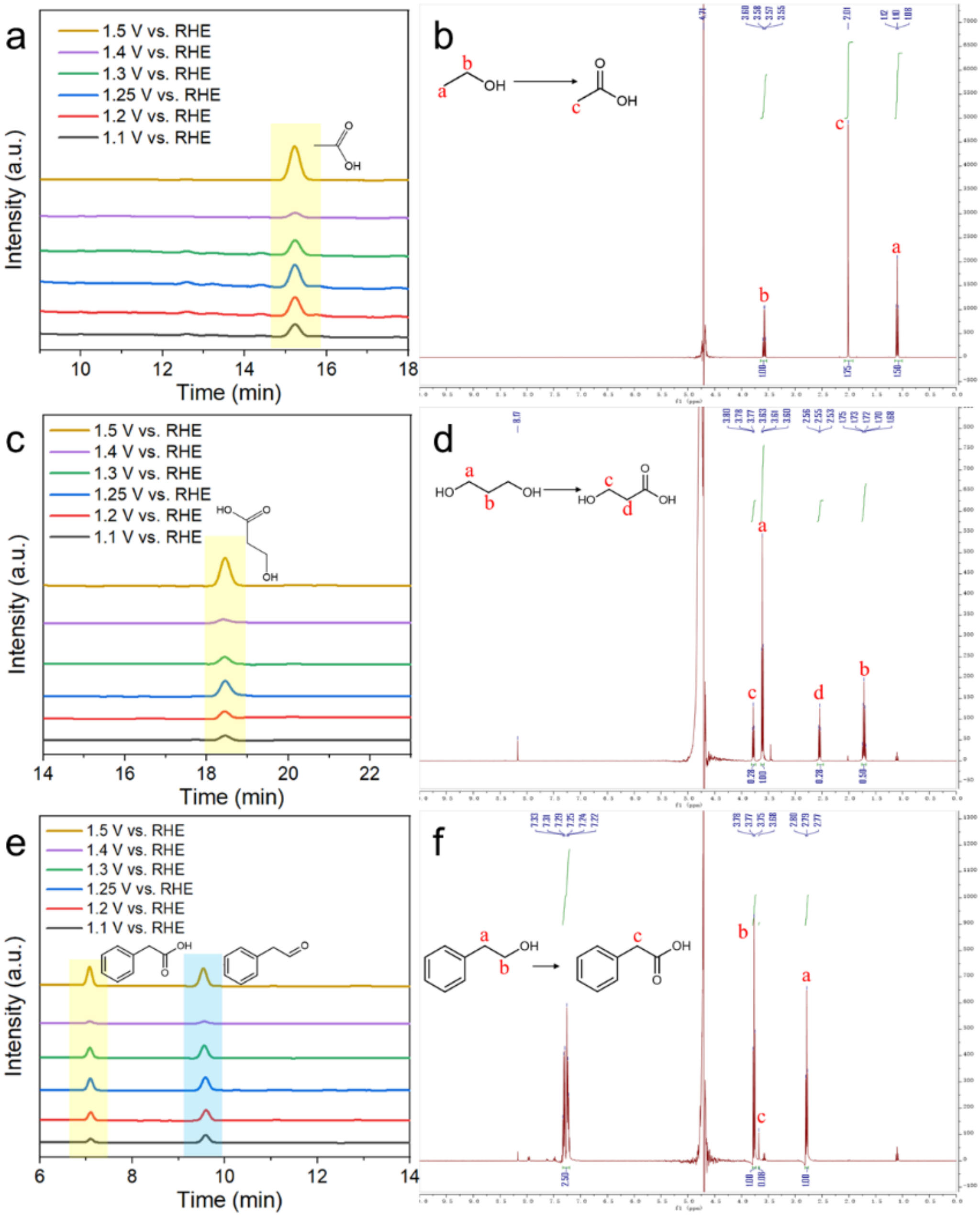

Supplementary Figure 39 | Products analysis. a,c,e, HPLC spectra of the oxidized products of (**a**) ethanol (**c**) 1,3-propanediol and (**e**) *β*-phenylethanol that oxidized by Au/CoOOH catalyst in1 M KOH with 0.1 M alcohols at different reaction potentials. **b,d,f,** the ^{1}H NMR spectra of the oxidized products of (**b**) ethanol (**d**) 1,3-propanediol and (**f**) *β*-phenylethanol.

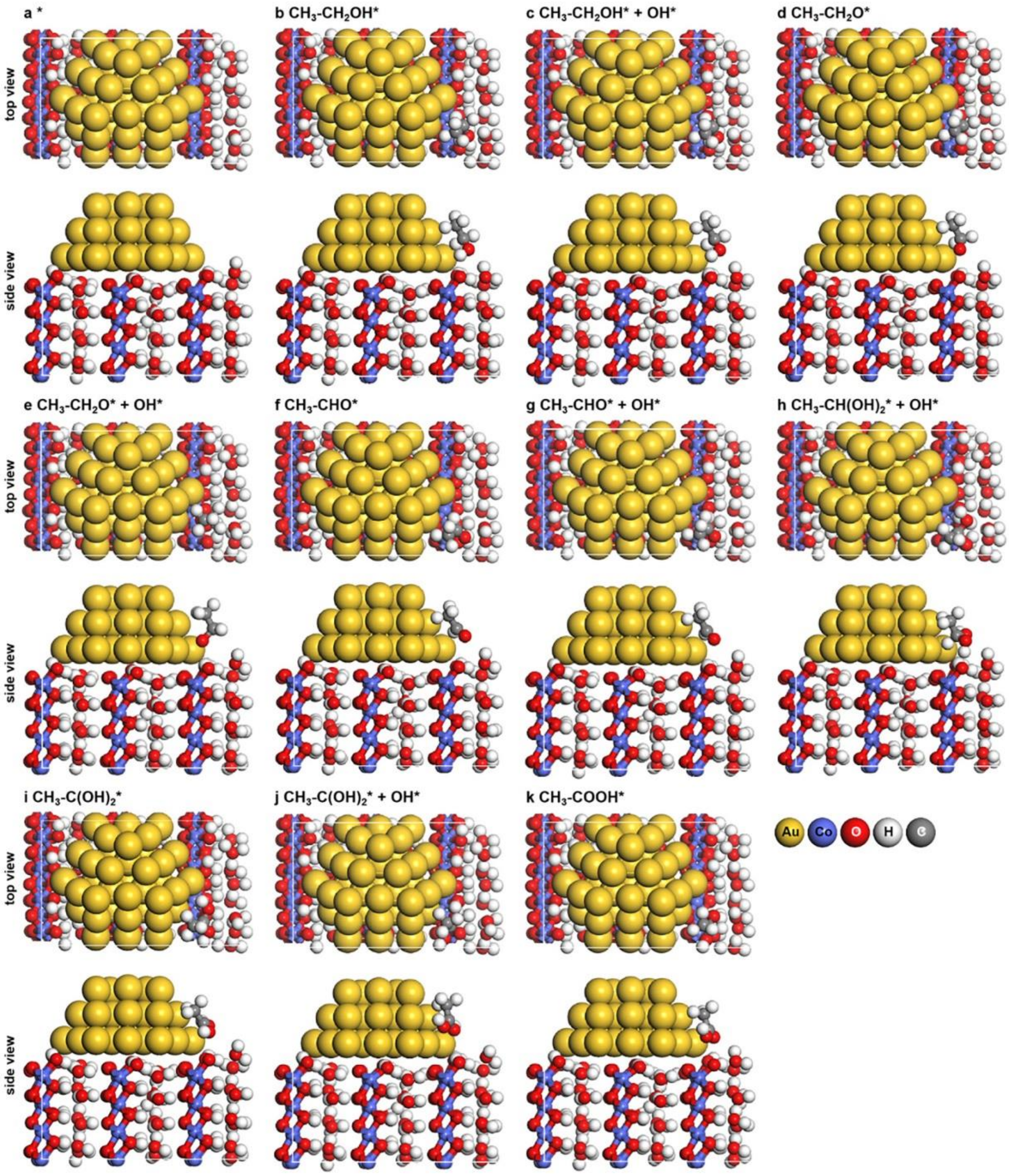

Supplementary Figure 40 | Optimized geometries for the reaction intermediates over Au/CoOOH. Optimized geometries for the reaction intermediates (* (**a**), CH_3–CH_2OH* (**b**), CH_3–CH_2OH* + OH* (**c**), CH_3–CH_2O* (**d**), CH_3–CH_2O* + OH* (**e**), CH_3–CHO* (**f**), CH_3–CHO* + OH* (**g**), CH_3–$CH(OH)_2$* + OH* (**h**), CH_3–$C(OH)_2$* (**i**), CH_3–$C(OH)_2$* + OH* (**j**),and CH_3–COOH* (**k**), respectively) in CH_3–CH_2OH oxidation to CH_3–COOH over Au/CoOOH. The color of each element is also labeled.

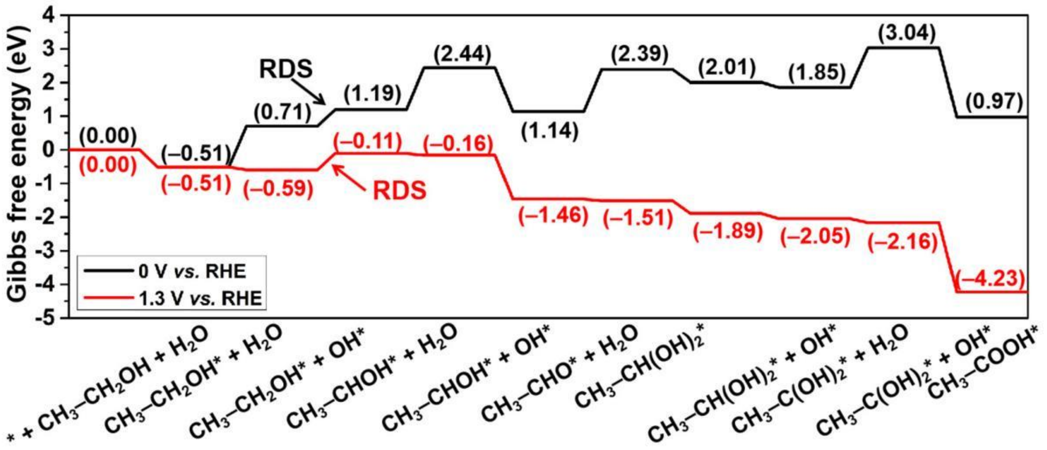

Supplementary Figure 41 | Gibbs free energy diagrams. Gibbs free energy diagrams for the oxidation of CH_3–CH_2OH over Au/CoOOH at 0 V and 1.3 V *vs.* RHE. The values of Gibbs free energies are labeled in the bracket in the unit of eV. The RDS is labeled.

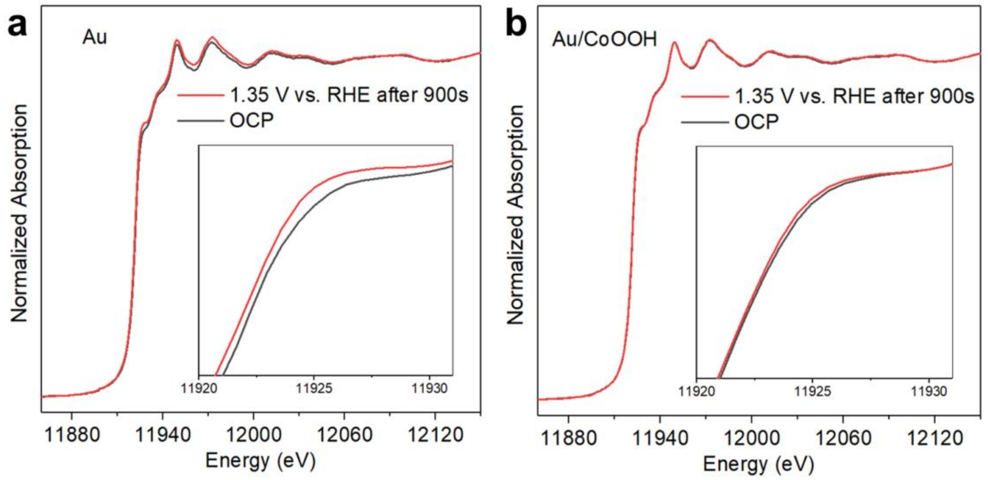

Supplementary Figure 42 | XANES spectra. Au L_3-edge XANES spectra of (**a**) Au and (**b**) Au/CoOOH at open circuit potential (OCP) and after 900 s at 1.35 V *vs*. RHE in a 1 M KOH with 0.1 M benzyl alcohol at r.t.

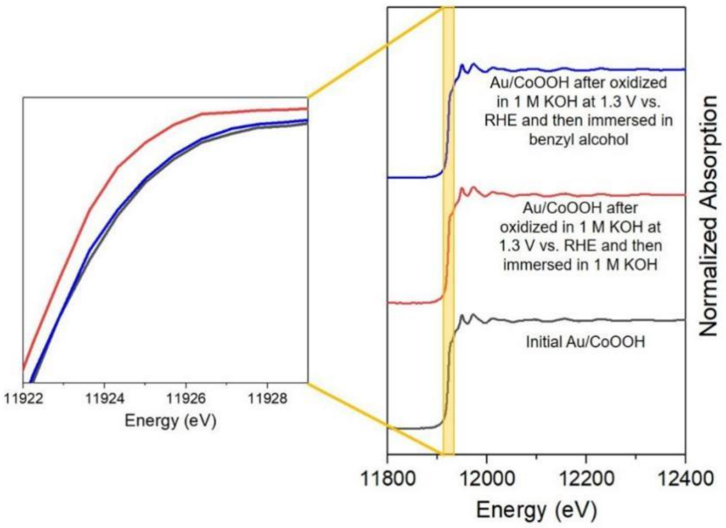

Supplementary Figure 43 | XANES spectra. Au L_3-edge XANES spectra of initial Au/CoOOH, Au/CoOOH being oxidized at 1.3 V vs. RHE in 1 M KOH and then immersed in KOH or in benzyl alcohol.

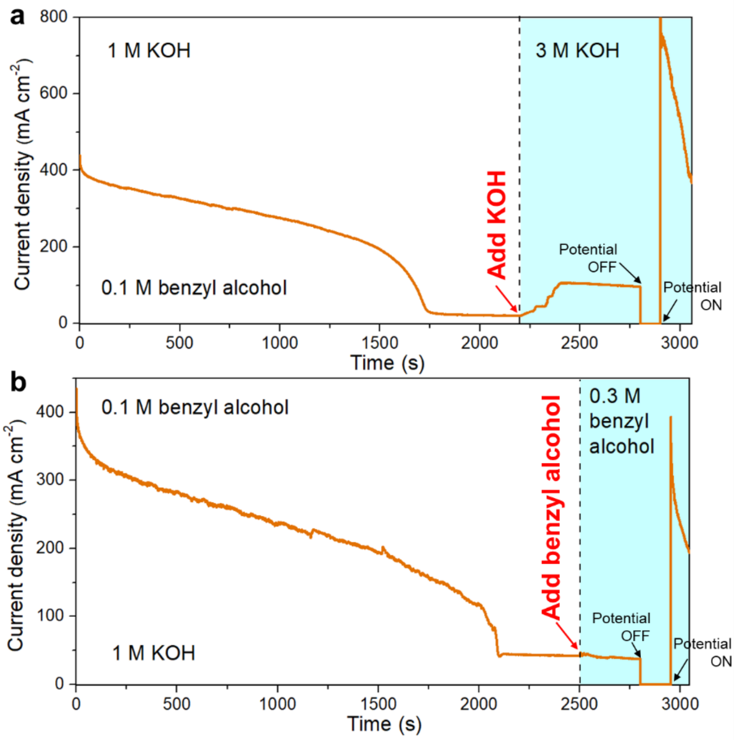

Supplementary Figure 44 | Decay and restoring of current density. a, *I-t* curve of Au/CoOOH at 1.3 V *vs*. RHE in 1 M KOH with 0.1 M benzyl alcohol at r.t. (KOH was added at 2200 s). **b**, *I-t* curve of Au/CoOOH at 1.3 V *vs*. RHE in 1 M KOH with 0.1 M benzyl alcohol at r.t. (benzyl alcohol was added at 2500 s).

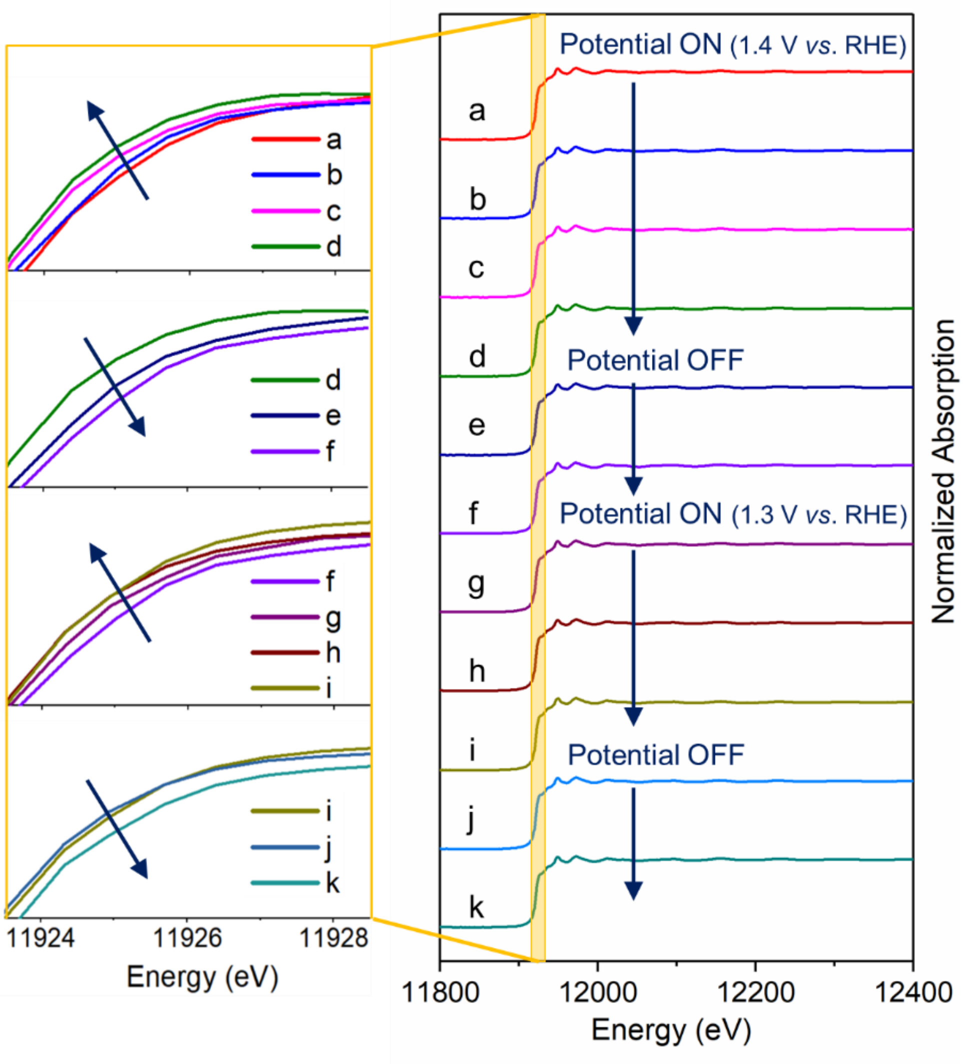

Supplementary Figure 45 | XANES spectra. The Au L_3-edge XANES spectra of Au/CoOOH when anodic potential (denoted as potential ON) and open circuit (denoted as potential OFF) were alternatively applied in a 1 M KOH with 0.1 M benzyl alcohol at r.t.. At potential ON (1.4 & 1.3V *vs*. RHE), the white line intensity was increased gradually (from line_**a** to line_**d** and line_**f** to line_**i**), which is assigned to Au oxidation. Subsequently, potential OFF was exerted and the white line intensity was decreased (from line_**d** to line_**f** and line_**i** to line_**k**), indicating that Au is reduced at open circuit.

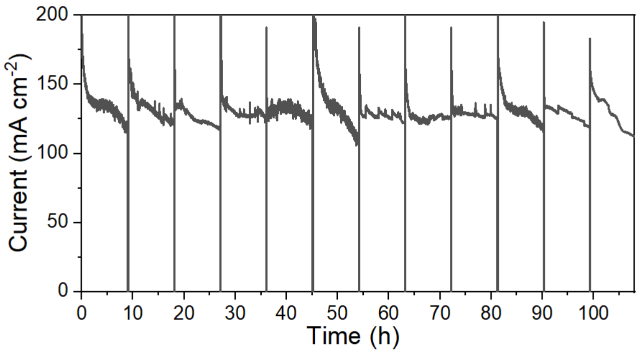

Supplementary Figure 46 | Long time stability test. Chronoamperometric measurements of Au/CoOOH catalyst using IP strategy at 1.2 V *vs*. RHE in a 1 M KOH with 0.1 M benzyl alcohol at r.t. over 108 h.

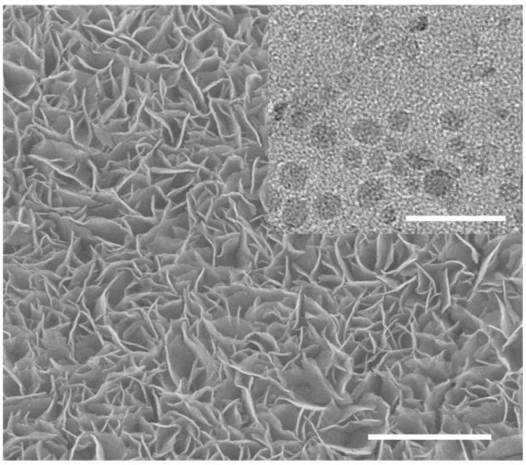

Supplementary Figure 47 | Characterisations of Au/CoOOH. SEM image of the Au/CoOOH catalyst after 24 h test. Scale bar, 2 μm. Inset displays TEM image of Au/CoOOH after 24 h test, showing the maintenance of the Au size. Scale bar, 20 nm.

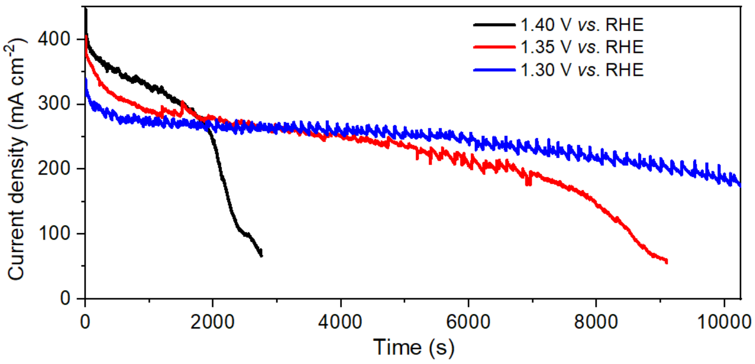

Supplementary Figure 48 | Electrochemical performances. *I-t* curves of Au/CoOOH in 1 M KOH with 0.1 M benzyl alcohol at different potentials.

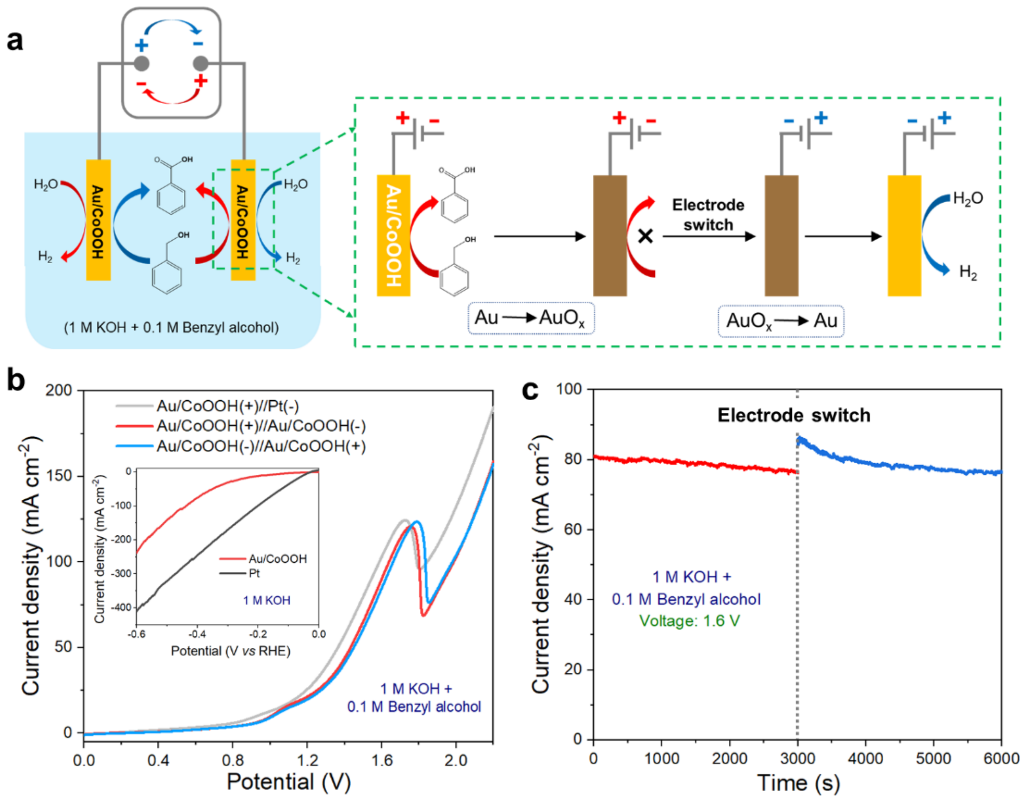

Supplementary Figure 49 | Symmetric single compartment (SSC) system. a, the schematic illustration of the SSC device by using Au/CoOOH as both anode and cathode for benzyl alcohol oxidation coupled with H_2 production. **b**, the LSV curves of Au/CoOOH in 1 M KOH with 0.1 M benzyl alcohol in the SSC system (inset: LSV curves of Au/CoOOH and Pt in 1 M KOH in a three-electrode system). **c**, *I-t* curves of Au/CoOOH in 1 M KOH with 0.1 M benzyl alcohol in the SSC system at voltage of 1.6 V, in which the electrodes were switched at 3000s.

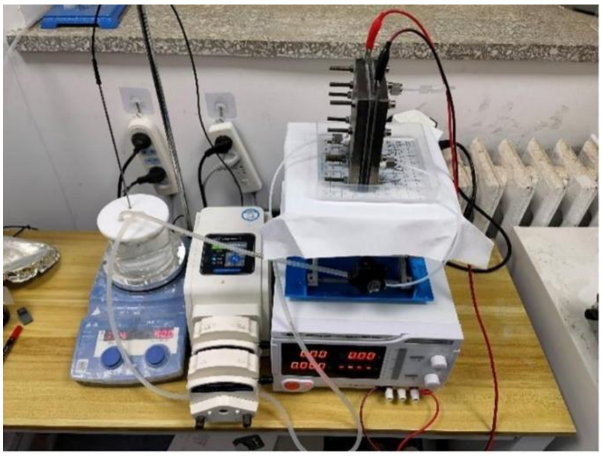

Supplementary Figure 50 | Two-electrode flow electrolyzer system. Photograph of the device for electrochemical tests in two-electrode membrane-free flow electrolyzer.

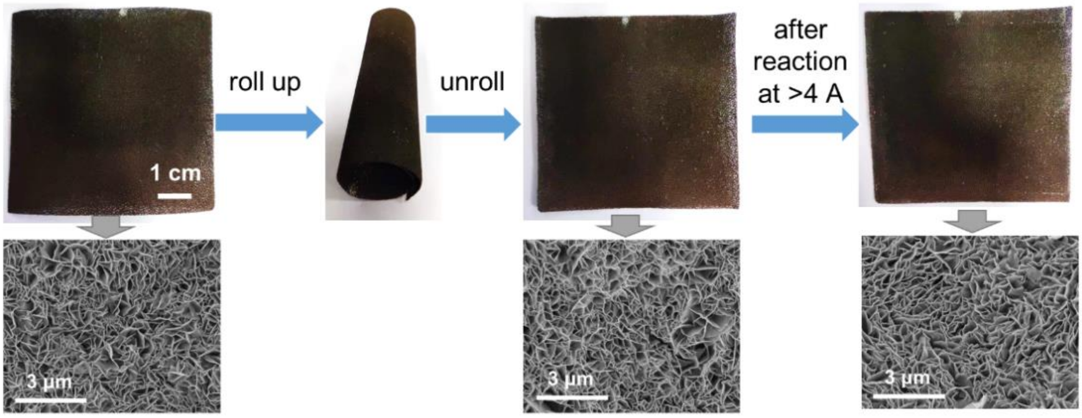

Supplementary Figure 51 | Catalyst mechanical stability evaluation. Photographs and the corresponding SEM images of Au/CoOOH catalyst on Ni foam.

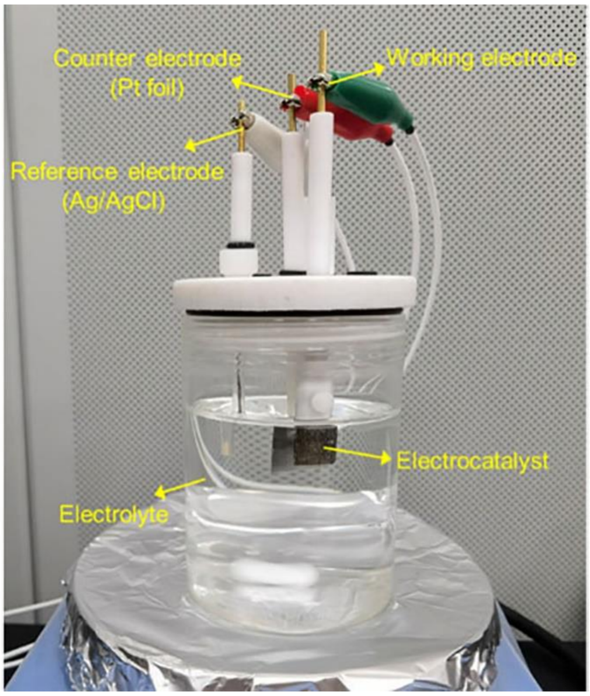

Supplementary Figure 52 | Three-electrode system. Photograph of the device for electrochemical tests in a three-electrode system.

Supplementary Tables

Supplementary Table 1. Comparison of the catalytic performance of anodic oxidation reactions over Au-based catalysts reported in the literatures and in this work.

**Catalysts**	**Reactant**	**Conductive substrate**	**Electrolyte**	**Loading mass (cm^{-2})**	**Scan rate of LSV ($mV\ s^{-1}$)**	**Potential (V *vs.* RHE)**	**Current density ($mA\ cm^{-2}$)**	**Ref.**
Au/C	glycerol (0.1 M)	carbon cloth	0.1 M KOH (60 °C)	1 mg_{Au}	1	1.5	45	*1*
AuPt/C	glycerol (0.5 M)	graphite paper	2 M KOH	0.24 mg	50	1.2	120	*2*
Pt-modified Au	Ethylene glycol (0.1 M)	Au electrode	0.5 M NaOH	-	50	0.92	40	*3*
PtAu/Au	Ethanol (1 M)	glassy carbon electrode	1 M KOH	0.07 mg	50	0.92	36	*4*
AuPd@Pd	Ethanol (0.5M)	-	0.5 M KOH	-	50	1.05	91	*5*
Au@FS-rGO	Sorbitol (0.5 M)	glass carbon electrode	0.5 M KOH	0.25 mg	50	1.35	17	*6*
Au@PdAg NSs/rGO	Ethanol (1 M)	glass carbon electrode	1 M KOH	-	50	0.9	6.5	*7*
AuPt/Ni	Glycerol (1 M)	Ni foil	0.1 M NaOH	-	50	1.9	14	*8*
PtAu/C	Glycerol (1 M)	graphite rod	1 M KOH	0.1 mg	10	0.97	95	*9*
Au_3Ag/C	Glycerol (1 M)	carbon cloth	4 M KOH (90 °C)	2 mg	10	1.5	330	*10*
PdAuRu	Ethylene glycol (1 M)	glass carbon electrode	1 M KOH	-	50	0.97	22	*11*
Nanoporous gold	Methanol (1 M)	glass carbon electrode	0.5 M KOH (photoelectric)	-	10	1.25	0.6	*12*
Au/CC	Glycerol (0.5 M)	glass carbon electrode	0.3 M NaOH	0.5 mg	5	1.3	85.6	*13*
Pd_2Au_1/C	HMF (0.02 M)	glass carbon electrode	0.1 M KOH	0.1 mg	50	0.97	7	*14*
hollow Au@Pd	Ethanol (0.5M)	glass carbon electrode	1 M NaOH	-	50	0.7	49	*15*
$Au_{55}Cu_{25}Si_{20}$	Ethanol (2 M)	Au-based metallic glass ribbon	0.5 M KOH	-	50	1.4	0.928	*16*
Au/C	Ethylene glycol (0.5 M)	glassy carbon electrode	1 M KOH	0.41 mg	-	1.3	15	*17*
Au disk	Ethylene glycol (1 M)	Au disk electrodes	0.1 M KOH	-	50	1.55	32	*18*
Au/Ti	Ethanol (1 M)	Ti foil	1 M KOH	1.9 mg	10	2	2.5	*19*
AuCu	Ethylene glycol (1 M)	glassy carbon electrode	1 M KOH	0.06 mg_{Au}	10	0.97	93	*20*

Au-Pd alloy nanoparticles	Ethanol (0.1 M)	ITO electrodes	0.1 M KOH	0.1 mg	50	1.1	4.3	*21*
AuPd	Methanol (1 M)	glassy carbon electrode	1 M NaOH	0.1 mg	50	0.84	62	*22*
Au/CoOOH	benzyl alcohol (0.1M)	nickel foam	1 M KOH	0.17 mg_{Au}	10	1.3<br>1.4<br>1.5	340<br>455<br>540	*This work*

Supplementary Table 2. Comparison of the catalytic performance of anodic oxidation reactions reported in the literatures and in this work.

Catalysts	Reactant	Conductive substrate	Electrolyte	Loading mass (mg cm^{-2})	Scan rate of LSV (mV s^{-1})	Potential (V *vs.* RHE)	Current density (mA cm^{-2})	Ref.*
NC@$CuCo_2N_x$/CF	benzyl alcohol (10 mM)	carbon fiber	1 M KOH	2	5	1.4	125	*8*
Ni-Mo-N/CFC	glycerol (0.1 M)	carbon fiber cloth	1 M KOH	2.9	10	1.88	300	*9*
Ni_2P	tetrahydroisoquinoline (0.5 mmol)	nickel foam	1 M KOH	-	5	1.57	180	*13*
Co_3O_4 NWs/Ti	benzyl alcohol (10 mM)	Ti membrance	0.1 M NaOH	-	10	2.598	9	*14*
A-Ni-Co-H	benzyl alcohol (0.1M)	nickel foam	1 M KOH	-	10	1.45	400	*15*
$CuCo_2O_4$	HMF (50 mM)	glass carbon electrode	1 M KOH	-	5	1.5	220	*27*
$NiSe_2$-NiO	Urea (0.33 M)	glass carbon electrode	1 M KOH	0.4	10	1.5	150	*28*
NiFe-LDH	HMF (100 mM)	carbon fiber paper	1 M KOH	-	5	1.5	300	*29*
Nanocrystalline Cu foam	HMF (5 mM)	Cu foam	0.1 M KOH	-	10	1.9	8	*30*
Ni_3N@C	HMF (10 mM)	nickel foam	1 M KOH	-	5	1.68	250	*31*
M-$Ni(OH)_2$	Urea (0.33 M)	glass carbon electrode	1 M KOH	0.5	50	2.023	50	*32*
Au/CoOOH	benzyl alcohol (0.1M)	nickel foam	1 M KOH	1.6	10	1.3<br>1.4<br>1.5	340<br>455<br>540	*This work*

Note. *The references were cited in the manuscript.

Supplementary Table 3. The mass loading of Au and CoOOH on Ni foam for Au/CoOOH and Au catalysts by inductively coupled plasma mass spectrometry (ICP-MS).

Catalysts	Mass loading of Au ($mg\ cm^{-2}$)	Mass loading of CoOOH ($mg\ cm^{-2}$)	Au mass loading on CoOOH (wt%)
Au/CoOOH	0.17	1.5	11.3
Au	0.33	0	-

Supplementary Table 4. Gibbs free energy changes (ΔG) of the generation of OH* and O* with different coordination numbers on CoOOH, NiOOH, FeOOH, and NiFeOOH at 0 V *vs.* RHE

electrophilic species	ΔG (eV)			
	CoOOH	NiOOH	FeOOH	NiFeOOH
μ_1–OH	1.248	1.643	1.682	1.656
μ_2–OH	0.554	0.484	–0.934	–0.298
μ_3–OH	–0.095	0.338	–0.877	–0.710
μ_1–O	2.331	2.329	2.510	3.012
μ_2–O	1.443	1.388	1.354	1.411
μ_3–O	1.073	0.902	0.386	0.904

Supplementary Table 5. The electric energy consumptions *via* IP and CP strategies.

	I ($A\ cm^{-2}$)	t (h)	$U \times t$ (V·h)[a]	W_E ($Wh\ cm^{-2}$)
IP strategy	0.2	0.33	0.406	0.081
	0.3	0.33	0.453	0.136
CP strategy	0.2	0.33	0.565	0.112
	0.3	0.33	0.594	0.178

Note: [a]$U \times t$ is directly obtained based on the integral area of the U-t curves in Fig. 6c

Supplementary Notes

Supplementary Note 1

The mechanism of oxygen evolution reaction on CoOOH was calculated. The oxygen evolution reaction on CoOOH happens in four consecutive steps:

$$* + OH^- \rightarrow OH^* + e^-, \quad (A)$$

$OH^* + OH^- \rightarrow O^* + H_2O + e^-$, (B)

$O^* + OH^- \rightarrow OOH^* + e^-$, (C)

$OOH^* + OH^- \rightarrow * + O_2 + H_2O + e^-$. (D)

The optimized geometries of the reaction intermediates in oxygen evolution reaction are displayed in Supplementary Fig. 7 and the obtained Gibbs free energy diagram of oxygen evolution reaction on CoOOH is shown in Supplementary Fig. 8. The ΔG of these elementary steps are calculated to be 0.554 eV, 1.433 eV, 1.558 eV, and 1.399 eV, respectively. The RDS is the generation of OOH* with a ΔG of 1.558 eV at 0 V *vs.* RHE. Thus the oxygen evolution reaction on CoOOH occurs at potential larger than 1.558 V *vs.* RHE.

Supplementary Note 2

To address the economic issue of acid neutralization of the carboxylate products, the development of high-performance catalysts working in acidic or neutral electrolyte systems is important. In addition, improving the economic value of alcohol oxidation coupling H_2 production process in alkaline electrolyte would also be a promising solution. The following two aspects can be considered in this work: *i*) The first aspect is to increase the product value. For example, the electrooxidation of ethylene glycol produces glycolic acid with FE of 65 % (Supplementary Fig. 37). Glycolic acid is widely used as monomer to produce biodegradable polyglycolic acid (PGA) with price of 100,000–300,000 \$/tons, while the price of ethylene glycol is <1000 \$/tons[23]. The high value of the product can effectively offset the cost of the acid neutralization process, making the reaction ecumenically feasible. Although the selectivity of glycolic acid is still unsatisfactory in the current work, we believe the performance can be improved in the future by designing more active electrocatalysts; *ii*) Secondly, for some alcohol oxidation, the product is insoluble in water which can be easily separated from the electrolyte. As shown in Supplementary Fig. 34, for the oxidation of α-phenethyl alcohol over the Au/CoOOH catalyst, acetophenone is the only oxidation product as confirmed by HPLC. As acetophenone is insoluble in water, it spontaneously floats on top of the electrolyte and can be easily abstracted (*see* the photograph in Supplementary Fig. 34b (insert)).

Supplementary Note 3

It is an essential issue to control the selectivity of the products (such as benzaldehyde,

benzoic acid). In terms of benzaldehyde, we found that its selectivity is sensitive to the pH of electrolyte. High selectivity of benzaldehyde can be obtained by reducing the pH of electrolyte. For example, the selectivity of benzaldehyde was increased from 52.2% at pH = 14 to 92.5% at pH = 9 with other reaction parameters were kept the same. The conversion rate of benzyl alcohol decreases with the decrease of pH, which may be due to the formation of fewer OH* on the catalyst surface under weak alkaline conditions (Supplementary Fig. 10).

From the electronic or crystal structures point of view as the reviewer mentioned, the control of product selectivity can be envisaged by regulation of intermediate adsorption strength (for example, benzaldehyde in this work) by engineering the electronic and geometric structures of the catalyst. A relatively weak adsorption of benzaldehyde on the catalyst surface may facilitate its desorption, leading to its high selectivity. On the contrary, a stronger adsorption of benzaldehyde would promote its further oxidation to benzoic acid. For example, the vacancies or defects on the catalyst surface can enhance the adsorption of carbonyl groups of benzaldehyde, which may promote the formation of benzoic acid[24]. In contrast, the catalysts with hydrophilic surfaces may not be conducive to the adsorption of carbonyl group, and thus high selectivity of benzaldehyde can be obtained[25].

Supplementary Note 4

we have calculated the turnover frequency (TOF) of Au and Au/MOOH catalysts based on the following equations:

$$\mathrm{TOF} = \frac{\text{total benzyl alcohol turnovers } (\mathrm{cm^2}_{\mathrm{geo}})}{\text{total active sites of Au } (\mathrm{cm^2}_{\mathrm{geo}})}$$

In the equation, the total benzyl alcohol turnovers were obtained based on the HPLC results after reaction at constant potentials, the total active sites of Au were calculated based on the ECSA results (Supplementary Fig. 13), which was determined by underpotential deposition of Cu (Cu-UPD) method[26,27].

We first compared the TOF values of Au and Au/CoOOH at different potentials. Before 1.2 V *vs*. RHE, (Supplementary Fig. 14c), the TOF values of Au/CoOOH are only slightly higher than that of pure Au at potentials of 1.0 and 1.1 V *vs*. RHE, indicating the intrinsic activity of pure Au is close to Au in Au/CoOOH. The slightly high activity of Au/CoOOH may be due to the enhanced adsorption of benzyl alcohol on Au/CoOOH interface that promotes the oxidation of benzyl alcohol. At 1.2 V vs. RHE, the TOF value of pure Au only shows slight

enhancement compared with that at 1.1 V vs. RHE, indicating the intrinsic activity of Au is largely remained, with the formation of more OH* species on Au surface with higher activity. In contrast, the TOF value of Au/CoOOH increases sharply at 1.2 V vs. RHE. As the intrinsic activity of Au in Au/CoOOH is close to pure Au, we postulate the significantly higher TOF value of Au/CoOOH is contributed by CoOOH. We have proven that OH* is generated over CoOOH at potential ~1.2 V *vs.* RHE, which is capable of oxidizing benzyl alcohol adsorbed on Au/CoOOH interface, in turn enhancing the current density with higher TOF. At 1.3 V vs. RHE, the TOF of Au decreases due to the formation of AuO_x. In comparison, the TOF of Au/CoOOH increases, which we speculate is due to the enhanced adsorption of benzyl alcohol at Au/CoOOH interface that delays Au deactivation process. This is supported by the LSV and CV curves of Au/CoOOH for benzyl alcohol oxidation in Fig. 2a and 2c.

To study the difference of the current density over different Au/MOOH catalysts, we examined the exposed number by measuring ECSA and the TOF of Au on these catalysts. As shown in Supplementary Figs. 13 and 14d-f, Au/MOOH sample exhibits higher ECSA than pure Au, indicating the nanosheet array structure of Au/MOOH can promote the exposure of Au active sites. We calculated the TOF based on the equations shown above. The results show that the normalized TOF are very close for Au/FeOOH, Au/NiOOH and Au/NiFeOOH at 1.0 V *vs*. RHE (Supplementary Fig. 14e). Therefore, we conclude that there is no obvious difference for the intrinsic activity of Au/MOOH samples. In addition, the slightly high activity of Au/MOOH than pure Au may be due to the enhanced adsorption of benzyl alcohol on Au/MOOH interface that facilitates benzyl alcohol oxidation. The current densities tend to decay at higher potential, which is due to the formation of AuO_x.

Supplementary Note 5

The model of bulk γ-CoOOH was constructed based on the following experimental results: the XRD measurement of Au/CoOOH reveals a γ-CoOOH phase with typical (003) and (006) reflections at 2θ of 12.9° and 26.2° (Fig. 3d). HRTEM image of CoOOH displays the ultrathin nanosheet structure with the interplanar spacing of 0.73 nm and 0.24 nm, corresponding to the (001) and (100) crystal plane of γ-CoOOH (Supplementary Fig. 15). Therefore the model of bulk γ-CoOOH is constructed with the space group of $R\bar{3}mH$ at first. The lattice parameters of bulk γ-CoOOH are $a = b = 2.851$ Å, $c = 13.15$ Å, $\alpha = \beta = 90°$, and $\gamma =$

120°.

The model of bulk Au is constructed with the space group of $Fm\bar{3}m$. The lattice parameters of bulk Au are $a = b = c = 4.07$ Å, $\alpha = \beta = \gamma = 90°$. The (111) facet have been reported to be the preferably exposed facet of bulk Au in previous literatures[28,29]. The XRD measurement of Au/CoOOH also reveals a reflection peak at $2\theta = 38.2°$, which is attributed to the Au (111) reflection (Fig. 3d). Thus, the main exposed crystal face of Au particles should be the (111) facet. The model of Au was constructed by cleaving a close-packed Au cluster from the (111) facet of bulk Au. The simplified model of Au is a Au_{37} cluster containing 19, 12, and 6 Au atoms in the bottom, middle, and uppermost layers, as displayed in Supplementary Fig. 18. The exposed facets of Au_{37} is the (111) facet and (100) facet, matching with HRTEM image of Au/CoOOH (Supplementary Fig. 15 and Fig. 3b,c in the manuscript).

The exposed surface of γ-CoOOH for supporting Au nanoparticles (NPs) is constructed based on the following experimental results: The HRTEM images reveal that Au nanoparticles are randomly distributed on the surface ((001) facet) and edge ((100) and (110) facets) of γ-CoOOH nanosheet with an average diameter of 4.3 nm (Supplementary Fig. 15 and Figs. 3b,c in the manuscript). In previous literature, the (110) facet has been determined to be the active site of oxyhydroxy[30]. Thus the (001) and (110) facets of γ-CoOOH are constructed. The coordination environments of surface oxygen species OH* on the (100) and (110) facets of γ-CoOOH are the same, namely, half of the OH* is one-coordinated (μ_1-OH), and another half is two-coordinated (μ_2-OH).

To determine the reaction active site on γ-CoOOH for oxidizing the benzyl alcohol, we calculated the generation ability of OH* and O* over the (001) and (110) facets of CoOOH. The electrophilic OH* and O* may exist in six forms, OH* coordinated with one Co atom (μ_1–OH), two Co atoms (μ_2–OH), or three Co atoms (μ_3–OH), and O* coordinated with one Co atom (μ_1–O), two Co atoms (μ_2–O), or three Co atoms (μ_3–O), respectively. The μ_3–OH and μ_3–O can be generated on the (001) facet of CoOOH while μ_1–OH, μ_2–OH, μ_1-O, and μ_2–O can be generated on the (110) facet. The Gibbs free energy changes (ΔG) of the generation of μ_1-OH, μ_2-OH, μ_3-OH, μ_1-O, μ_2-OH, and μ_3-OH are calculated to be 1.248, 0.554, –0.095, 1.073, 1.443, and 2.331 eV at 0 V *vs.* RHE, respectively (Supplementary Table 4), corresponding to the onset potentials of 1.248, 0.554, –0.095, 1.073, 1.443, and 2.331 V *vs.* RHE, respectively. According to the LSV curves (Fig. 2a-2d in manuscript), the onset potential

is about 1.2 V *vs.* RHE, therefore the reaction active electrophilic species of CoOOH can be estimated to be the μ_1–OH, which can be generated on the (110) facet of γ-CoOOH. Thus the model of CoOOH is constructed by cleaving the (110) facet of γ-CoOOH (Supplementary Fig. 18a). Thus, the model of Au/CoOOH for benzyl alcohol oxidation in our work was constructed by placing the Au_{37} on the (110) facet of CoOOH. The chemical formula of model Au/CoOOH is $Au_{37}Co_{48}O_{119}H_{95}$ with both the CoOOH (110) facet and Au (111) facet exposed (Supplementary Fig. 18c).

Supplementary Note 6

A scheme has been supplemented to provide a clear physics image as suggested. According to the optimized geometry of Ph–CH_2O^{-}* for Au/CoOOH (Supplementary Fig. 20a), the benzyl alcohol (in the form of alkoxide) is adsorbed with its phenyl group paralleled to the (111) facet of Au. The distance between the phenyl group of benzyl alkoxide and Au is denoted as d_π, and was calculated to be 3.03 Å. The O atom in –CH_2O^{-} group of benzyl alkoxide is adsorbed on Au with a Au–O bond. The bond length of this Au–O bond is denoted as d_σ, and was calculated to be 2.18 Å. Referred to the previous literature[31], it can be deduced that a σ–π interaction exists between benzyl alkoxide and Au at Au/CoOOH interface, *i.e.*, the σ bond between the lone pair electrons of O atom in –CH_2O^{-} and Au 6*s* orbital, and d–π bond between the π* orbital of benzyl alkoxide and Au 5*d* orbitals. For the adsorption of benzyl alkoxide on Au, the d_π and d_σ are calculated to be 3.27 Å and 2.36 Å, respectively. Both d_π and d_σ for Au are larger than those for Au/CoOOH, indicating that the σ–π interaction between benzyl alkoxide and Au is weaker than that between benzyl alkoxide and Au/CoOOH. This result is due to the electron transfer from Au to CoOOH, which makes the Au atoms at Au/CoOOH interface to be electron deficient (Supplementary Fig. 20b).

Hirshfeld charge analysis was performed on Au/CoOOH and Ph-CH_2O−* (* is Au/CoOOH) (Supplementary Fig. 20b). It is found that after combining with CoOOH, 2.57 *e* of electron is transferred from Au to CoOOH, which makes the Au to be positively charged, especially the Au atoms adjacent to CoOOH. The average Hirshfeld charge of Au atoms in the bottom layer of Au is 0.14 *e*, while those of middle layer and top layer are 0.00 *e*, and −0.01 *e*, respectively. After adsorption of Ph-CH_2O^{-}, 0.69 *e* of electron is transferred from Ph-CH_2O^{-} to Au/CoOOH through Au-O interaction. Furthermore, the bond order of Au-O bond is calculated

to be 0.54, indicating that the strength of Au-O interaction is in the magnitude of covalent bond. On the other hand, the density of states for Au/CoOOH, together with the frontier orbitals of Ph-CH_2O^- were calculated (Fig. 4d). It is found that the *d* orbital of Au (ranging from −7.91 to −3.76 eV *vs.* vacuum level) overlaps with the LUMO of Ph-CH_2O^- (−3.91 eV *vs.* vacuum level), suggesting that there exists a feedback *d-π* interaction between Au and Ph-CH_2O^-, which reinforces the adsorption of Ph-CH_2O^- on Au/CoOOH.

Supplementary Note 7

We compared the adsorption configurations of benzyl alcohol on Au and Au/CoOOH and redrew the schematic diagram for the benzyl alcohol oxidation over Au, CoOOH and Au/CoOOH:

For Au, DFT calculation results show that benzyl alcohol (in the form of alkoxide) was adsorbed on Au by forming a σ bond between Au 6*s* orbital and the lone pair electron of the −CH_2O^- group of alkoxide, and a π bond between the fully occupied Au 5*d* orbitals and the unoccupied π^* orbital of benzyl alkoxide (Fig. 4d). For benzyl alcohol oxidation on Au (Supplementary Fig. 23), OH* is generated at 0.856 V *vs.* RHE, in consistent with the CV test in Fig. 1a in the manuscript. The DFT results show that the RDS for benzyl alcohol oxidation over Au is the nucleophilic attack of Ph−C$(OH)_2$* on OH* (ΔG = 0.041 eV; Fig. 5b). Au would be oxidized to form AuO_x at working potential of >1.4 V that causes Au deactivation

For Au/CoOOH, DFT results indicate that benzyl alcohol prefers to adsorb at the interface of Au/CoOOH. Because of the electron transfer from Au to CoOOH, the Au atoms at Au/CoOOH interface are more electron-deficient, revealing oxidation state of +1. Therefore, the σ donation bond between the lone pair electrons of –CH_2O^- group and Au 6*s* orbital exists, together with the π bond between the π^* orbital of benzyl alcoholate and Au 5*d* orbitals, which in turn leads to the enrichment of benzyl alcohol on Au/CoOOH interface (detailed discussion *please see* Fig. 4 and Pages 8-10 in the manuscript). Under 0.85 - 1.2 V *vs.* RHE, OH* is only generated and adsorbed on Au. When the working potential is larger than 1.2 V *vs.* RHE, the OH* is mainly generated on CoOOH. After 1.5 V *vs.* RHE, Au is deactivated because of the generation of AuO_x. Under working potential 1.3 V *vs.* RHE, the adsorbed benzyl alcohol on Au/CoOOH interface is oxidized by the OH* generated on neighboring CoOOH. The DFT results show that the RDS for benzyl alcohol oxidation on Au/CoOOH is the generation of OH*

on CoOOH with a ΔG of −0.084 eV, which is smaller than the reaction barrier on Au (ΔG = 0.041 eV), giving rise to higher oxidation activity (Fig. 5b and the Supplementary Fig. 24). Therefore, the significantly enhanced current density of Au/CoOOH compared to pure Au is contributed by both the enrichment of benzyl alcohol at the interface and the higher oxidation activity. For pure CoOOH, although OH* can be generated at ~1.2 V *vs.* RHE, benzyl alcohol is weakly adsorbed on CoOOH, resulting in lower benzyl alcohol oxidation activity with higher oxidation onset potential of ~1.35 V *vs.* RHE.

Supplementary Note 8

To study whether the interaction between CoOOH and Au is special compared with other MOOH supports (NiOOH, FeOOH, NiFeOOH), we carried out XPS measurement of all the Au/MOOH samples. As shown in Supplementary Fig. 21, the Au 4*f* XPS spectra of Au/NiOOH, Au/FeOOH and Au/NiFeOOH samples exhibit positive peak shifting compared with pure Au, which is similar with the case of Au/CoOOH, suggesting electron transfer from Au to MOOH takes place in all the Au/MOOH samples. This is also confirmed by DFT results. The amount of electron transfer from Au to CoOOH, NiOOH, FeOOH, and NiFeOOH are 2.71, 1.67, 3.08, and 1.62 *e*, respectively (Supplementary Fig. 25a). These results suggest that there may be no significant difference in the electronic structure interaction between Au/CoOOH and other Au/MOOHs.

The adsorption behavior of benzyl alcohol in the form of alcoholate over Au NPs on different MOOH supports were also calculated by DFT. The adsorption energies of benzyl alcohol on the Au NPs in Au/MOOH were calculated to be comparable, namely –1.28, –1.10, –1.16, and –1.28 eV, respectively (Supplementary Fig. 25b), indicating that the difference of MOOH support on the adsorption energy between benzyl alcohol and Au is not obvious. This phenomenon can be understood by the following analysis. The adsorption between benzyl alcoholate and Au is composed of σ–π bonds, *i.e.*, σ bond between the lone-pair electron of –CH_2O^- in benzyl alcoholate and Au 6*s* orbital, and π bond between the π^* orbital of benzyl alcoholate and Au 5*d* orbital. Since the Au in Au/MOOH are electropositive, specifically the Au atoms at the Au/CoOOH interface are Au(I), the σ–π bonds between benzyl alcoholate and Au exists among all four Au/MOOHs. Based on the above results, we speculate that the significantly enhanced current density for Au/CoOOH compared to other Au/MOOHs samples

may not due to the special interaction between Au and CoOOH, but the unique catalytically active site over CoOOH that promotes the alcohol electrooxidation.

Supplementary Note 9

The model of Au/NiOOH was constructed in the similar way with that of Au/CoOOH. The space group of bulk γ-NiOOH is $R\bar{3}mH$, with the lattice parameters of $a = b = 2.82$ Å, $c = 20.65$ Å, $\alpha = \beta = 90°$, and $\gamma = 120°$. In order to determine the reaction active site on γ-NiOOH for oxidizing the benzyl alcohol, the Gibbs free energy changes (ΔG) of the generation of μ_1-OH, μ_2-OH, μ_3-OH, μ_1-O, μ_2-OH, and μ_3-OH were calculated. The μ_2-O, which can be generated on the (110) facet of γ-NiOOH, is determined to be the reaction active site for the oxidation of benzyl alcohol. Thus the (110) facet of bulk γ-NiOOH was cleaved, containing four layers of Ni atoms, O atoms, and H atoms, together with a vacuum layer of 15 Å. After that, the Au_{37} cluster was placed upon the (110) facet of NiOOH connecting with Au–O–Ni. The chemical formula of model Au/NiOOH is $Au_{37}Ni_{48}O_{102}H_{72}$.

For FeOOH, the space group of FeOOH is Pbnm, with the lattice parameters of $a = 4.6188$, $b = 9.9528$, $c = 3.0236$, and $\alpha = \beta = \gamma = 90°$. In order to determine the reaction active site on FeOOH for oxidizing the benzyl alcohol, the Gibbs free energy changes (ΔG) of the generation of μ_1-OH, μ_2-OH, μ_3-OH, μ_1-O, μ_2-OH, and μ_3-O were calculated. It is found that the μ_2-O generated on the (001) facet of FeOOH is the reaction active site for the oxidation of benzyl alcohol. After that, the (001) facet of FeOOH was cleaved, containing four layers of Fe atoms, eight layers of O atoms, three layers of H atoms, and a vacuum layer of 15 Å. Then, the Au_{37} cluster was placed upon the (001) facet of FeOOH binding with Au–O–Fe bond. The chemical formula of model Au/FeOOH is $Au_{37}Fe_{96}O_{192}H_{72}$.

The model of Au/NiFeOOH was constructed in a similar way with that of Au/NiOOH. The molar ratio of Ni : Fe in γ-NiFeOOH is 2 : 1 in this work. Therefore, it is supposed that the structure of γ-NiFeOOH is similar with that of γ-NiOOH. The (110) facet of γ-NiFeOOH was constructed by substituting one third of the Ni atoms with Fe atoms. In order to determine the reaction active site on γ-NiFeOOH for oxidizing the benzyl alcohol, the Gibbs free energy changes (ΔG) of the generation of μ_1-OH, μ_2-OH, μ_3-OH, μ_1-O, μ_2-OH, and μ_3-OH were calculated. The μ_2-O, which can be generated on the (110) facet of γ-NiOOH, is determined to be the reaction active site for the oxidation of benzyl alcohol. After that, the Au_{37} was placed

upon the (110) facet of NiFeOOH to construct the model of Au/NiFeOOH with the chemical formula of $Au_{37}Ni_{32}Fe_{16}O_{102}H_{72}$.

The mechanisms of benzyl alcohol oxidation to benzoic acid on Au/NiOOH, Au/FeOOH, and Au/NiFeOOH were calculated. The optimized geometries of the reaction intermediates for benzyl alcohol oxidation are displayed in Supplementary Figs. 29-31. The Gibbs free energy diagrams for the benzyl alcohol oxidation to benzoic acid on Au/NiOOH, Au/FeOOH, and Au/NiFeOOH are shown in Supplementary Fig. 32. The rate-determining step (RDS) are the generation of O* with the ΔG of 0.099 eV for Au/NiOOH, 0.042 eV for Au/FeOOH, and 0.112 eV for Au/NiFeOOH at 1.3 V *vs*. RHE, respectively, which are all larger than 0 eV. Therefore, Au/NiOOH, Au/FeOOH, and Au/NiFeOOH are inert in benzyl alcohol oxidation at 1.3 V *vs*. RHE, in accordance with the LSV curves (Supplementary Fig. 5).

Supplementary Note 10

The LUMO of methallyl alcohol and hydroxyacetone in the form of alkoxide are displayed in Supplementary Fig. 36. The LUMO of $CH_2{=}C(CH_3)CH_2O^-$ and $CH_3COCH_2O^-$ are delocalized, thus can be deduced to be π* orbital. The energy levels of the π* orbital for $CH_2{=}C(CH_3)CH_2O^-$ and $CH_3COCH_2O^-$ are −4.28 and −4.43 eV versus vacuum level, respectively, which are lower than part of the Au-*d* orbital. Therefore, the d-π* interaction between Au and $CH_2{=}C(CH_3)CH_2O^-/CH_3COCH_2O^-$ is reasonable.

Supplementary Note 11

The oxidation peaks in the CV curves in Supplementary Fig. 38 represent the oxidation of the different alcohols. In the anodic direction, the oxidation peaks from ~0.8-1.4 V *vs*. RHE correspond to the oxidation of ethanol, 1,3-propanediol and β-phenylethanol, respectively. At this potential range, the OH* species over Au (formed at ~0.8 V *vs*. RHE) or CoOOH (formed at ~1.2 V *vs*. RHE) exist which are capable of oxidizing alcohols. We carried out HPLC and NMR measurements to analyze the oxidation products. The electrooxidation were operated at constant potentials within 1.1~1.5 V *vs*. RHE for 1 h over Au/CoOOH catalyst in a 1 M KOH with 0.1 M alcohols. As shown in the Supplementary Fig. 39a, c, e, the HPLC spectra show that acetic acid (peak at 15.2 min), 3-hydroxypropionic acid (18.4min) and phenylacetic acid (7.1min) were observed, confirming the oxidation of ethanol, 1,3-propanediol and β-

phenylethanol, respectively. The formation of these products was also confirmed by NMR spectra (Supplementary Fig. 39b, d, f). The anodic current substantially decreased at 1.4 V *vs*. RHE because of the formation of catalytically inactive AuO_x species. The following peaks after 1.4 V *vs*. RHE of Au/CoOOH and Au/NiFeOOH are attributed to the alcohols oxidation over CoOOH or NiFeOOH (Supplementary Fig. 11). In the cathodic direction, another oxidation peak appeared at ~1.1 V vs. RHE for these alcohols, which is assigned to the regeneration of Au-OH species upon AuO_x reduction.

Supplementary Note 12

The electronic structures of benzyl alcohol, ethanol, 1,3–propanediol, *β*–phenylethanol, methallyl alcohol, and hydroxyacetone were calculated with the molecular orbital theory in the DMol^3 code in the Materials Studio version 5.5 software package[32,33]. In brief, these calculations were performed at the level of B3LYP[34] with the basis set of double numerical plus polarization. The Grimme method was used to treat the DFT-D correction. After that, the highest occupied molecular orbitals (HOMO) of benzyl alcohol, ethanol, 1,3–propanediol, *β*–phenylethanol, methallyl alcohol, and hydroxyacetone are calculated to be –6.458 eV, –7.497 eV, –7.256 eV, –6.701 eV, –6.689 eV, and –7.457 eV, respectively.

Supplementary Note 13

The mechanism of ethanol oxidation to acetic acid over Au/CoOOH was calculated. A series of models representing the reaction intermediates were constructed and denoted as $CH_3CH_2OH^*$ (ethanol does not exist in the form of alkoxide in this work because of its high pKa of 16), $CH_3CH_2O^*$, CH_3CHO^*, $CH_3CH(OH)_2^*$, $CH_3C(OH)_2^*$, and CH_3COOH^*, respectively. The optimized geometries of reaction intermediates for CH_3–CH_2OH oxidation are displayed in Supplementary Fig. 40. The corresponding Gibbs free energy diagram is shown in Supplementary Fig. 41. the ΔG of the electrophilic attack of electrophilic OH* on CH_3–CH_2OH is larger than 0 eV (0.488 eV) at 1.3 V *vs.* RHE, indicating that oxidation of CH_3–CH_2OH is slow to happen at 1.3 V *vs.* RHE. The CH_3–CH_2OH is difficult to be oxidized because of its low HOMO.

Supplementary References

1. Qi, J., *et al.* Electrocatalytic selective oxidation of glycerol to tartronate on Au/C anode catalysts in anion exchange membrane fuel cells with electricity cogeneration. *Appl. Catal. B.* **154**, 360–368 (2014).

2. Dai, C., *et al.* Electrochemical production of lactic acid from glycerol oxidation catalyzed by AuPt nanoparticles. *J. Catal.* **356**, 14–21 (2017).

3. Jin, C., *et al.* Platinum modification of gold and electrocatalytic oxidation of ethylene glycolon Pt-modified Au electrodes. *Electrochim. Acta.* **56**, 321–325 (2010).

4. Du, C., *et al.* Epitaxial growth of zigzag PtAu alloy surface on Au nano-pentagrams with enhanced Pt utilization and electrocatalytic performance toward ethanol oxidation reaction. *Electrochim. Acta.* **238**, 263–268 (2017).

5. Liu, Q., *et al.* Simple wet-chemical synthesis of core–shell Au–Pd@Pd nanocrystals and their improved electrocatalytic activity for ethylene glycol oxidation reaction. *Int. J. Hydrog. Energy.* **41**, 2547–2553 (2016).

6. Daşdelen, Z., *et al.* Preparation of anode catalysts for sorbitol electrooxidation based on the nanocomposites of fumed silica, reduced graphene oxide and gold nanoparticles. *Int. J. Hydrog. Energy.* **46**, 28121–28133 (2021).

7. Wu, E., *et al.* Synthesis of hollow echinus-like Au@PdAgNSs decorated reduced graphene oxide as an excellent electrocatalyst for enhanced ethanol electrooxidation. *J. Alloys Compd.* **789**, 174–182 (2019).

8. Zebulon, G., *et al.* Electrooxidation of glycerol on self-organized, mixed Au–Pt interfaces formed on Ni substrates. *J. Electrochem. Soc.* **167**, 056502 (2020).

9. Li, N., *et al.* Pt/C and Pd/C catalysts promoted by Au for glycerol and CO electrooxidation in alkaline medium. *J. Energy Inst.* **90**, 725–733 (2017).

10. Santos, J. B. C., *et al.* Promotional effect of auxiliary metals Bi on Pt, Pd, and Ag on Au, for glycerol electrolysis. *Int. J. Hydrog. Energy.* **45**, 25658–25671 (2020).

11. Xu, H., *et al.* Superior ethylene glycol electrocatalysis enabled by Au-decorated PdRu nanopopcorns. *J. Electroanal. Chem.* **814**, 31–37 (2018).

12. Wang, Z., *et al.* Free-standing nanoporous gold for direct plasmon enhanced electro-oxidation of alcohol molecules. *Nano Energy.* **56**, 286–293 (2019).

13. Habibi, E., *et al.* Glycerol electrooxidation on Pd, Pt and Au nanoparticles supported on carbon ceramic electrode in alkaline media. *Int. J. Hydrog. Energy.* **37**, 16800–16809 (2012).

14. Chadderdon, D. J., *et al.* Electrocatalytic oxidation of 5-hydroxymethylfurfural to 2, 5-furandicarboxylic acid on supported Au and Pd bimetallic nanoparticles. *Green Chem.* **16**, 3778–3786 (2014).

15. Song, H., *et al.* Hollow Au@ Pd and Au@ Pt core–shell nanoparticles as electrocatalysts for ethanol oxidation reactions. *J. Mater. Chem.* **22**, 25003–25010 (2012).

16. Xu, Y., *et al.* Evolution of 3D nanoporosity and morphology in selectively dealloying ternary $Au_{55}Cu_{25}Si_{20}$ metallic glass ribbon with enhanced alcohol electro-oxidation performance. *Nanoscale.* **10**, 18846–18856 (2018).

17. Xin, L., *et al.* Electrocatalytic oxidation of ethylene glycol (EG) on supported Pt and Au catalysts in alkaline media: Reaction pathway investigation in three-electrode cell and fuel cell reactors. *Appl. Catal. B.* **125**, 85–94 (2012).

18. Mahoney, E. G., *et al.* Analyzing the electrooxidation of ethylene glycol and glucose over platinum-modified gold electrocatalysts in alkaline electrolyte using in-situ infrared spectroscopy. *J. Power Sources.* **305**, 89–96 (2016).

19. Cao, X., *et al.* Highly strained au nanoparticles for improved electrocatalysis of ethanol oxidation reaction. *J. Phys. Chem. Lett.* **11**, 3005–3013 (2020).

20. Xu, H., *et al.* Self-supported porous 2D AuCu triangular nanoprisms as model electrocatalysts for ethylene glycol and glycerol oxidation. *J. Mater. Chem. A.* **5**, 15932–15939 (2017).

21. Lee, Y. W., *et al.* Synthesis and electrocatalytic activity of Au− Pd alloy nanodendrites for ethanol oxidation. *J. Phys. Chem. C.* **114**, 7689–7693 (2010).

22. Kardan, A., *et al.* In situ electrochemical activation as a generic strategy for promoting the electrocatalytic hydrogen evolution reaction and alcohol electro-oxidation in alkaline medium. *RSC Adv.* **11**, 10615–10624 (2021).

23. Zhang, Z., *et al.* Catalytic oxidation of carbohydrates into organic acids and furan chemicals. *Chem. Soc. Rev.* **47**, 1351–390 (2018).

24. Huang, Y., *et al.* Cobalt/iron (oxides) heterostructures for efficient oxygen evolution and benzyl alcohol oxidation reactions. *ACS Energy Lett.* **3**, 1854–1860 (2018).

25. Luo, L., *et al.* Selective activation of benzyl alcohol coupled with photoelectrochemical water oxidation via a radical relay strategy. *ACS Catal.* **10**, 4906–4913 (2020).

26. Wei, C., *et al.* Approaches for measuring the surface areas of metal oxide electrocatalysts for determining their intrinsic electrocatalytic activity. *Chem. Soc. Rev.* **48**, 2518 (2019).

27. Zheng, Y., *et al.* High electrocatalytic hydrogen evolution activity of an anomalous ruthenium catalyst. *J. Am. Chem. Soc.* **138**, 16174−16181 (2016).

28. Chang, C. R., *et al.* A water-promoted mechanism of alcohol oxidation on a Au (111) surface: understanding the catalytic behavior of bulk gold. *ACS Catal.* **3**, 1693−1699 (2013).

29. Zhang, Y. *et al.* Nano-gold catalysis in fine chemical synthesis. *Chem. Rev.* **112**, 2467–

2505 (2012).

30. Dionigi, F. *et al. In-situ* structure and catalytic mechanism of NiFe and CoFe layered double hydroxides during oxygen evolution. *Nat. Commun.* **11**, 2522 (2020).

31. Zhao, X. *et al*. Phenyl ring transfer mechanism of styrene selective oxidation to phenyl acetaldehyde on gold catalysts from density functional theory (DFT) studies. *J. Phys. Chem. C* 123, 1710–1719 (2019).

32. Delley, B. An all-electron numerical method for solving the local density functional for polyatomic molecules. *J. Chem. Phys.* **92**, 508–517 (1990).

33. Delley, B. From molecules to solids with the $DMol^3$ approach. *J. Chem. Phys.* **113**, 7756–7764 (2000).

34. Lee, C., Yang, W. & Parr, R. G. Development of the Colle–Salvetti correlation–energy formula into a functional of the electron density. *Phys. Rev. B* **37**, 785–789 (1988).
